# Properties and unbiased estimation of *F*- and *D*-statistics in samples containing related and inbred individuals

**DOI:** 10.1093/genetics/iyab090

**Published:** 2021-07-15

**Authors:** Mehreen R Mughal, Michael DeGiorgio

**Affiliations:** 1 Bioinformatics and Genomics at the Huck Institutes of the Life Sciences, Pennsylvania State University, University Park, PA 16802, USA; 2 Department of Computer and Electrical Engineering and Computer Science, Florida Atlantic University, Boca Raton, FL 33431, USA

**Keywords:** inbreeding, relatedness, introgression, demography, population genetics, gene flow

## Abstract

The Patterson *F*- and *D*-statistics are commonly used measures for quantifying population relationships and for testing hypotheses about demographic history. These statistics make use of allele frequency information across populations to infer different aspects of population history, such as population structure and introgression events. Inclusion of related or inbred individuals can bias such statistics, which may often lead to the filtering of such individuals. Here, we derive statistical properties of the *F*- and *D*-statistics, including their biases due to the inclusion of related or inbred individuals, their variances, and their corresponding mean squared errors. Moreover, for those statistics that are biased, we develop unbiased estimators and evaluate the variances of these new quantities. Comparisons of the new unbiased statistics to the originals demonstrates that our newly derived statistics often have lower error across a wide population parameter space. Furthermore, we apply these unbiased estimators using several global human populations with the inclusion of related individuals to highlight their application on an empirical dataset. Finally, we implement these unbiased estimators in open-source software package funbiased for easy application by the scientific community.

## Introduction

The recently introduced *F*- and *D*-statistics (Huson *et al.* 2005; Kulathinal *et al.* 2009; Reich *et al.* 2009; Green *et al.* 2010; Patterson *et al.* 2012) have transformed the way geneticists measure population differentiation. These statistics have been instrumental in many major recent discoveries, including testing which Neanderthal populations are closest to the populations that admixed with modern humans (Hajdinjak *et al.* 2018), and detecting which population is likely the admixing source for European admixture in modern Ethiopian populations (Molinaro *et al.* 2019). Iterating through different combinations of populations using the *F*_4_- and *D*-statistics has allowed reconstruction of population histories in diverse groups such as Native Americans and South Asians (Reich *et al.* 2012; Moorjani *et al.* 2013). In addition, the *D*-statistics have been used extensively to provide evidence of introgression and hybridization among species of *Drosophila* fruit flies and Heliconius butterflies (Martin *et al.* 2015; Turissini and Matute 2017).

In many cases, however, the populations tested by these statistics are small, and proper random sampling may include data from related individuals. It is common to remove one or more of the relatives from a group of related individuals because including them might provide a bias in the value of a particular statistic being measured (Rosenberg 2006; DeGiorgio and Rosenberg 2009; DeGiorgio *et al.* 2010; Harris and DeGiorgio 2017a; Waples and Anderson 2017). For this reason, we explore whether the current estimators for these statistics are biased with the inclusion of related or inbred individuals and if so, then develop unbiased estimators under such scenarios.

These statistics are flexible and relatively simple to compute, as they measure genetic drift along branches of a population tree by contrasting allele frequencies between different combinations of populations. Using allele frequency data from two, three, or four populations, these statistics measure shared variation along specific branches of the tree relating the populations. We begin by providing intuitive descriptions and formal definitions of each of the *F*- and *D*-statistics that we evaluate. Specifically, consider that we have allele frequency data at *J* biallelic loci from each of four populations, denoted *A*, *B*, *C*, and *D*. We denote the parametric population frequencies of the reference allele at locus *j* as *a_j_*, *b_j_*, *c_j_*, and *d_j_* in populations *A*, *B*, *C*, and *D*, respectively, which are unknown quantities that we will ultimately need to estimate prior to computing the *F*- and *D*-statistics from data. We begin by defining the true parametric population *F*- and *D*-statistics (Reich *et al.* 2009; Patterson *et al.* 2012) and then proceed in the *Theory* section to define sample estimators of these quantities, and show that our new estimators that account for related and inbred individuals reduce to the unbiased estimators in Appendix A of Patterson *et al.* (2012) when samples contain unrelated and noninbred individuals.

We first examine the *F*_2_ statistic, which measures the amount of genetic drift separating a pair of populations, and is thus a test for differentiation between them, and is akin to the widely used fixation index $F_{\text{ST}}$ (Weir and Cockerham 1984). For a pair of populations *A* and *B*, we define the *F*_2_ statistic as

(1) $$F_{2}(A,B)=\frac{1}{J}\sum_{j=1}^{J}F_{2}(A_{j},B_{j}),$$

where for locus *j*  

(2) $$F_{2}(A_{j},B_{j})={\left(a_{j}-b_{j}\right)}^{2}.$$

**Figure 1:**
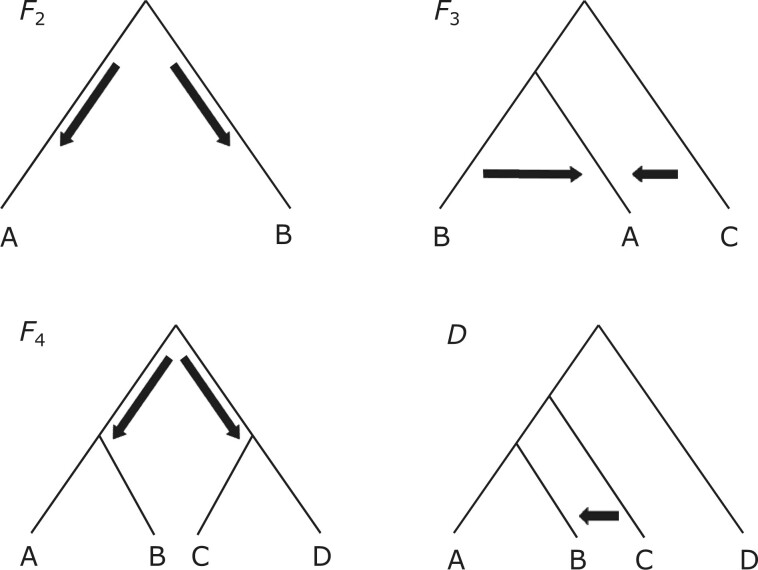
Trees showing the different relationships the *F*- and *D*-statistics are designed to test. $F_{2}(A,B)$ can test the differentiation of two populations *A* and *B*. $F_{3}(A;B,C)$ can test for introgression or relatedness between populations *A* and *B* or populations *A* and *C*. The placement of population *A* as an ingroup branch is arbitrary, because the $F_{3}(A;B,C)$ statistic measures the amount of genetic drift along branch *A*, and thus assumes the three-population tree is unrooted. However, because we are also showing how $F_{3}(A;B,C)$ can be employed to detect admixture in population *A*, we depict population *A* as an ingroup for visual convenience. $F_{4}(A,B;C,D)$ can test the hypothesis of whether two populations are closer to each other than they are to two other populations, in this case are *A* and *B* closer to each other than they are to *C* and *D*. The D(A,B,C,D) statistic can test whether there has been admixture between population *C* and either populations *A* or *B*.

It is clear from this definition that *F*_2_ takes values between zero, when the populations have identical allele frequencies, and one, when the populations are fixed for different alleles (Figure 1).

The *F*_3_ statistic employs allele frequencies from three populations, and measures the amount of genetic drift along the branch leading to a target population, given allele frequency data from two reference populations. For a target population *A* and two reference populations *B* and *C*, we define the *F*_3_ statistic as

(3) $$F_{3}(A;B,C)=\frac{1}{J}\sum_{j=1}^{J}F_{3}(A_{j};B_{j},C_{j}),$$

where for locus *j*  

(4) $$F_{3}(A_{j};B_{j},C_{j})=(a_{j}-b_{j})(a_{j}-c_{j}).$$

Because it measures genetic drift along a branch leading to a target population, its value is expected to be nonnegative. However, an interesting property of the *F*_3_ statistic is that it can be negative if the target population experienced admixture, and therefore a negative value directly indicates admixture in the history of the target population (Reich *et al.* 2009; Patterson *et al.* 2012). However, though *F*_3_ can detect admixture if its value is negative, admixture is not guaranteed to lead to negative values (Reich *et al.* 2009; Patterson *et al.* 2012), and it is therefore an inconclusive test for admixture if *F*_3_ is nonnegative. Moreover, because loci with higher minor allele frequencies may affect *F*_3_ more than loci with lower minor allele frequencies, the *F*_3_ statistic is sometimes normalized (Reich *et al.* 2009; Patterson *et al.* 2012) based on levels of diversity of the target population. Formally, this normalized *F*_3_ statistic has definition

(5) $$F_{3}(A;B,C|A)=\frac{F_{3}(A;B,C)}{2G(A)},$$

where we define for population *P* (here *P *=* A*)

(6) $$G(P)=\frac{1}{J}\sum_{j=1}G(P_{j})$$

such that for locus *j*  

(7) $$G(P_{j})=p_{j}(1-p_{j}).$$

The *F*_4_ statistic, on the other hand, is a test of “treeness” among a set of four populations, examining whether the unrooted tree relating four populations is supported by the allele frequencies within the set of populations. For a pair of sister populations *A* and *B* and a pair of sister populations *C* and *D*, we define the *F*_4_ statistic as

(8) $$F_{4}(A,B;C,D)=\frac{1}{J}\sum_{j=1}^{J}F_{4}(A_{j},B_{j};C_{j},D_{j}),$$

where for locus *j*  

(9) $$F_{4}(A_{j},B_{j};C_{j},D_{j})=(a_{j}-b_{j})(c_{j}-d_{j}).$$

If the unrooted relationship is true, then *F*_4_ takes the value of zero, and is nonzero otherwise. If it is known *a priori* that the unrooted relationship should be true, then a nonzero *F*_4_ statistic can be indicative of admixture, and the sign of the statistic will suggest which set of populations may be violating the assumed unrooted tree topology (Figure 1). As with the *F*_3_ statistic, a normalized version (Reich *et al.* 2009; Patterson *et al.* 2012) of the *F*_4_ statistic is sometimes used, with normalization based on the diversity of one of the four populations. Formally, this normalized *F*_4_ statistic has definition

(10) $$F_{4}(A,B;C,D|P)=\frac{F_{4}(A,B;C,D)}{G(P)}$$

where we normalize by diversity in population P∈{A,B,C,D}.

Finally, the *D*-statistic is a special version of the *F*_4_ statistic that is a test of treeness for a particular asymmetric rooted tree relating four populations, with the tree topology containing a pair of sister populations, together with a close and a distant outgroup population (Figure 1). For sister populations *A* and *B*, close outgroup population *C*, and distant outgroup population *D*, we define the *D* statistic as

(11) $$D(A,B,C,D)=-\frac{F_{4}(A,B;C,D)}{H(A,B,C,D)},$$

where

(12) $$H(A,B,C,D)=\frac{1}{J}\sum_{j=1}^{J}H(A_{j},B_{j},C_{j},D_{j})$$

is a normalizing factor to constrain the *D* statistic to take values between negative one and one, such that for locus *j*  

(13) $$H(A_{j},B_{j},C_{j},D_{j})=(a_{j}+b_{j}-2a_{j}b_{j})(c_{j}+d_{j}-2c_{j}d_{j}).$$

If the rooted relationship is true, then *D* takes the value of zero, and is nonzero otherwise. A nonzero *D* value can be used to detect admixture between the close outgroup population and one of the two sister populations based on its sign (Figure 1).

### Theory

The *F*- and *D*-statistic equations presented in the *Introduction* employ population allele frequencies, and are thus parameters of the set of populations. To estimate them, we first need to build an estimator of allele frequencies based on samples. We denote estimates of the reference allele frequencies at locus *j*, j=1,2,…,J, in populations *A*, *B*, *C*, and *D* by ${\hat{a}}_{j},\;{\hat{b}}_{j},\;{\hat{c}}_{j}$, and ${\hat{d}}_{j}$, respectively.

As used previously (*e.g.*, McPeek *et al.* 2004; DeGiorgio and Rosenberg 2009; DeGiorgio *et al.* 2010; Harris and DeGiorgio 2017a), a linear unbiased estimator of population reference allele frequency *p* at a biallelic locus can be defined as

(14) $$\hat{p}=\sum_{k=1}^{N(P)}\phi_{k}(P)X_{k},$$

where *N*(*P*) is the number of individuals sampled at the locus, *X_k_* is the frequency of the reference allele in individual *k* at the locus, and $\phi_{k}(P)$ is the weight of individual *k* in population *P* at the locus. McPeek *et al.* (2004) discussed the impact of various weighting schemes on allele frequency estimation, and Harris and DeGiorgio (2017a) examined the effects of weighting scheme on estimation of expected heterozygosity.

Plugging the sample estimate of allele frequencies in place of parametric population allele frequencies, estimators of the *F*- and *D*-statistics can be computed as

(15) $${\hat{F}}_{2}(A,B)=\frac{1}{J}\sum_{j=1}^{J}{\hat{F}}_{2}(A_{j},B_{j})$$

 

(16) $${\hat{F}}_{3}(A;B,C)=\frac{1}{J}\sum_{j=1}^{J}{\hat{F}}_{3}(A_{j};B_{j},C_{j})$$

 

(17) $${\hat{F}}_{4}(A,B;C,D)=\frac{1}{J}\sum_{j=1}^{J}{\hat{F}}_{4}(A_{j},B_{j};C_{j},D_{j})$$

 

(18) $${\hat{F}}_{3}(A;B,C|A)=\frac{{\hat{F}}_{3}(A;B,C)}{2\hat{G}(A)}$$

 

(19) $${\hat{F}}_{4}(A,B;C,D|P)=\frac{{\hat{F}}_{4}(A,B;C,D)}{\hat{G}(P)}$$

 

(20) $$\hat{D}(A,B,C,D)=-\frac{{\hat{F}}_{4}(A,B,C,D)}{\hat{H}(A,B,C,D)},$$

where

(21) $${\hat{F}}_{2}(A_{j},B_{j})={\left({\hat{a}}_{j}-{\hat{b}}_{j}\right)}^{2}$$

 

(22) $${\hat{F}}_{3}(A_{j},B_{j},C_{j})=({\hat{a}}_{j}-{\hat{b}}_{j})({\hat{a}}_{j}-{\hat{c}}_{j})$$

 

(23) $${\hat{F}}_{4}(A_{j},B_{j};C_{j},D_{j})=({\hat{a}}_{j}-{\hat{b}}_{j})({\hat{c}}_{j}-{\hat{d}}_{j}),$$

and where

(24) $$\hat{G}(P)=\frac{1}{J}\sum_{j=1}^{J}\hat{G}(P_{j})$$

 

(25) $$\hat{H}(A,B,C,D)=\frac{1}{J}\sum_{j=1}^{J}\hat{H}(A_{j},B_{j},C_{j},D_{j})$$

with

(26) $$\hat{G}(P_{j})={\hat{p}}_{j}(1-{\hat{p}}_{j})$$

 

(27) $$\hat{H}(A_{j},B_{j},C_{j},D_{j})=({\hat{a}}_{j}+{\hat{b}}_{j}-2{\hat{a}}_{j}{\hat{b}}_{j})({\hat{c}}_{j}+{\hat{d}}_{j}-2{\hat{c}}_{j}{\hat{d}}_{j}).$$

In the following, we discuss properties of these estimators, and where appropriate, develop unbiased estimators for the statistics that are biased due to the inclusion of related or inbred individuals in the sample.

To begin, we define the kinship coefficient $\Phi_{xy}$ between individuals *x* and *y*, as the probability that a pair of sampled alleles, one from *x* and one from *y* are identical by descent if x≠y, and as the probability that a pair of alleles sampled with replacement from individual *x* are identical by descent if *x *=* y* (Lange 2002). A pair of unrelated individuals *x* and *y* have kinship coefficient $\Phi_{xy}=0$ (Lange 2002). Moreover, an individual *x* with ploidy *m_x_* has kinship coefficient $\Phi_{xx}=1/m_{x}+(1-1/m_{x})f_{x}=(1/m_{x})[1+(m_{x}-1)f_{x}]$, where *f_x_* is the inbreeding coefficient of individual *x*, and is defined as the probability that a pair of alleles sampled without replacement in individual *x* are identical by descent (DeGiorgio *et al.* 2010). A noninbred individual *x* has inbreeding coefficient *f_x_* = 0, and so if *x* is noninbred, then their kinship coefficient is $\Phi_{xx}=1/m_{x}$. If an individual is haploid for regions of their genome, then by definition at those loci $\Phi_{xx}=1$. Directly accounting for the ploidy of individuals is especially pertinent for X-linked loci, as sampled male individuals will be haploid and sampled females will be diploid, thereby leading to noninbred male individuals to have self-kinship coefficients mirroring completely inbred individuals ($\Phi_{xx}=1$), whereas noninbred females at the same loci will have self-kinship coefficients consistent with noninbred diploids ($\Phi_{xx}=1/2$). As in DeGiorgio *et al.* (2010) and Harris and DeGiorgio (2017a), we define the weighted mean kinship coefficients across sets of individuals sampled in population P∈{A,B,C,D} at locus *j* as

(28) $$\Phi_{2}(P_{j})=\sum_{w=1}^{N(P_{j})}\sum_{x=1}^{N(P_{j})}\phi_{w}(P_{j})\phi_{x}(P_{j})\Phi_{wx}$$

 

(29) $$\Phi_{3}(P_{j})=\sum_{w=1}^{N(P_{j})}\sum_{x=1}^{N(P_{j})}\sum_{y=1}^{N(P_{j})}\phi_{w}(P_{j})\phi_{x}(P_{j})\phi_{y}(P_{j})\Phi_{\mathrm{wxy}}$$

 

(30) $$\Phi_{4}(P_{j})=\sum_{w=1}^{N(P_{j})}\sum_{x=1}^{N(P_{j})}\sum_{y=1}^{N(P_{j})}\sum_{z=1}^{N(P_{j})}\phi_{w}(P_{j})\phi_{x}(P_{j})\phi_{y}(P_{j})\phi_{z}(P_{j})\Phi_{\mathrm{wxyz}}$$

 

(31) $$\Phi_{2,2}(P_{j})=\sum_{w=1}^{N(P_{j})}\sum_{x=1}^{N(P_{j})}\sum_{y=1}^{N(P_{j})}\sum_{z=1}^{N(P_{j})}\phi_{w}(P_{j})\phi_{x}(P_{j})\phi_{y}(P_{j})\phi_{z}(P_{j})\Phi_{wx,yz},$$

which are the weighted mean kinship coefficients for the $N(P_{j})$ individuals sampled at locus *j* in population *P* for pairs, triples, quadruples, and pairs of pairs of individuals, respectively. Here, $\Phi_{\mathrm{wxy}},\;\Phi_{\mathrm{wxyz}}$, and $\Phi_{wx,yz}$ are kinship coefficients respectively defining the probabilities that a trio of alleles from individuals *w*, *x*, and *y*, a quadruple of alleles from individuals *w*, *x*, *y*, and *z*, and a pair of alleles from *w* and *x* and a pair of alleles from *y* and *z* are identical by descent (Harris 1964; Cockerham 1971). In particular, $\Phi_{wx},\;\Phi_{\mathrm{wxy}},\;\Phi_{\mathrm{wxyz}}$, and $\Phi_{wx,yz}$ are identical to the Cockerham (1971) coefficients denoted by *θ*, *γ*, *δ*, and Δ, respectively.

These Cockerham (1971) pairwise and extended kinship coefficients for measuring identity-by-descent probabilities among individuals are computed from sets of alleles sampled from two, three, or four individuals within the same population, whereas the *F*_2_, *F*_3_, and *F*_4_ statistics respectively are computed from sets of alleles sampled from two, three, or four distinct populations. Harris (1964) derived the genotypic covariances between individuals within a population, which are related to these kinship coefficients. In an interesting connection, $F_{2}(A,B)$ measures the covariance (variance) in the frequency difference between alleles sampled from populations *A* and *B*, $F_{3}(A;B,C)$ the covariance in the frequency difference between alleles sampled from populations *A* and *B* and the difference between alleles sampled from populations *A* and *C*, and $F_{4}(A,B;C,D)$ the covariance in the frequency difference between alleles sampled from populations *A* and *B* and the difference between alleles sampled from populations *C* and *D* (Peter 2016). For this reason, we may expect that these covariances (*F*-statistics) will depend on the identity-by-descent probabilities defined by the Cockerham (1971) kinship coefficients, which we show is the case based on our derivations of the theoretical properties of the *F*-statistics.

From our definitions of kinship, we know that unrelated individuals have kinship coefficients of zero, but noninbred individuals still have positive values of their self-kinship coefficient, thereby causing the mean kinship coefficients to necessarily be positive quantities. It is for this reason that some *F*-statistic estimators will be biased even without related or inbred individuals, and this bias would be due to finite sample size. The estimators presented in Appendix A of Patterson *et al.* (2012) correct this bias due to finite sample sizes, and our goal is to further correct for the biases induced by related and inbred individuals. For accurate estimates of the drift quantities, it is therefore important to obtain unbiased estimators.

A number of quantities (particularly variances and covariances involving the *F*- and *D*-statistics) will be mathematically complex, as they will involve linear combinations of higher order mean kinship coefficients. For this reason, we follow prior studies (DeGiorgio *et al.* 2010; Harris and DeGiorgio 2017a) and make the simplifying assumption that no individual in a sample from population *P* is related to more than one other individual in the sample, such that terms $\Phi_{3}(P_{j}),\;\Phi_{4}(P_{j}),\;\Phi_{2,2}(P_{j})$, and $\Phi_{2}{\left(P_{j}\right)}^{2}$ negligible to $\Phi_{2}(P_{j})$. Moreover, we assume that individuals sampled in different populations are unrelated to each other, as well as assume that the different populations in general are independent so that alleles sampled in different populations cannot be identical by descent. Furthermore, in the cases referred to in this article, sampling is defined as statistical sampling, where the expectation is averaging over repeated sampling. Under these assumptions, we approximate a few key results from prior studies (DeGiorgio *et al.* 2010; Harris and DeGiorgio 2017a) that will ultimately make derivations easier. Given that ${\hat{p}}_{j}$ is an estimate of the frequency of a reference allele at locus *j* in population *P*, we have the following expectations (approximate notation when not exact)

(32) $$E[{\hat{p}}_{j}]=p_{j}$$

 

(33) $$E[{\hat{p}}_{j}^{2}]=p_{j}^{2}+\Phi_{2}(P_{j})p_{j}(1-p_{j})$$

 

(34) $$E[{\hat{p}}_{j}^{3}]\approx p_{j}^{3}+3\Phi_{2}(P_{j})p_{j}^{2}(1-p_{j})$$

 

(35) $$E[{\hat{p}}_{j}^{4}]\approx p_{j}^{4}+6\Phi_{2}(P_{j})p_{j}^{3}(1-p_{j}).$$

From prior studies (Nei and Roychoudhury 1974; Weir 1989; DeGiorgio and Rosenberg 2009; DeGiorgio *et al.* 2010; Harris and DeGiorgio 2017a), we know that $2\hat{p}(1-\hat{p})$ is a downwardly biased estimator of expected heterozygosity at a locus, with the bias due to finite sample size (Nei and Roychoudhury 1974) and exacerbated by the inclusion of inbred (Weir 1989) and related (DeGiorgio and Rosenberg 2009; DeGiorgio *et al.* 2010; Harris and DeGiorgio 2017a) individuals in the sample. Based on this definition, 2G(P)=2p(1−p) is expected heterozygosity, and its estimator $2\hat{G}(P)=2\hat{p}(1-\hat{p})$ therefore biased. We begin by developing an unbiased estimator for *G*(*P*), as it is a key normalization quantity in the *F*_3_ and *F*_4_ statistics.

Lemma 1. Consider *J* polymorphic loci in a population *P* with parametric reference allele frequencies $p_{j}\in (0,1)$, and suppose we take a random sample of $N(P_{j})$ individuals at locus *j*, some of which may be related or inbred. The estimator $\hat{G}(P)$ has downward bias

(36) $$\text{Bias}[\hat{G}(P)]=-\frac{1}{J}\sum_{j=1}^{J}\Phi_{2}(P_{j})G(P_{j})$$

and an unbiased estimator of *G*(*P*) is

(37) $$\tilde{G}(P)=\frac{1}{J}\sum_{j=1}^{J}\tilde{G}(P_{j}),$$

where

(38) $$\tilde{G}(P_{j})=\frac{\hat{G}(P_{j})}{1-\Phi_{2}(P_{j})}$$

is an unbiased estimator of $G(P_{j})$.

Though this result is also given based on work in page 153 of Weir (1996), we provide the proof of Lemma 1 in the Appendix for completeness. Intuitively though, because $\hat{G}(P)$ involves the product of frequencies for two alleles drawn from population *P*, there is a chance of having the two alleles being identical by descent by sampling the same allele twice, and is therefore a biased estimator with and without related or inbred individuals. As a corollary, we next provide the bias of $\hat{G}(P)$ due to finite sample size, and use it to construct the unbiased estimator $\overset{⌣}{G}(P)$ of *G*(*P*) in samples of unrelated and noninbred individuals (proof of this corollary given in the Appendix). Note that this estimator is identical to the one provided in Appendix A of Patterson *et al.* (2012).

Corollary 2. Consider *J* polymorphic loci in a population *P* with parametric reference allele frequencies $p_{j}\in (0,1)$, and suppose we take a random sample of $N(P_{j})$ unrelated and noninbred individuals at locus *j* where individual $k\in \{1,2,\ldots ,N(P_{j})$} has ploidy *m_k_*. Assuming allele frequencies are estimated using the sample proportion, the estimator $\hat{G}(P)$ has downward bias

(39) $$\text{Bias}[\hat{G}(P)]=-\frac{1}{J}\sum_{j=1}^{J}\frac{1}{\sum_{k=1}^{N(P_{j})}m_{k}}G(P_{j})$$

and an unbiased estimator of *G*(*P*) is

(40) $$\overset{⌣}{G}(P)=\frac{1}{J}\sum_{j=1}^{J}\overset{⌣}{G}(P_{j}),$$

where

(41) $$\overset{⌣}{G}(P_{j})=\frac{\sum_{k=1}^{N(P_{j})}m_{k}}{\sum_{k=1}^{N(P_{j})}m_{k}-1}\hat{G}(P_{j})$$

is an unbiased estimator of $G(P_{j})$. Here, $\overset{⌣}{G}(P_{j})$ is equivalent to the unbiased estimator termed ${\hat{h}}_{A}$ in Appendix A of Patterson *et al.* (2012).

We next consider examining the bias of the estimator ${\hat{F}}_{2}(A,B)$. As with $\hat{G}(P)$, because ${\hat{F}}_{2}(A,B)$ requires sampling two alleles from population *A* and two alleles from population *B*, we find it is biased due to the inclusion of related or inbred individuals. We present the formal result for *F*_2_ next (Proposition 3), and prove the result in the Appendix.

Proposition 3. Consider *J* polymorphic loci in populations *A* and *B* with respective parametric reference allele frequencies $a_{j},b_{j}\in (0,1)$, and suppose we take a random sample of $N(P_{j})$ individuals at locus *j* in population P∈{A,B}, some of which may be related or inbred. The estimate ${\hat{F}}_{2}(A,B)$ has upward bias

(42) $$\text{Bias}[{\hat{F}}_{2}(A,B)]=\frac{1}{J}\sum_{j=1}^{J}[\Phi_{2}(A_{j})G(A_{j})+\Phi_{2}(B_{j})G(B_{j})]$$

and an unbiased estimator of $F_{2}(A,B)$ is

(43) $${\tilde{F}}_{2}(A,B)=\frac{1}{J}\sum_{j=1}^{J}[{\hat{F}}_{2}(A_{j},B_{j})-\Phi_{2}(A_{j})\tilde{G}(A_{j})-\Phi_{2}(B_{j})\tilde{G}(B_{j})].$$

As one can see, the estimator ${\hat{F}}_{2}(A,B)$ is upwardly biased due to relatedness and inbreeding, and that sampling within both populations *A* and *B* contributes proportionally to this bias. The new unbiased estimator ${\tilde{F}}_{2}(A,B)$ corrects this bias by adjusting the computation to account for the kinship coefficients and diversity within each population, with the adjustment of diversity using the unbiased estimator $\tilde{G}(P)$ presented in Lemma 1. As a corollary, we next provide the bias of ${\hat{F}}_{2}(A,B)$ due to finite sample size, and use it to construct the unbiased estimator ${\overset{⌣}{F}}_{2}(A,B)$ of $F_{2}(A,B)$ in samples of unrelated and noninbred individuals (proof of this corollary given in the Appendix). Note that this estimator is identical to the one derived in Appendix A of Patterson *et al.* (2012), and we provide an additional corollary highlighting its bias in samples containing related or inbred individuals, which we prove in the Appendix.

Corollary 4. Consider *J* polymorphic loci in populations *A* and *B* with respective parametric reference allele frequencies $a_{j},b_{j}\in (0,1)$, and suppose we take a random sample of $N(P_{j})$ unrelated and noninbred individuals at locus *j* in population P∈{A,B} where individual $k\in \{1,2,\ldots ,N(P_{j})$} has ploidy *m_k_*. Assuming allele frequencies are estimated using the sample proportion, the estimate ${\hat{F}}_{2}(A,B)$ has upward bias

(44) $$\text{Bias}[{\hat{F}}_{2}(A,B)]=\frac{1}{J}\sum_{j=1}^{J}[\frac{1}{\sum_{k=1}^{N(A_{j})}m_{k}}G(A_{j})+\frac{1}{\sum_{k=1}^{N(B_{j})}m_{k}}G(B_{j})]$$

and an unbiased estimator of $F_{2}(A,B)$ is

(45) $${\overset{⌣}{F}}_{2}(A,B)=\frac{1}{J}\sum_{j=1}^{J}[{\hat{F}}_{2}(A_{j},B_{j})-\frac{1}{\sum_{k=1}^{N(A_{j})}m_{k}-1}\hat{G}(A_{j})-\frac{1}{\sum_{k=1}^{N(B_{j})}m_{k}-1}\hat{G}(B_{j})].$$

Here, ${\overset{⌣}{F}}_{2}(A_{j},B_{j})$ is equivalent to the unbiased estimator termed ${\hat{F}}_{2}(A,B)$ in Appendix A of Patterson *et al.* (2012).

Corollary 5. Consider *J* polymorphic loci in populations *A* and *B* with respective parametric reference allele frequencies $a_{j},b_{j}\in (0,1)$, and suppose we take a random sample of $N(P_{j})$ individuals at locus *j* in population P∈{A,B}, some of which may be related or inbred. The estimate ${\overset{⌣}{F}}_{2}(A,B)$ described in Corollary 4 [also in Appendix A of Patterson *et al.* (2012)] has upward bias

(46) $$\text{Bias}[{\overset{⌣}{F}}_{2}(A,B)]=\frac{1}{J}\sum_{j=1}^{J}[\frac{\Phi_{2}(A_{j})\sum_{k=1}^{N(A_{j})}m_{k}-1}{\sum_{k=1}^{N(A_{j})}m_{k}-1}G(A_{j})+\frac{\Phi_{2}(B_{j})\sum_{k=1}^{N(B_{j})}m_{k}-1}{\sum_{k=1}^{N(B_{j})}m_{k}-1}G(B_{j})].$$

Similarly to ${\hat{F}}_{2}(A,B)$, the original estimator ${\hat{F}}_{3}(A;B,C)$ is also upwardly biased because it requires the sampling of two alleles from the target population *A*. We show the formal results for *F*_3_ next (Proposition 6), and prove the result in the Appendix.

Proposition 6. Consider *J* polymorphic loci in populations *A*, *B*, and *C* with respective parametric reference allele frequencies $a_{j},b_{j},c_{j}\in (0,1)$, and suppose we take a random sample of $N(P_{j})$ individuals at locus *j* in population P∈{A,B,C}, some of which may be related or inbred. The estimate ${\hat{F}}_{3}(A;B,C)$ has upward bias

(47) $$\text{Bias}[{\hat{F}}_{3}(A;B,C)]=\frac{1}{J}\sum_{j=1}^{J}\Phi_{2}(A_{j})G(A_{j})$$

and an unbiased estimator of $F_{3}(A;B,C)$ is

(48) $${\tilde{F}}_{3}(A;B,C)=\frac{1}{J}\sum_{j=1}^{J}[{\hat{F}}_{3}(A_{j};B_{j},C_{j})-\Phi_{2}(A_{j})\tilde{G}(A_{j})].$$

The bias of the original estimator is proportional to the relatedness and diversity within the target population *A*. The new unbiased estimator ${\tilde{F}}_{3}(A;B,C)$ corrects the bias by adjusting the computation to account for the kinship and diversity within the target population, with the adjustment of diversity using the unbiased estimator $\tilde{G}(A)$. Moreover, it is important to note that the reference populations *B* and *C* do not contribute to bias, as only a single allele is sampled from each of these populations. As a corollary, we next provide the bias of ${\hat{F}}_{3}(A;B,C)$ due to finite sample size, and use it to construct the unbiased estimator ${\overset{⌣}{F}}_{3}(A;B,C)$ of $F_{3}(A;B,C)$ in samples of unrelated and noninbred individuals (proof of this corollary given in the Appendix). Note that this estimator is identical to the one derived in Appendix A of Patterson *et al.* (2012), and we provide an additional corollary highlighting its bias in samples containing related or inbred individuals, which we prove in the Appendix.

Corollary 7. Consider *J* polymorphic loci in populations *A*, *B*, and *C* with respective parametric reference allele frequencies $a_{j},b_{j},c_{j}\in (0,1)$, and suppose we take a random sample of $N(P_{j})$ unrelated and noninbred individuals at locus *j* in population P∈{A,B,C} where individual $k\in \{1,2,\ldots ,N(P_{j})$} has ploidy *m_k_*. Assuming allele frequencies are estimated using the sample proportion, the estimate ${\hat{F}}_{3}(A;B,C)$ has upward bias

(49) $$\text{Bias}[{\hat{F}}_{3}(A;B,C)]=\frac{1}{J}\sum_{j=1}^{J}\frac{1}{\sum_{k=1}^{N(A_{j})}m_{k}}G(A_{j})$$

and an unbiased estimator of $F_{3}(A;B,C)$ is

(50) $${\overset{⌣}{F}}_{3}(A;B,C)=\frac{1}{J}\sum_{j=1}^{J}[{\hat{F}}_{3}(A_{j};B_{j},C_{j})-\frac{1}{\sum_{k=1}^{N(A_{j})}m_{k}-1}\hat{G}(A_{j})].$$

Here, ${\overset{⌣}{F}}_{3}(A_{j};B_{j},C_{j})$ is equivalent to the unbiased estimator termed ${\hat{F}}_{3}(A;B,C)$ in Appendix A of Patterson *et al.* (2012).

Corollary 8. Consider *J* polymorphic loci in populations *A*, *B*, and *C* with respective parametric reference allele frequencies $a_{j},b_{j},c_{j}\in (0,1)$, and suppose we take a random sample of $N(P_{j})$ individuals at locus *j* in population P∈{A,B,C}, some of which may be related or inbred. The estimate ${\overset{⌣}{F}}_{3}(A;B,C)$ described in Corollary 7 [also in Appendix A of Patterson *et al.* (2012)] has upward bias

(51) $$\text{Bias}[{\overset{⌣}{F}}_{3}(A;B,C)]=\frac{1}{J}\sum_{j=1}^{J}\frac{\Phi_{2}(A_{j})\sum_{k=1}^{N(A_{j})}m_{k}-1}{\sum_{k=1}^{N(A_{j})}m_{k}-1}G(A_{j}).$$

Given that ${\hat{F}}_{3}(A;B,C|A)$ uses the biased estimators ${\hat{F}}_{3}(A;B,C)$ and $\hat{G}(A)$ in its definition, we can expect that it would be biased as its component estimators are biased, and these components have different biases that are also in different directions. However, ${\hat{F}}_{3}(A;B,C|A)$ is a ratio estimator, and we can therefore not directly take its expectation to evaluate bias. Instead, we will make some simplifying assumptions and compute the approximate bias of ${\hat{F}}_{3}(A;B,C|A)$. We show the formal results next (Proposition 9), and prove the result in the Appendix.

Proposition 9. Consider *J* polymorphic loci in populations *A*, *B*, and *C* with respective parametric reference allele frequencies $a_{j},b_{j},c_{j}\in (0,1)$, and suppose we take a random sample of $N(P_{j})$ individuals at locus *j* in population P∈{A,B,C}, some of which may be related or inbred. The ratio estimator ${\hat{F}}_{3}(A;B,C|A)$ is approximately upwardly biased, assuming that its mean is well-approximated by the ratio of means of ${\hat{F}}_{3}(A;B,C)$ and $2\hat{G}(A)$ that it uses in its definition, with its upward approximate bias

(52) $$\text{Bias}[{\hat{F}}_{3}(A;B,C|A)]\approx \frac{\left(1/J)\sum_{j=1}^{J}\Phi_{2}(A_{j})G(A_{j}\right)}{G(A)-(1/J)\sum_{j=1}^{J}\Phi_{2}(A_{j})G(A_{j})}[F_{3}(A;B,C|A)+\frac{1}{2}].$$

Moreover, an approximately unbiased estimator of $F_{3}(A;B,C|A)$ is

(53) $${\tilde{F}}_{3}(A;B,C|A)=\frac{{\tilde{F}}_{3}(A;B,C)}{2\tilde{G}(A)}.$$

There is an upward approximate bias of the original normalized *F*_3_ estimator, and the bias is, as with the standard estimator of *F*_3_, due partially to the diversity and sampling in the target population. The new approximately unbiased estimator ${\tilde{F}}_{3}(A;B,C|A)$ is based simply on the ratio of unbiased estimators of its components ${\tilde{F}}_{3}(A;B,C)$ and $\tilde{G}(A)$. As a corollary, we next provide the approximate bias of ${\hat{F}}_{3}(A;B,C|A)$ due to finite sample size, and use it to construct the approximately unbiased estimator ${\overset{⌣}{F}}_{3}(A;B,C|A)$ of $F_{3}(A;B,C|A)$ in samples of unrelated and noninbred individuals (proof of this corollary given in the Appendix). We provide an additional corollary highlighting its bias in samples containing related or inbred individuals, which we prove in the Appendix.

Corollary 10. Consider *J* polymorphic loci in populations *A*, *B*, and *C* with respective parametric reference allele frequencies $a_{j},b_{j},c_{j}\in (0,1)$, and suppose we take a random sample of $N(P_{j})$ unrelated and noninbred individuals at locus *j* in population P∈{A,B,C} where individual $k\in \{1,2,\ldots ,N(P_{j})$} has ploidy *m_k_*. The ratio estimator ${\hat{F}}_{3}(A;B,C|A)$ is approximately upwardly biased, assuming that its mean is well-approximated by the ratio of means of ${\hat{F}}_{3}(A;B,C)$ and $2\hat{G}(A)$ that it uses in its definition, with its upward approximate bias

(54) $$\text{Bias}[{\hat{F}}_{3}(A;B,C|A)]\approx \frac{\left(1/J)\sum_{j=1}^{J}[1/\sum_{k=1}^{N(A_{j})}m_{k}]G(A_{j}\right)}{G(A)-(1/J)\sum_{j=1}^{J}[1/\sum_{k=1}^{N(A_{j})}m_{k}]G(A_{j})}[F_{3}(A;B,C|A)+\frac{1}{2}].$$

Moreover, an approximately unbiased estimator of $F_{3}(A;B,C|A)$ is

(55) $${\overset{⌣}{F}}_{3}(A;B,C|A)=\frac{{\overset{⌣}{F}}_{3}(A;B,C)}{2\overset{⌣}{G}(A)}.$$

Corollary 11. Consider *J* polymorphic loci in populations *A*, *B*, and *C* with respective parametric reference allele frequencies $a_{j},b_{j},c_{j}\in (0,1)$, and suppose we take a random sample of $N(P_{j})$ individuals at locus *j* in population P∈{A,B,C}, some of which may be related or inbred. The ratio estimate ${\overset{⌣}{F}}_{3}(A;B,C|A)$ described in Corollary 10 is approximately upwardly biased, assuming that its mean is well-approximated by the ratio of means of ${\overset{⌣}{F}}_{3}(A;B,C)$ and $2\overset{⌣}{G}(A)$ that it uses in its definition, with its upward approximate bias

(56) $$\text{Bias}[{\overset{⌣}{F}}_{3}(A;B,C|A)]\approx \frac{1}{2}[\frac{1}{Y(A)}-\frac{1}{G(A)}]F_{3}(A;B,C)+\frac{X(A)}{2Y(A)},$$

where

(57) $$X(A)=\frac{1}{J}\sum_{j=1}^{J}\frac{\Phi_{2}(A_{j})\sum_{k=1}^{N(A_{j})}m_{k}-1}{\sum_{k=1}^{N(A_{j})}m_{k}-1}G(A_{j})$$

 

(58) $$Y(A)=\frac{1}{J}\sum_{j=1}^{J}\frac{[1-\Phi_{2}(A_{j})]\sum_{k=1}^{N(A_{j})}m_{k}}{\sum_{k=1}^{N(A_{j})}m_{k}-1}G(A_{j}).$$

Finally, we move to the four population statistics *F*_4_ and *D*. Note that the *F*_4_ statistic by definition only samples a single allele per population, and therefore the original estimator ${\hat{F}}_{4}(A,B;C,D)$ is intuitively unbiased. We show the formal results next (Proposition 12), and prove the result in the Appendix.

Proposition 12. Consider *J* polymorphic loci in populations *A*, *B*, *C*, and *D* with respective parametric reference allele frequencies $a_{j},b_{j},c_{j},d_{j}\in (0,1)$, and suppose we take a random sample of $N(P_{j})$ individuals at locus *j* in population P∈{A,B,C,D}, some of which may be related or inbred. The estimator ${\hat{F}}_{4}(A,B;C,D)$ is unbiased.

Though the original *F*_4_ estimator is unbiased, the normalized *F*_4_ and *D* statistics are more complicated as they are ratio estimators, meaning their biases cannot be directly assessed. However, intuitively, because both estimators have ${\hat{F}}_{4}(A,B;C,D)$ as their numerator, bias would seemingly derive from their denominator component. Next, we show formally in Proposition 13 that the normalized ${\hat{F}}_{4}(A,B;C,D|P)$ estimator is approximately upwardly biased, and prove the result in the Appendix.

Proposition 13. Consider *J* polymorphic loci in populations *A*, *B*, *C* and *D* with respective parametric reference allele frequencies $a_{j},b_{j},c_{j},d_{j}\in (0,1)$, and suppose we take a random sample of $N(P_{j})$ individuals at locus *j* in population P∈{A,B,C,D}, some of which may be related or inbred. The ratio estimator ${\hat{F}}_{4}(A,B;C,D|P)$ is approximately upwardly biased, assuming that its mean is well-approximated by the ratio of means of ${\hat{F}}_{4}(A,B;C,D)$ and $\hat{G}(P)$ for any population P∈{A,B,C,D} that it uses in its definition, with its upward approximate bias

(59) $$\text{Bias}[{\hat{F}}_{4}(A,B;C,D|P)]\approx \frac{\left(1/J)\sum_{j=1}^{J}\Phi_{2}(P_{j})G(P_{j}\right)}{G(P)-(1/J)\sum_{j=1}^{J}\Phi_{2}(P_{j})G(P_{j})}F_{4}(A,B;C,D|P).$$

Moreover, an approximately unbiased estimator of $F_{4}(A,B;C,D|P)$ is

(60) $${\tilde{F}}_{4}(A,B;C,D|P)=\frac{{\hat{F}}_{4}(A,B;C,D)}{\tilde{G}(P)}.$$

The reasoning that the ${\hat{F}}_{4}(A,B;C,D|P)$ estimator has upward approximate bias is that its estimator $\hat{G}(P)$ used in its denominator is downwardly biased. By using the unbiased estimator $\tilde{G}(P)$ in its place within the denominator, we find a new estimator ${\tilde{F}}_{4}(A,B;C,D|P)$ is approximately unbiased. As a corollary, we next provide the approximate bias of ${\hat{F}}_{4}(A,B;C,D|P)$ due to finite sample size, and use it to construct the approximately unbiased estimator ${\overset{⌣}{F}}_{4}(A,B;C,D|P)$ of $F_{4}(A,B;C,D|P)$ in samples of unrelated and noninbred individuals (proof of this corollary given in the Appendix). We provide an additional corollary highlighting its bias in samples containing related or inbred individuals, which we prove in the Appendix.

Corollary 14. Consider *J* polymorphic loci in populations *A*, *B*, *C*, and *D* with respective parametric reference allele frequencies $a_{j},b_{j},c_{j},d_{j}\in (0,1)$, and suppose we take a random sample of $N(P_{j})$ unrelated and noninbred individuals at locus *j* in population P∈{A,B,C,D} where individual $k\in \{1,2,\ldots ,N(P_{j})$} has ploidy *m_k_*. The ratio estimator ${\hat{F}}_{4}(A,B;C,D|P)$ is approximately upwardly biased, assuming that its mean is well-approximated by the ratio of means of ${\hat{F}}_{4}(A,B;C,D)$ and $\hat{G}(P)$ that it uses in its definition, with its upward approximate bias

(61) $$\text{Bias}[{\hat{F}}_{4}(A,B;C,D|P)]\approx \frac{\left(1/J)\sum_{j=1}^{J}[1/\sum_{k=1}^{N(P_{j})}m_{k}]G(P_{j}\right)}{G(P)-(1/J)\sum_{j=1}^{J}[1/\sum_{k=1}^{N(P_{j})}m_{k}]G(P_{j})}F_{4}(A,B;C,D).$$

Moreover, an approximately unbiased estimator of $F_{4}(A,B;C,D|P)$ is

(62) $${\overset{⌣}{F}}_{4}(A,B;C,D|P)=\frac{{\hat{F}}_{4}(A,B;C,D)}{\overset{⌣}{G}(P)}.$$

Corollary 15. Consider *J* polymorphic loci in populations *A*, *B*, *C*, and *D* with respective parametric reference allele frequencies $a_{j},b_{j},c_{j},d_{j}\in (0,1)$, and suppose we take a random sample of $N(P_{j})$ individuals at locus *j* in population P∈{A,B,C,D}, some of which may be related or inbred. The ratio estimate ${\overset{⌣}{F}}_{4}(A,B;C,D|P)$ described in Corollary 14 is approximately upwardly biased, assuming that its mean is well-approximated by the ratio of means of ${\hat{F}}_{4}(A,B;C,D)$ and $\overset{⌣}{G}(P)$ that it uses in its definition, with its upward approximate bias

(63) $$\text{Bias}[{\overset{⌣}{F}}_{4}(A,B;C,D|P)]\approx [\frac{1}{Y(P)}-\frac{1}{G(P)}]F_{4}(A,B;C,D),$$

where

(64) $$Y(P)=\frac{1}{J}\sum_{j=1}^{J}\frac{[1-\Phi_{2}(P_{j})]\sum_{k=1}^{N(P_{j})}m_{k}}{\sum_{k=1}^{N(P_{j})}m_{k}-1}G(P_{j}).$$

The bias property of the *D* statistic is different than the normalized *F*_4_ statistic, as the estimator $\hat{H}(A,B,C,D)$ of its denominator is unbiased (Lemma 17 of the Appendix). Intuitively, this result is due to the denominator not having a product of frequencies for two alleles sampled from the same population. Because both its numerator and denominator are unbiased, we next show that the ratio estimator $\hat{D}(A,B,C,D)$ is approximately unbiased in Proposition 16, and prove the result in the Appendix.

Proposition 16. Consider *J* polymorphic loci in populations *A*, *B*, *C*, and *D* with respective parametric reference allele frequencies $a_{j},b_{j},c_{j},d_{j}\in (0,1)$, and suppose we take a random sample of $N(P_{j})$ individuals at locus *j* in population P∈{A,B,C,D}, some of which may be related or inbred. The ratio estimator $\hat{D}(A,B,C,D)$ is approximately unbiased, assuming that its mean is well-approximated by the ratio of means of $-{\hat{F}}_{4}(A,B;C,D)$ and $\hat{H}(A,B,C,D)$ that it uses in its definition.

In addition to bias, variance is an important property of an estimator, as both bias and variance are components of mean squared error (MSE). Because the formulas and derivations for the variances of the *F*- and *D*-statistics are not particularly insightful, we relegate these results to the Appendix. Specifically, we provide the variances for ${\hat{F}}_{2}(A,B),\;{\tilde{F}}_{2}(A,B)$, ${\overset{⌣}{F}}_{2}(A,B)$, ${\hat{F}}_{3}(A;B,C),\;{\tilde{F}}_{3}(A;B,C)$, ${\overset{⌣}{F}}_{3}(A;B,C)$, ${\hat{F}}_{3}(A;B,C|A),\;{\tilde{F}}_{3}(A;B,C|A)$, ${\overset{⌣}{F}}_{3}(A;B,C|A)$, ${\hat{F}}_{4}(A,B;C,D),\;{\hat{F}}_{4}(A,B;C,D|P),\;{\tilde{F}}_{4}(A,B;C,D|P)$, ${\overset{⌣}{F}}_{4}(A,B;C,D|P)$, and $\hat{D}(A,B,C,D)$ in Propositions 20, 21, 22, 23, 25, 26, 28, 31, 32, 27, 34, 37, 38, and 41 of the Appendix, respectively.

## Results

In the *Theory* and Appendix, we introduced new unbiased estimators of *F*_2_ and *F*_3_ statistics, and derived biases and variances (and hence MSEs) for the original and new estimators of *F*- and *D*-statistics. In this section, we theoretically evaluate the relative performances of the old biased estimators and the new unbiased estimators under an array of settings, including different mixtures of relatedness, inbreeding, sample sizes, and population parameters.

For all of our results we require the kinship coefficients for each pair of individuals. To acquire these values, we need to know if each individual is related to any other in the population and also whether they are inbred, and if so, how these values are quantified through the use of kinship coefficients ($\Phi_{xy}$). To summarize how an entire sample from a population *P* is related to each other at a locus, we use

(65) $$\Phi_{2}(P)=\sum_{w=1}^{N(P)}\sum_{x=1}^{N(P)}\phi_{w}(P)\phi_{x}(P)\Phi_{wx},$$

where $\phi_{w}(P)$ and $\phi_{x}(P)$ are weights of individuals *w* and *x* in population *P*, and in this study we use weights corresponding to the proportion of alleles contributed by individual *x* to the sample from population *P*, which is computed as

(66) $$\phi_{x}(P)=\frac{m_{x}}{\sum_{k=1}^{N(P)}m_{k}}.$$

Here *m_x_* is the ploidy of individual *x*. Moreover, using this weighting scheme, we also estimate the frequency of the reference allele at a biallelic locus as the sample proportion (McPeek *et al.* 2004; DeGiorgio *et al.* 2010; Harris and DeGiorgio, 2017a)

(67) $$\hat{p}=\sum_{k=1}^{N(P)}\phi_{k}(P)X_{k}=\sum_{k=1}^{N(P)}\frac{m_{k}}{\sum_{j=1}^{N(P)}m_{j}}X_{k}.$$

### Effect of population *F*-statistic value on mean squared error

The relationship between the population parameter for a statistic and the estimate based on a sample from the population is important to evaluate. We compare the difference in the MSE between the biased $\hat{F}$ estimators, the $\overset{⌣}{F}$ estimators, which are unbiased in samples not containing related or inbred individuals, and our unbiased $\tilde{F}$ estimators to the true value of each statistic in the cases for which both estimators exist. The *F*_2_, *F*_3_, and *F*_4_ statistics require allele frequency information from either two, three, or four populations, respectively.

For our *F*_2_ comparisons, we use the sample allele frequencies from the YRI (sub-Saharan African) and CEU (central Europeans) from the 1000 Genomes Project (The 1000 Genomes Project Consortium 2015) as the true population allele frequencies to obtain the true $F_{2}(A,B)$ statistic by using the population definition from the *Introduction*, with populations A=CEU and B=YRI. We use populations from the 1000 Genomes Project, as the released dataset of genotype calls across the 2504 worldwide samples does not include related individuals. To evaluate the relative performances of *F*_2_ estimators over a range of true *F*_2_ values, we randomly sample 20 independent loci from both populations for 1000 independent replicates of *J *=* *20 loci, yielding 1000 independent draws of the true *F*_2_ statistic, which ranged across the set of values $F_{2}\in [0.02,0.12]$. Using these allele frequencies, along with the sample size and relatedness information, we also calculate the difference in MSE between the ${\hat{F}}_{2}$ and ${\tilde{F}}_{2}$ estimators by using Propositions 3, 20, and 21 and the difference in MSE between the $\hat{F}$ and $\overset{⌣}{F}$ estimators by using Corollary 5 and Proposition 22. We calculate the MSE by summing the variance and squared bias. We note that the MSEs of the unbiased estimators are equal to their variances. We repeat this process for $F_{3}(A;B,C),\;F_{3}(A;B,C|A)$, and $F_{4}(A,B;C,D|A)$ as these are the estimators that are biased in their $\hat{F}$ forms.

We use Propositions 6, 23, 25, and 26 and Corollary 8 to determine the MSE for all three $F_{3}$ estimators by including allele frequency information from the JPT (Japanese) population where A=JPT, B=CEU, and C=YRI, with true range for $F_{3}\in [0.00,0.08]$. For the normalized $F_{3}(A;B,C|A)$ estimators, we compare MSE between the biased and unbiased versions by using bias and variances derived in Propositions 9, 28, 31, and 32 and Corollary 11 with true range for normalized $F_{3}\in [0.00,0.30]$. Finally, we estimate the MSE for the normalized $F_{4}(A,B;C,D|A)$ estimators by including GIH (Gujurati Indian) allele frequency data and using the derivations in Propositions 13, 34, 37, and 38 and Corollary 15. In this case, we set A=YRI, B=CEU, C=JPT, and D=GIH for a true range of normalized $F_{4}\in [-0.3,0.2]$.

For each analysis, we estimate MSE for instances when samples of 60 diploid individuals from each population include 30 relative pairs, including 10 avuncular relationships, 10 inbred full siblings, and 10 outbred full siblings. We also assumed every individual was related to exactly one other individual. In these estimates, all populations contain the same composition of related individuals.

**Figure 2:**
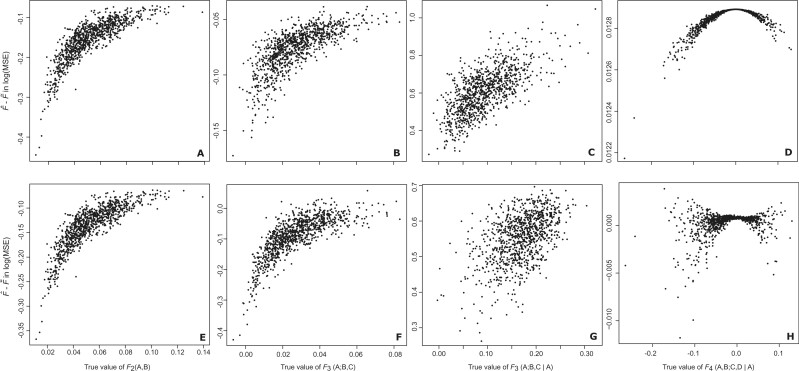
Difference in theoretically calculated ${\;\text{log}\;}_{10}(\text{MSE})$ of $\hat{F}$ and $\tilde{F}$ (A–D) and $\hat{F}$ and $\overset{⌣}{F}$ (E–H) estimators when including relatives or inbred individuals. The MSE is estimated for instances when samples of 60 individuals include individuals related to exactly one other in the sample, with 10 pairs of avuncular relationships, 10 pairs of inbred full siblings and 10 pairs of outbred full siblings. Each point represents calculations from *J *=* *20 randomly sampled loci from the 1000 Genomes Project dataset for CEU, European, YRI African, JPT Japanese, and GIH Indian populations. For $F_{2}(A,B)$ we use A=CEU and B=YRI, while for $F_{3}(A;B,C)$ and $F_{3}(A;B,C|A)$ we use A=JPT, B=CEU, and C=YRI and for $F_{4}(A,B;C,D|A)$ we assign A=YRI, B=CEU, C=JPT, and D=GIH.

The difference in ${\;\text{log}\;}_{10}(\text{MSE})$ between $\hat{F}$ and $\tilde{F}$ estimators for $F_{2}(A,B),\;F_{3}(A;B,C),\;F_{3}(A;B,C|A)$, and $F_{4}(A,B;C,D|A)$ show similar trends with respect to the true *F*-statistic values. Similar trends emerge when examining the difference between $\hat{F}$ and $\overset{⌣}{F}$ estimators. Specifically, the difference in ${\;\text{log}\;}_{10}(\text{MSE})$ decreases as the true *F*-statistic value approaches zero (Figure 2). In our evaluation of $F_{4}(A,B;C,D|A)$, we considered both positive and negative values for its true value, which shows that the difference in ${\;\text{log}\;}_{10}(\text{MSE})$ of ${\hat{F}}_{4}$ and ${\tilde{F}}_{4}$ exhibits a quadratic shaped trend as a function of true *F*_4_. Overall, we notice that the difference in MSE between biased and unbiased estimators is dependent on the true value of the *F*-statistic, with the least difference occurring when the true *F*-statistic is closest to zero.

### Effect of sample size on mean squared error

To probe how sample size within each population affects the difference in estimator error rate, we theoretically computed the MSE for both $\hat{F}$ and $\tilde{F}$ estimators when different numbers of individuals are sampled, with the constraint that every sampled individual is related to exactly one other individual in the sample from that population. Specifically, we evaluate the impact on these estimators when sampling from one pair to 50 pairs of related individuals, with relationships of inbred full sibling pairs ($\Phi_{xy}=3/8$), outbred full sibling pairs ($\Phi_{xy}=1/4$), parent-offspring pairs ($\Phi_{xy}=1/4$), and avuncular pairs ($\Phi_{xy}=1/8$). We compute the MSE as in *Effect of population F-statistic value on* *MSE* section above.

**Figure 3:**
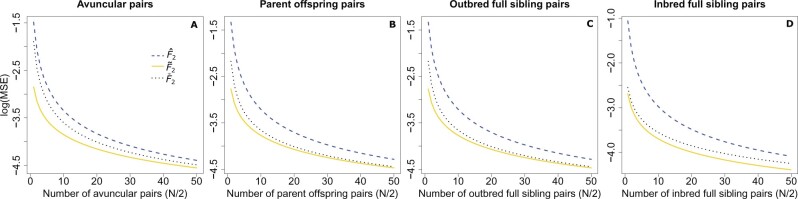
Mean squared error theoretically calculated for ${\hat{F}}_{2}(A,B)$, ${\overset{⌣}{F}}_{2}(A,B)$, and ${\tilde{F}}_{2}(A,B)$ across different sample sizes or related pairs of individuals, including avuncular relationships (A), parent-offspring relationships (B), outbred full siblings (C), and inbred full siblings (D). The number of sampled individuals ranges from 2 to 100 with the number of relative pairs equaling half the total sampled, all computed using *J *=* *20 loci. The true value of $F_{2}(A,B)$ is 0.071.

In almost all cases, the biased $\hat{F}$ and the unbiased $\overset{⌣}{F}$ estimators always displayed elevated MSE compared to their corresponding unbiased $\tilde{F}$ estimators (Figure 3 and Supplementary Figures S1–S3), with the $\overset{⌣}{F}$ estimators always having values between the $\hat{F}$ and $\tilde{F}$ estimators, and usually being closer to $\tilde{F}$ than to $\hat{F}$. For all estimators, we see a clear decrease in the MSE as the number of sampled individuals increases, with the greatest error observed when two individuals are sampled. As expected, a greater sample size allows one to better estimate allele frequencies, and ultimately reduces the mean pairwise kinship coefficient within the sample, as the number of pairs in the sample grows quadratically but the number of relative pairs grows linearly. We also find that the difference in the MSE at larger sample sizes is not as pronounced for normalized *F*_4_ as it is for *F*_2_, *F*_3_, and normalized *F*_3_, as the difference in bias among the biased and unbiased estimators is much smaller for normalized *F*_4_ (Supplementary Figure S3).

### Effect of sample composition on mean squared error

Different types of relatives have different proportions of their alleles shared identical by descent, and thus have different pairwise kinship coefficients. Because we have demonstrated that bias and variance (and hence MSE) of estimators are influenced by within-population mean pairwise kinship coefficient across sampled individuals, the distribution of relative types within a sample will impact overall *F*-statistic estimation error. For this reason, it is important to examine how our *F*-statistics are affected by samples containing diverse mixtures of relative types. Specifically, to accurately assess the impact of relative composition, we hold sample sizes, number of relative pairs, and true population *F*-statistic values constant.

**Figure 4:**
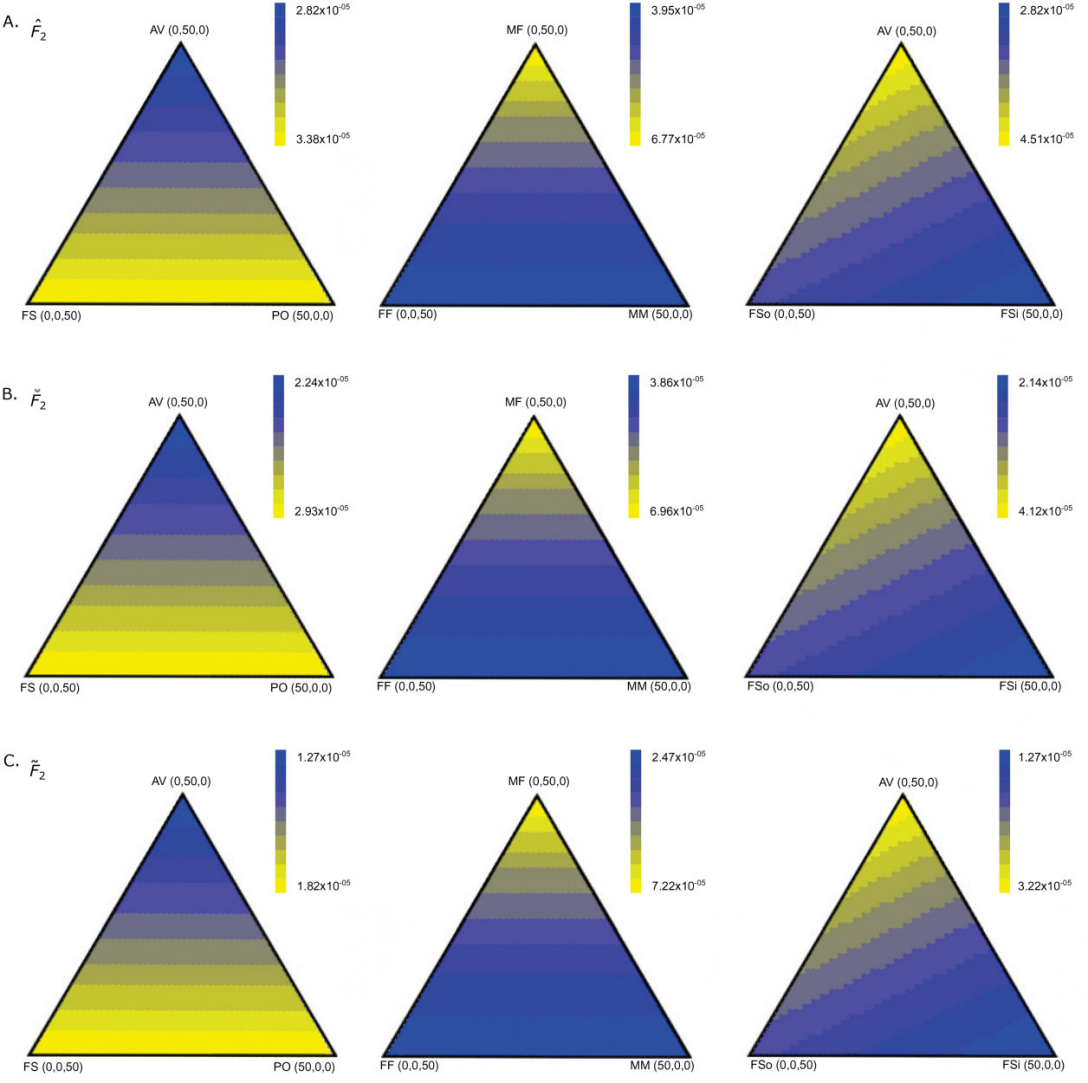
Theoretically calculated MSE of ${\hat{F}}_{2}(A,B)$ (A), ${\overset{⌣}{F}}_{2}(A,B)$ (B), and ${\tilde{F}}_{2}(A,B)$ (C) when including relatives or inbred individuals for *J *=* *20 loci. The MSE is estimated for instances when samples of 100 individuals include individuals related to exactly one other in the sample. The first column shows MSE for samples with different combinations of parent-offspring (PO), full sibling (FS), and avuncular (AV) relationships, the second includes full siblings that are male–male (MM), male–female (MF), and female–female (MF). The last column includes AV relationships as well as inbred (FSi) and outbred (FSo) full siblings. The true value of $F_{2}(A,B)$ is 0.071.

We computed the theoretical MSE when samples of 50 pairs of relatives (100 diploid individuals sampled) contain relative pairs of three different types as in Harris and DeGiorgio (2017a). In addition, each individual is related to exactly one other individual in the sample from the same population. For each statistic we vary the number of pairs related by each of three types of relationships between zero and 50, with 1326 combinations for each. We repeat this process for three configurations of relationships to probe estimator error as a function of the mixture of relative types. We also provide comparisons among inclusion of male–male full siblings ($\Phi_{xy}=1/2$), male–female full siblings ($\Phi_{xy}=3/8$) and female–female full siblings ($\Phi_{xy}=1/4$) at mixed-ploidy loci such as on the X chromosome (DeGiorgio *et al.* 2010), with results showing elevated MSE for both estimators for higher male–male sibling proportions, when compared to male–female or female–female full siblings. To investigate the effects of inbred individuals, we also provide a comparison between inbred full siblings ($\Phi_{xy}=3/8$) with inbreeding coefficient $f_{x}=f_{y}=1/4$, and outbred full-siblings ($\Phi_{xy}=1/4$) at a autosomal diploid loci. We see that MSE is higher for inbred full-siblings than for outbred full-siblings in all cases examined (Figure 4 and Supplementary Figures S4–S6).

We also note that the value of MSE for the biased $\hat{F}$ estimators is always greater than the value for their corresponding unbiased $\tilde{F}$ statistics, which is true in part due to the values of the true *F*-statistics for the loci we chose to use. In addition, the values for the $\overset{⌣}{F}$ estimators are also higher than the corresponding $\tilde{F}$ estimators, but are lower than respective $\hat{F}$ estimators for all combinations of individuals. Though the MSE is higher for the biased estimators, the variation in MSE values is similar for all estimators. For example, the data point with the highest proportion of avuncular relatives has the lowest MSE when compared to parent-offspring relationships and outbred full siblings. In all tested settings (Figure 4 and Supplementary Figures S4–S6), we notice similar patterns of MSE variation when comparing $\hat{F}$ estimators with $\overset{⌣}{F}$ and $\tilde{F}$ estimators. This pattern is again shared when comparing MSE variation among the estimators for *F*_2_, *F*_3_, normalized *F*_3_, and normalized *F*_4_. We can conclude that in all of these cases, the value of the mean kinship coefficient is most important in determining MSE when sample size and true *F*-statistic value are fixed.

### Simulations to evaluate theoretical MSE approximations

To verify that our theoretical approximations for MSE are reasonable, we simulate samples containing related individuals and use them to compute the biased $\hat{F}$- and unbiased $\tilde{F}$-statistics as well as calculate their biases, variances, and MSEs. For each population (CEU, YRI, GIH, and JPT), we simulate 10 noninbred parent-offspring pairs with each individual related to exactly one other individual in the sample. Genotypes for each individual are simulated by first sampling two alleles with replacement according to their respective population allele frequencies from each of the populations (CEU, YRI, GIH, and JPT) to create a set of 20 unrelated individuals per population. Individuals *x* and *y* that form one of the 10 relative pairs have the genotype of individual *y* modified according to their relationship type. Specifically, for each relative type, there are probabilities $\Delta_{0},\;\Delta_{1}$, and $\Delta_{2}$ that the two individuals will share zero, one, or two alleles identical by descent, respectively. The first allele of individual *y* is copied from the first allele from individual *x* with probability $\Delta_{1}$ and the entire genotype of individual *x* is copied over to individual *y* with probability $\Delta_{2}$. This process is repeated across 20 independent loci to generate a sample of 20 individuals with 10 relative pairs in each population with genotypes taken at *J *=* *20 independent loci. To generate 20 independent loci from the four 1000 Genomes Project populations, we used loci either on separate chromosomes, or at least one megabase away from each other.

**Figure 5:**
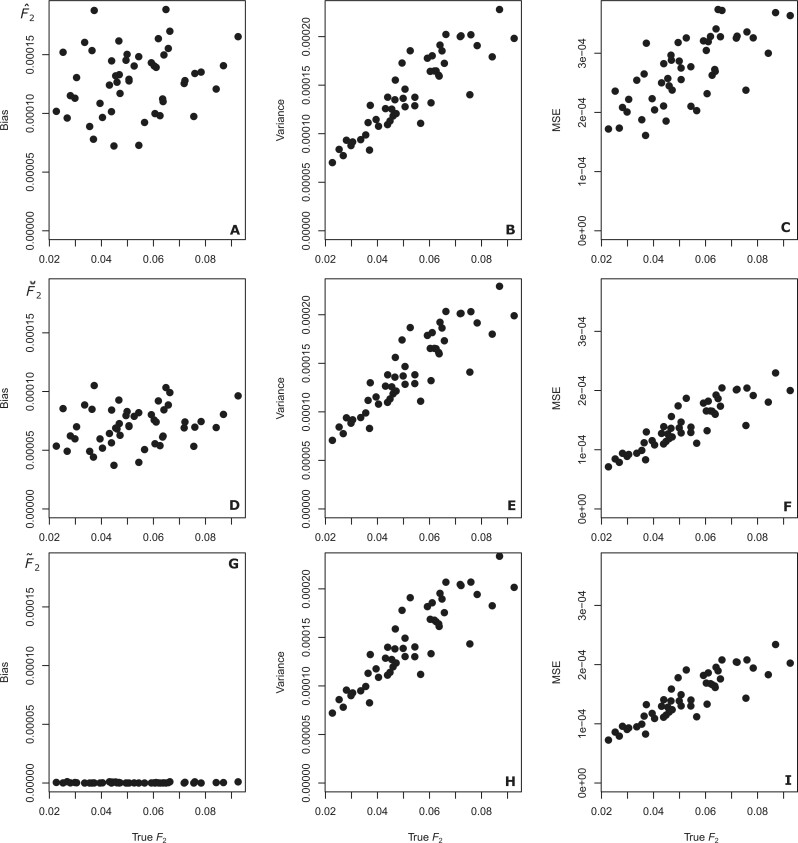
Comparison of squared bias (A, D, and G), variance (B, E, and H), and MSE (C, F, and I), for ${\hat{F}}_{2}(A,B)$, ${\overset{⌣}{F}}_{2}(A,B)$, and ${\tilde{F}}_{2}(A,B)$ from simulated data including 60 parent offspring relative pairs. Each estimate was computed from *J *=* *20 randomly sampled loci using A=CEU and B=YRI.

For each of our new unbiased estimators we compute the bias, variance, and MSE along with the same values for the original estimators (Figure 5 and Supplementary Figures S7–S9). Comparing the bias measurements in these figures, we observe a clear reduction in bias when applying the $\tilde{F}$ and $\overset{⌣}{F}$ estimators as opposed to the $\hat{F}$ estimators. Importantly, the bias measurements for the $\overset{⌣}{F}$ estimators are always higher than the bias measures for $\tilde{F}$ estimates, as the $\overset{⌣}{F}$ estimators do not account for relatives. However, the variances are highly similar for $\hat{F}$, $\overset{⌣}{F}$, and $\tilde{F}$ in all cases. As the value of variance is much larger than the magnitude of the bias (by an order of magnitude) and hence the squared bias, the resulting MSE is consequently similar as well. Because *F*_4_ is quantifying the relationship among four populations, more simulations may be required to converge to the pattern seen by theoretical simulations. For this reason, we increased the number of simulations used to compute the bias, variance, and MSE to 10^4^ for each data point in Supplementary Figure S9, whereas 10^3^ simulation replicates were used for *F*_2_, and both versions of *F*_3_.

To compare the accuracy of our theoretical approximations to simulation results across a spectrum of relatedness between individuals in a sample, we simulate combinations of parent offspring, outbred full sibling, and avuncular relationships. In a manner similar to described above (first paragraph of *Simulations to evaluate theoretical MSE approximations*), we simulate a total of 10 relative pairs made up of a combination of each of the three relative types, with the number of each relative type ranging from 0 to 10. We simulate each of these 66 distinct settings of relative type combinations with genotypes sampled at *J *=* *20 independent loci, and completed 1000 independent replicates of each setting to obtain accurate measurements of bias, variance, and MSE for each simulation setting, with each simulation using true *F*-statistic values specified in (Figure 5 and Supplementary Figures S7–S9). We compute the bias, variance, and MSE for simulations, and compare these values to theoretically calculated computations for each relative combination (Supplementary Figures S11–S14). We find that although noisier, the bias, variance, and MSE patterns in our simulation results match theoretical calculations, suggesting that our theoretical computations are accurate. For all cases, the simulated bias measurements for the $\tilde{F}$ estimators are close to zero, whereas the $\hat{F}$ estimators display bias measurements matching the theoretically calculated $\hat{F}$ bias values.

### Utility and applications of unbiased estimators

In previous sections, we have shown through simulations that our theoretical results produced expected patterns and evaluated the performance of our unbiased estimators under varying combinations of relatives, true *F*-statistic values, and sample sizes. In this section, we show some potential applications of these estimators, using both simulated and empirical data. As discussed previously in the *Introduction*, the value of $F_{3}(A;B,C)$ can be used to identify whether population *A* is the result of admixture between populations related to *B* and *C* (Figure 1). A negative value of *F*_3_ indicates the presence of this process, whereas a nonnegative value is inconclusive and means that further tests may be required to verify a history of admixture. However, because ${\hat{F}}_{3}$ is upwardly biased and because ${\tilde{F}}_{3}$ corrects for this bias, ${\tilde{F}}_{3}$ might allow us to detect admixture in cases where ${\hat{F}}_{3}$ would be inconclusive, even without the presence of related or inbred individuals.

To explore this hypothesis, we first examine an admixture scenario in which $F_{3}(A;B,C)$ might provide marginally negative values. We simulate two populations (*B* and *C*) with effective population size of 10^4^ diploid individuals (Takahata 1993) that diverged 2000 generations prior to sampling using SLiM (Haller and Messer, 2019). This simple divergence model has parameters inspired by the history relating African and non-African human populations (Gravel *et al.* 2011). These populations then merge with admixture proportions 0.4 and 0.6 for *B* and *C*, respectively, to form population *A* 400 generations prior to sampling. Using these parameters, the expected value is $F_{3}(A;B,C)=-0.0568$. To generate genetic data from this model, we evolved sequences with a per-site per-generation mutation rate of $\mu =1.25\times 10^{-8}$ (Scally and Durbin 2012) and a uniform per-site per-generation recombination rate of $r=10^{-8}$ (Payseur and Nachman 2000). We output 20 two megabase chromosomal regions containing allele frequency information for all three populations. Using allele frequency information from the three populations (*A, B*, and *C*) we generate 50 individuals for each population, in which there are 25 parent-offspring pairs. We then compute ${\tilde{F}}_{3}(A;B,C)$, ${\overset{⌣}{F}}_{3}(A;B,C)$, and ${\hat{F}}_{3}(A;B,C)$ across *J *=* *20 loci, either on separate chromosomes or at least one megabase away from each other to ensure independence.

**Figure 6:**
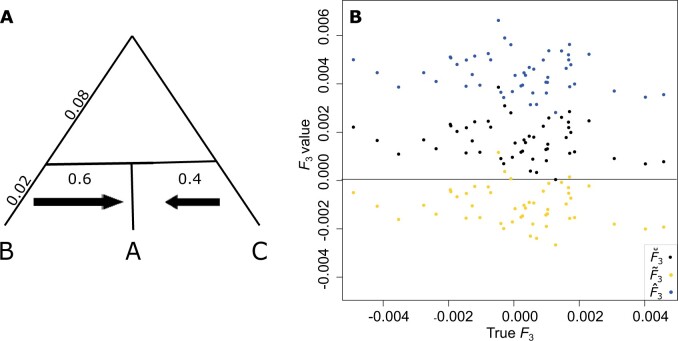
${\hat{F}}_{3}(A;B,C)$ , ${\overset{⌣}{F}}_{3}(A;B,C)$, and ${\tilde{F}}_{3}(A;B,C)$ calculated for simulations where populations *B* and *C* merge with admixture proportions of 0.4 and 0.6, respectively, 400 generations ago (0.02 coalescent units) to form population *A* (tree shown in panel A). Panel (B) shows results for a sample containing 25 parent-offspring pairs.

Figure 6 illustrates that ${\tilde{F}}_{3}$ values are lower than ${\hat{F}}_{3}$, with ${\overset{⌣}{F}}_{3}$ values falling in between ${\hat{F}}_{3}$ and ${\tilde{F}}_{3}$. ${\tilde{F}}_{3}$ values are almost always negative while ${\hat{F}}_{3}$ and ${\overset{⌣}{F}}_{3}$ values are almost always positive. Because this statistic is used to test for admixture and a negative result indicates the presence of admixture, the use of biased estimators when related individuals are included in the sample leads to a different conclusion than when using the unbiased estimator. However, we notice that there is almost no correlation between the true *F*_3_ values and the estimates of *F*_3_ in Figure 6. This lack of correlation is due to the fact that the *F*_3_ values we simulated for this experiment are drawn from a particularly small range, and correlations in estimated versus true *F*_3_ are unable to be observed over the estimation noise. To ensure that this is indeed the case, we conduct simulations across a larger range of true *F*_3_ values and estimate ${\tilde{F}}_{3},\;{\hat{F}}_{3}$, and ${\overset{⌣}{F}}_{3}$. We notice that the expected trend appears when the range of *F*_3_ values is expanded (Supplementary Figure S15).

**Figure 7:**
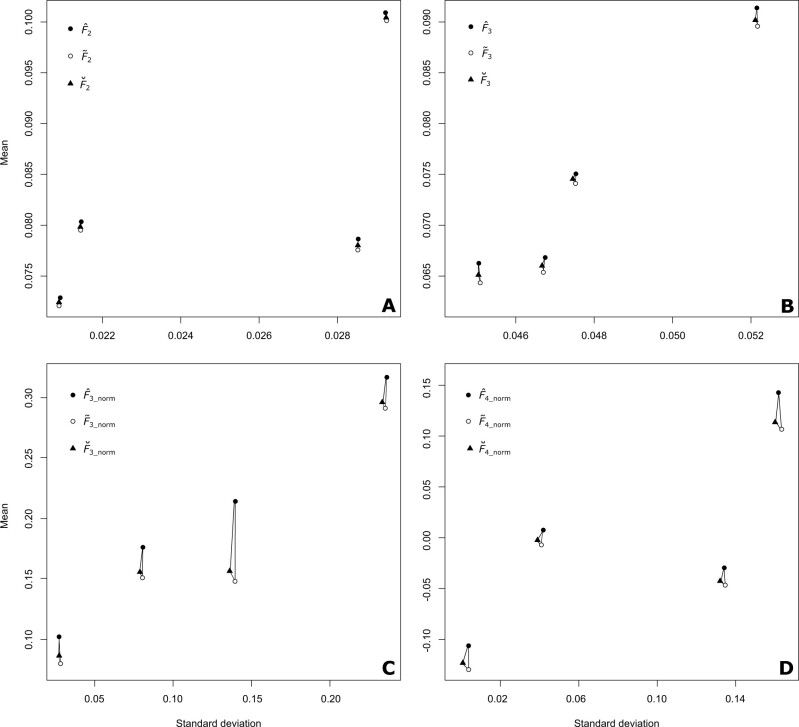
The difference between the means and standard deviations of biased, unbiased Patterson’s, and our new unbiased estimators of *F*_2_, normalized and un-normalized *F*_3_ and normalized *F*_4_ when estimated with genotype information from four different combinations of Colombian, Lahu, Melanesian, Mandenka, San, and Druze populations. All of these populations include between 2 and 14 relative pairs. The black dots represent the values for the biased estimators, while the white dots show the value for the unbiased estimators. Each mean and standard deviation was calculated for a combination of two, three, or four populations, for *F*_2_, *F*_3_, and *F*_4_, respectively, and consists of 1000 estimates of the statistic, each calculated from *J *=* *20 randomly samples single nucleotide polymorphisms from the genome. Panel (A) has values for $F_{2}(A,B)$, panel (B) has values for $F_{3}(A;B,C)$, panel (C) shows results for normalized $F_{3}(A;B,C|A)$, and panel (D) has results for normalized $F_{4}(A,B;C,D|A)$.

Finally, we test the performance of our statistics on empirical data. We use populations from the HGDP SNP dataset (Li *et al.* 2008) that include related individuals (Rosenberg 2006). Specifically, we use genotype information from Colombian, Lahu, Melanesian, Mandenka, San, and Druze populations, and we sample 20 independent loci that are at least one megabase apart from all populations for 1000 independent replicates of *J *=* *20 loci, yielding 1000 independent draws. Each of these populations contains between two and 14 pairs of inferred related individuals, according to Rosenberg (2006). Using distinct pairs for *F*_2_, triples for *F*_3_, and quadruples for *F*_4_ of these populations and the relationships from Rosenberg (2006), we estimate ${\hat{F}}_{2}(A,B),\;{\tilde{F}}_{2}(A,B)$, ${\overset{⌣}{F}}_{2}(A,B)$, ${\hat{F}}_{3}(A;B,C|A),\;{\tilde{F}}_{3}(A;B,C|A)$, ${\overset{⌣}{F}}_{3}(A;B,C|A)$, ${\hat{F}}_{4}(A,B;C,D|A),\;{\tilde{F}}_{4}(A,B;C,D|A)$, and ${\overset{⌣}{F}}_{4}(A,B;C,D|A)$, and compare the mean and standard deviation of the biased and unbiased estimators (Figure 7). In all cases shown, the biased estimator has higher mean than the unbiased estimator, although the standard deviations are similar for both. This indicates that correcting the bias generated by related individuals yields more accurate *F*-statistic estimates with minimal cost in precision of the estimates. In addition, the unbiased $\overset{⌣}{F}$ estimators presented in Appendix A of Patterson *et al.* (2012) all have lower means than the biased $\hat{F}$ estimators, and are more similar to our unbiased $\tilde{F}$ estimators, while having standard deviation measures that are a lower than both other estimators. This could indicate that Patterson’s unbiased estimators have slightly higher precision, while having slightly lower accuracy than the unbiased estimators introduced in this article when samples contain related individuals.

## Discussion

We have introduced the unbiased estimators ${\tilde{F}}_{2}(A,B),\;{\tilde{F}}_{3}(A;B,C),\;{\tilde{F}}_{3}(A;B,C|A)$, and ${\tilde{F}}_{4}(A,B;C,D|P)$ as well as shown that the estimators ${\hat{F}}_{4}(A,B;C,D)$ and $\hat{D}(A,B,C,D)$ are unbiased with the inclusion of related and inbred individuals. In addition, we have demonstrated that the variance of ${\tilde{F}}_{2}(A,B)$ is similar to that of ${\hat{F}}_{2}(A,B)$, as are the variances of ${\tilde{F}}_{3}(A;B,C)$ and ${\hat{F}}_{3}(A;B,C)$. We have also provided variance calculations for all other *F*- and *D*-statistic estimators included in this study. Using these calculations, we have compared the performance of the biased and newly derived unbiased estimators, and shown that in most cases the unbiased estimators have lower MSE values than the biased estimators of the same statistic.

Interestingly, the two statistics that sample from each analyzed population only once per locus—${\hat{F}}_{4}(A,B;C,D)$ and $\hat{D}(A,B,C,D)$—are unbiased with the inclusion of related or inbred individuals, whereas ${\hat{F}}_{2}(A,B)$, which samples from each population *A* and *B* twice, and ${\hat{F}}_{3}(A;B,C)$, which samples from population *A* twice, are biased. This process of sampling more than once from a single population per locus is responsible for creating bias due the inclusion of related or inbred individuals within the twice-sampled population.

The development of these unbiased statistics, and the proofs showing other statistics are unbiased is beneficial for anthropologists interested in populations such as hunter-gatherers, some of which are often small and widely dispersed yet retain high genetic diversity (Kim *et al.* 2014). Small population sizes may necessitate the sampling of close relatives, such as parents and offspring, or siblings. Along with small human populations, these statistics are often applied to nonhuman species. Some, such as elephants, rhinoceros, and cheetahs are close to extinction or have extremely small and inbred populations due to human activity. The *F*- and *D*-statistics may prove important in conservation efforts to test how (and whether) different populations of these animals are interacting. For these reasons, having estimators that are unbiased under such conditions is imperative in making accurate inferences about the relationships of such small populations with others. Although it may not be possible to identify relatives through the sampling process, especially in the case of wild animals, there are methods available to identify related individuals and estimate their likely degree of relatedness once the samples have been sequenced (Epstein *et al.* 2000). The inferences from these methods will allow users to identify pairwise kinship coefficients necessary to apply the unbiased statistics of this study.

A key consideration when evaluating the importance unbiased estimators of *F*- and *D*-statistics is their potential use. Specifically, a number of applications of these statistics do not employ the raw estimates, but instead standardized estimates (Soraggi *et al.* 2018; Zheng and Janke 2018), where a particular *F*- or *D*-statistic has its genomewide mean subtracted, and is normalized by the standard error using a genomic block jackknife procedure (Reich *et al.* 2009). Indeed, subtracting out this genomewide mean may circumvent bias issues. However, this assumes that all genomic blocks have similar sample properties, yet blocks with reduced sample size (*e.g.*, in regions with difficult to call genotypes) may still deviate from the genomewide expectation. In contrast, accounting for this bias due to relatedness would provide estimates closer to the genomewide mean. Because the variance for these biased and unbiased estimators is approximately the same (compare Propositions 20 vs 21 and 23 vs 25), the standard errors used for normalizing these statistics are expected to be comparable, and thus, the unbiased estimators of the *F*-statistics derived here represent a more robust alternative to the original biased estimators, regardless of whether the raw or standardized values of the statistics are used. Furthermore, the raw value of some statistics, such as using the *F*_3_ statistic to detect population admixture, is important, and without correcting the bias of such statistics (Figure 6), key historical events relating populations could be missed. In addition, applying these statistics to genomic regions that are likely to be evolving non-neutrally (*e.g.*, protein-coding regions) may lead to skewed estimates due to selection. For this reason, it is recommended that these statistics be applied to intergeneic regions.

The *F*- and *D*-statistics evaluated here are the most commonly used. However, since their development by Reich *et al.* (2009) and Patterson *et al.* (2012), other *D*-statistic type tests have been formulated to not only detect admixture, but also to identify the direction of gene flow—namely the partitioned *D*-statistics of Eaton and Ree (2013) and the $D_{\text{FOIL}}$ statistics of Pease and Hahn (2015). Specifically, the $D_{\text{FOIL}}$ statistics as originally formulated by Pease and Hahn (2015) sampled a single lineage (or allele) from each of a set of five populations *A*, *B*, *C*, *D*, and *O*, with a symmetric rooted topology ((AB)(CD)) relating populations *A*, *B*, *C*, and *D*, and with *O* an outgroup to these populations used to polarize the ancestral allelic state. Subsequently, Harris and DeGiorgio (2017b) derived allele frequency formulas for the $D_{\text{FOIL}}$ statistics, and showed that allele frequency information for the outgroup population *O* is not needed for computation. The $D_{\text{FOIL}}$ statistics are a set of four quantities (Harris and DeGiorgio 2017b)

(68) $$D_{\text{FO}}(A,B;C,D)=\frac{\sum_{j=1}^{J}\left(1-2a_{j}\right)(d_{j}-c_{j})}{\sum_{j=1}^{J}\left(c_{j}+d_{j}-2c_{j}d_{j}\right)}$$

 

(69) $$D_{\text{IL}}(A,B;C,D)=\frac{\sum_{j=1}^{J}\left(1-2b_{j}\right)(d_{j}-c_{j})}{\sum_{j=1}^{J}\left(c_{j}+d_{j}-2c_{j}d_{j}\right)}$$

 

(70) $$D_{\text{FI}}(A,B;C,D)=\frac{\sum_{j=1}^{J}\left(1-2c_{j}\right)(b_{j}-a_{j})}{\sum_{j=1}^{J}\left(a_{j}+b_{j}-2a_{j}b_{j}\right)}$$

 

(71) $$D_{\text{OL}}(A,B;C,D)=\frac{\sum_{j=1}^{J}\left(1-2d_{j}\right)(b_{j}-a_{j})}{\sum_{j=1}^{J}\left(a_{j}+b_{j}-2a_{j}b_{j}\right)},$$

each of which does not have the frequencies for two alleles sampled from a single population multiplying each other. Hence, using sample allele frequencies in place of the population quantities would still yield approximately unbiased estimators of the $D_{\text{FOIL}}$ statistics, regardless of whether related or inbred individuals were included in the sample. Though we chose to focus on the more classic *F*- and *D*-statistics, variance quantities for these partitioned *D* and $D_{\text{FOIL}}$ statistics can be readily computed as we have done for other ratio estimators in this study.

Though we have only shown results when all populations contain samples with the same relative pair composition, it is trivial to include different relative types in different populations within these statistics. In addition, a key assumption of our theoretical formulas is that pairwise relative contributions to the bias and variance are so much larger in magnitude than higher-order relative contributions, that the inclusion of kinship terms for trios, quadruples, or pairs of pairs of relatives would minimally affect results. For this reason, and to avoid highly unwieldy formulas for variance calculations, we made approximations to the variance formulas using this assumption. We briefly explore the accuracy of such approximations under simulations with 20 full sibling trios, and calculate the bias, variance, and MSE over a range of *F*_2_ values. We compare these simulated results to theoretically calculated bias, variance, and MSE for ${\tilde{F}}_{2}$ across identical *F*_2_ values (Supplementary Figure S16), where the theoretical variance (and hence MSE) formulas are approximations that only consider pairwise kinship coefficients. We see that though the trends in variance (and hence MSE) values are similar between the simulated and theoretical quantities, there is a slight difference between the simulated and theoretical variance (and hence MSE), where the theoretical values are consistently lower than the simulated. Therefore, our theoretical approximate variance calculations have underestimated the true variance values, which can be expected as we drop a number of terms that would be in the exact variance calculation, while still being useful for understanding the overall trends of the variances (and hence MSEs) across estimators.

In addition, it is also possible to apply our new unbiased estimators when only some or none of the populations contain related or inbred individuals. Moreover, though we have demonstrated results for allele frequencies estimated as the sample proportion, we could have instead used the best linear unbiased estimator (BLUE) of McPeek *et al.* (2004), as all derivations in this article are based on a general form of a linear unbiased estimator. The BLUE allele frequency estimator would have superior properties to the sample proportion discussed here, as it has smallest variance (McPeek *et al.* 2004), and this reduction in variance translates to functions of the allele frequency as highlighted by improvements in both expected heterozygosity and $F_{\text{ST}}$ by Harris and DeGiorgio (2017a). To apply the BLUE estimator, we would simply alter the weight $\phi_{x}(P)$ of an individual *x* in population *P* at a particular locus with the equation

(72) $$\phi_{x}(P)=\frac{\sum_{k=1}^{N(P)}{\left(K^{-1}\right)}_{kx}}{1^{T}K^{-1}1},$$

where $K\in R^{N(P)\times N(P)}$ is the matrix of pairwise kinship coefficients, with element in row *j* and column *k* given by $K_{jk}=\Phi_{jk},\;1\in R^{N(P)}$ is a column vector of ones, and superscript T indicates transpose. To facilitate easy application of these statistics, we have developed open-source software funbiased for use by the scientific community, which is available at https://github.com/MehreenRuhi/funbiased.

## Data availability

Supplementary data are available at Genetics online. The 1000 Genomes Project data used in this publication is available at http://www.1000genomes.org/. Relatedness information used to generate Figure 7 is available online within Supplementary Tables S7–S15 of Rosenberg (2006).

## Supplementary Material

iyab090_Supplementary_Figures

## Appendix

In this section, we provide proofs of key lemmas and propositions from the *Theory* section, and also develop and prove other important results.

*Proof of Lemma 1*. We first calculate (result in Weir 1996, page 153)

$$\begin{array}{l} E[\hat{G}(P_{j})]=E[{\hat{p}}_{j}(1-{\hat{p}}_{j})]\\ =E[{\hat{p}}_{j}]-E[{\hat{p}}_{j}^{2}]\\ =p_{j}-[1-\Phi_{2}(P_{j})]p_{j}^{2}-\Phi_{2}(P_{j})p_{j}\\ =[1-\Phi_{2}(P_{j})]p_{j}(1-p_{j})\\ =[1-\Phi_{2}(P_{j})]G(P_{j}), \end{array}$$

which gives

$$\begin{array}{l} E[\hat{G}(P)]=\frac{1}{J}\sum_{j=1}^{J}E[\hat{G}(P_{j})]\\ =\frac{1}{J}\sum_{j=1}^{J}[1-\Phi_{2}(P_{j})]G(P_{j})\\ =G(P)+\Delta (P), \end{array}$$

where we define the downward bias of $\hat{G}(P)$ as

$$\begin{array}{l} \Delta (P)=E[\hat{G}(P)]-G(P)\\ =-\frac{1}{J}\sum_{j=1}^{J}\Phi_{2}(P_{j})G(P_{j}). \end{array}$$

It follows that $\tilde{G}(P)$ is an unbiased estimator of *G*(*P*) because

$$\begin{array}{l} E[\tilde{G}(P)]=\frac{1}{J}\sum_{j=1}^{J}\frac{1}{1-\Phi_{2}(P_{j})}\hat{G}(P_{j})\\ =\frac{1}{J}\sum_{j=1}^{J}\frac{1}{1-\Phi_{2}(P_{j})}[1-\Phi_{2}(P_{j})]G(P_{j})\\ =\frac{1}{J}\sum_{j=1}^{J}\hat{G}(P_{j})\\ =G(P).\;□ \end{array}$$

*Proof of Corollary 2*. When using the sample proportion to estimate allele frequencies, the weight of individual *x* in population *P* at locus *j* is (Harris and DeGiorgio 2017a)

$$\phi_{x}(P_{j})=\frac{m_{x}}{\sum_{k=1}^{N(P_{j})}m_{k}}.$$

Plugging into the definition of $\Phi_{2}(P_{j})$, we have

$$\begin{array}{l} \Phi_{2}(P_{j})=\sum_{w=1}^{N(P_{j})}\sum_{x=1}^{N(P_{j})}\phi_{w}(P_{j})\phi_{x}(P_{j})\Phi_{wx}\\ =\frac{1}{{\left(\sum_{k=1}^{N(P_{j})}m_{k}\right)}^{2}}\sum_{w=1}^{N(P_{j})}\sum_{x=1}^{N(P_{j})}m_{w}m_{x}\Phi_{wx}. \end{array}$$

Because the sample contains unrelated individuals, then $\Phi_{wx}=0$ for w≠x and $\Phi_{xx}=1/m_{x}$ resulting in

$$\begin{array}{l} \Phi_{2}(P_{j})=\frac{1}{{\left(\sum_{k=1}^{N(P_{j})}m_{k}\right)}^{2}}\sum_{w=1}^{N(P_{j})}m_{w}^{2}\frac{1}{m_{w}}\\ =\frac{1}{\sum_{k=1}^{N(P_{j})}m_{k}}. \end{array}$$

Plugging $\Phi_{2}(P_{j})$ into the definitions of $\text{Bias}[\hat{G}(P)]$ and $\tilde{G}(P_{j})$ within Lemma 1 gives the desired result. □

*Proof of Proposition 3*. We first calculate

$$\begin{array}{l} E[{\hat{F}}_{2}(A_{j},B_{j})]=E[{\left({\hat{a}}_{j}-{\hat{b}}_{j}\right)}^{2}]\\ =E[{\hat{a}}_{j}^{2}]+E[{\hat{b}}_{j}^{2}]-2E[{\hat{a}}_{j}]E[{\hat{b}}_{j}]\\ =[1-\Phi_{2}(A_{j})]a_{j}^{2}+\Phi_{2}(A_{j})a_{j}+[1-\Phi_{2}(B_{j})]b_{j}^{2}+\Phi_{2}b_{j}-2a_{j}b_{j}\\ ={\left(a_{j}-b_{j}\right)}^{2}+\Phi_{2}(A_{j})a_{j}(1-a_{j})+\Phi_{2}(B_{j})b_{j}(1-b_{j})\\ =F_{2}(A_{j},B_{j})+\Phi_{2}(A_{j})G(A_{j})+\Phi_{2}(B_{j})G(B_{j}), \end{array}$$

which gives

$$\begin{array}{l} E[{\hat{F}}_{2}(A,B)]=\frac{1}{J}\sum_{j=1}^{J}E[{\hat{F}}_{2}(A_{j},B_{j})]\\ =\frac{1}{J}\sum_{j=1}^{J}[F_{2}(A_{j},B_{j})+\Phi_{2}(A_{j})G(A_{j})+\Phi_{2}(B_{j})G(B_{j})]\\ =F_{2}(A,B)+\Delta (A,B), \end{array}$$

where we define the upward bias of ${\hat{F}}_{2}(A,B)$ as

$$\begin{array}{l} \Delta (A,B)=E[{\hat{F}}_{2}(A,B)]-F_{2}(A,B)\\ =\frac{1}{J}\sum_{j=1}^{J}[\Phi_{2}(A_{j})G(A_{j})+\Phi_{2}(B_{j})G(B_{j})]. \end{array}$$

It follows that ${\tilde{F}}_{2}(A,B)$ is an unbiased estimator of $F_{2}(A,B)$ because

$$\begin{array}{l} E[{\tilde{F}}_{2}(A,B)]=\frac{1}{J}\sum_{j=1}^{J}(E[{\hat{F}}_{2}(A_{j},B_{j})]-\Phi_{2}(A_{j})E[\tilde{G}(A_{j})]-\Phi_{2}(B_{j})E[\tilde{G}(B_{j})])\\ =\frac{1}{J}\sum_{j=1}^{J}[F_{2}(A_{j},B_{j})+\Phi_{2}(A_{j})G(A_{j})+\Phi_{2}(B_{j})G(B_{j})-\Phi_{2}(A_{j})G(A_{j})\\ -\Phi_{2}(B_{j})G(B_{j})]\\ =\frac{1}{J}\sum_{J=1}^{J}F_{2}(A_{j},B_{j})\\ =F_{2}(A,B).\;□ \end{array}$$

*Proof of Corollary 4*. Plugging the definition of $\Phi_{2}(P_{j}),\;P\in \{A,B\}$, derived in the proof of Corollary 2 as well as

$$\overset{⌣}{G}(P_{j})=\frac{\sum_{k=1}^{N(P_{j})}m_{k}}{\sum_{k=1}^{N(P_{j})}m_{k}-1}\hat{G}(P_{j})$$

of Corollary 2 into the definitions of $\text{Bias}[{\hat{F}}_{2}(A,B)]$ and ${\tilde{F}}_{2}(A,B)$ within Proposition 3 gives the desired result. □

*Proof of Corollary 5*. From Corollary 4, we have

$${\overset{⌣}{F}}_{2}(A,B)=\frac{1}{J}\sum_{j=1}^{J}[{\hat{F}}_{2}(A_{j},B_{j})-\frac{1}{\sum_{k=1}^{N(A_{j})}m_{k}-1}\hat{G}(A_{j})-\frac{1}{\sum_{k=1}^{N(B_{j})}m_{k}-1}\hat{G}(B_{j})].$$

Because populations *A* or *B* may have related or inbred individuals within them, from the proofs of Lemma 1 and Proposition 3, we have

$$\begin{array}{l} E[{\hat{F}}_{2}(A_{j},B_{j})]=F_{2}(A_{j},B_{j})+\Phi_{2}(A_{j})G(A_{j})+\Phi_{2}(B_{j})G(B_{j})\\ E[\hat{G}(P_{j})]=[1-\Phi_{2}(P_{j})]G(P_{j}) \end{array}$$

yielding

$$\begin{array}{l} E[{\overset{⌣}{F}}_{2}(A,B)]=\frac{1}{J}\sum_{j=1}^{J}[F_{2}(A_{j},B_{j})+\Phi_{2}(A_{j})G(A_{j})+\Phi_{2}(B_{j})G(B_{j})\\ -\frac{1-\Phi_{2}(A_{j})}{\sum_{k=1}^{N(A_{j})}m_{k}-1}G(A_{j})-\frac{1-\Phi_{2}(B_{j})}{\sum_{k=1}^{N(B_{j})}m_{k}-1}G(B_{j})]\\ =\frac{1}{J}\sum_{j=1}^{J}[F_{2}(A_{j},B_{j})+\frac{\Phi_{2}(A_{j})\sum_{k=1}^{N(A_{j})}m_{k}-1}{\sum_{k=1}^{N(A_{j})}m_{k}-1}G(A_{j})+\frac{\Phi_{2}(B_{j})\sum_{k=1}^{N(B_{j})}m_{k}-1}{\sum_{k=1}^{N(B_{j})}m_{k}-1}G(B_{j})]\\ =F_{2}(A,B)+\frac{1}{J}\sum_{j=1}^{J}[\frac{\Phi_{2}(A_{j})\sum_{k=1}^{N(A_{j})}m_{k}-1}{\sum_{k=1}^{N(A_{j})}m_{k}-1}G(A_{j})+\frac{\Phi_{2}(B_{j})\sum_{k=1}^{N(B_{j})}m_{k}-1}{\sum_{k=1}^{N(B_{j})}m_{k}-1}G(B_{j})]. \end{array}$$

It follows that the bias is

$$\begin{array}{l} \text{Bias}[{\overset{⌣}{F}}_{2}(A,B)]=E[{\tilde{F}}_{2}(A,B)]-F_{2}(A,B)\\ =\frac{1}{J}\sum_{j=1}^{J}[\frac{\Phi_{2}(A_{j})\sum_{k=1}^{N(A_{j})}m_{k}-1}{\sum_{k=1}^{N(A_{j})}m_{k}-1}G(A_{j})+\frac{\Phi_{2}(B_{j})\sum_{k=1}^{N(B_{j})}m_{k}-1}{\sum_{k=1}^{N(B_{j})}m_{k}-1}G(B_{j})], \end{array}$$

and because $\Phi_{2}(P_{j})\geq 1/\sum_{k=1}^{N(P_{j})}m_{k}$, this is an upward bias. □

*Proof of Proposition 6*. We first calculate

$$\begin{array}{l} E[{\hat{F}}_{3}(A_{j};B_{j},C_{j})]=E[({\hat{a}}_{j}-{\hat{b}}_{j})({\hat{a}}_{j}-{\hat{c}}_{j})]\\ =E[{\hat{a}}_{j}^{2}]-E[{\hat{a}}_{j}]E[{\hat{c}}_{j}]-E[{\hat{a}}_{j}]E[{\hat{b}}_{j}]+E[{\hat{b}}_{j}]E[{\hat{c}}_{j}]\\ =[1-\Phi_{2}(A_{j})]a_{j}^{2}+\Phi_{2}(A_{j})a_{j}-a_{j}c_{j}-a_{j}b_{j}+b_{j}c_{j}\\ =(a_{j}^{2}-a_{j}c_{j}-a_{j}b_{j}+b_{j}c_{j})+\Phi_{2}(A_{j})a_{j}(1-a_{j})\\ =F_{3}(A_{j};B_{j},C_{j})+\Phi_{2}(A_{j})G(A_{j}), \end{array}$$

which gives

$$\begin{array}{l} E[{\hat{F}}_{3}(A;B,C)]=\frac{1}{J}\sum_{j=1}^{J}E[{\hat{F}}_{3}(A_{j};B_{j},C_{j})]\\ =\frac{1}{J}\sum_{j=1}^{J}[F_{3}(A;B_{j},C_{j})+\Phi_{2}(A_{j})G(A_{j})]\\ =F_{3}(A;B,C)+\Delta (A;B,C), \end{array}$$

where we define the upward bias of ${\hat{F}}_{3}(A;B,C)$ as

$$\begin{array}{l} \Delta (A;B,C)=E[{\hat{F}}_{3}(A;B,C)]-F_{3}(A;B,C)\\ =\frac{1}{J}\sum_{j=1}^{J}\Phi_{2}(A_{j})G(A_{j}). \end{array}$$

It follows that ${\tilde{F}}_{3}(A;B,C)$ is an unbiased estimator of $F_{3}(A;B,C)$ because

$$\begin{array}{l} E[{\tilde{F}}_{3}(A;B,C)]=\frac{1}{J}\sum_{j=1}^{J}(E[{\hat{F}}_{3}(A_{j};B_{j},C_{j})]-\Phi_{2}(A_{j})E[\tilde{G}(A_{j})])\\ =\frac{1}{J}\sum_{j=1}^{J}[F_{3}(A_{j};B_{j},C_{j})+\Phi_{2}(A_{j})G(A_{j})-\Phi_{2}(A_{j})G(A_{j})]\\ =\frac{1}{J}\sum_{J=1}^{J}F_{3}(A_{j};B_{j},C_{j})\\ =F_{3}(A;B,C).\;□ \end{array}$$

*Proof of Corollary 7*. Plugging the definition of $\Phi_{2}(A_{j})$ derived in the proof of Corollary 2 as well as

$$\overset{⌣}{G}(A_{j})=\frac{\sum_{k=1}^{N(A_{j})}m_{k}}{\sum_{k=1}^{N(A_{j})}m_{k}-1}\hat{G}(A_{j})$$

of Corollary 2 into the definitions of $\text{Bias}[{\hat{F}}_{3}(A;B,C)]$ and ${\tilde{F}}_{3}(A;B,C)$ within Proposition 6 gives the desired result. □

*Proof of Corollary 8*. From Corollary 7, we have

$${\overset{⌣}{F}}_{3}(A;B,C)=\frac{1}{J}\sum_{j=1}^{J}[{\hat{F}}_{3}(A_{j};B_{j},C_{j})-\frac{1}{\sum_{k=1}^{N(A_{j})}m_{k}-1}\hat{G}(A_{j})].$$

Because population *A* may have related or inbred individuals within it, from the proofs of Lemma 1 and Proposition 6, we have

$$\begin{array}{l} E[{\hat{F}}_{3}(A_{j};B_{j},C_{j})]=F_{3}(A_{j};B_{j},C_{j})+\Phi_{2}(A_{j})G(A_{j})\\ E[\hat{G}(A_{j})]=[1-\Phi_{2}(A_{j})]G(A_{j}) \end{array}$$

yielding

$$\begin{array}{l} E[{\overset{⌣}{F}}_{3}(A;B,C)]=\frac{1}{J}\sum_{j=1}^{J}[F_{3}(A_{j};B_{j},C_{j})+\Phi_{2}(A_{j})G(A_{j})-\frac{1-\Phi_{2}(A_{j})}{\sum_{k=1}^{N(A_{j})}m_{k}-1}G(A_{j})]\\ =\frac{1}{J}\sum_{j=1}^{J}[F_{3}(A_{j};B_{j},C_{j})+\frac{\Phi_{2}(A_{j})\sum_{k=1}^{N(A_{j})}m_{k}-1}{\sum_{k=1}^{N(A_{j})}m_{k}-1}G(A_{j})]\\ =F_{3}(A;B,C)+\frac{1}{J}\sum_{j=1}^{J}\frac{\Phi_{2}(A_{j})\sum_{k=1}^{N(A_{j})}m_{k}-1}{\sum_{k=1}^{N(A_{j})}m_{k}-1}G(A_{j}). \end{array}$$

It follows that the bias is

$$\begin{array}{l} \text{Bias}[{\overset{⌣}{F}}_{3}(A;B,C)]=E[{\tilde{F}}_{3}(A;B,C)]-F_{3}(A;B,C)\\ =\frac{1}{J}\sum_{j=1}^{J}\frac{\Phi_{2}(A_{j})\sum_{k=1}^{N(A_{j})}m_{k}-1}{\sum_{k=1}^{N(A_{j})}m_{k}-1}G(A_{j}), \end{array}$$

and because $\Phi_{2}(A_{j})\geq 1/\sum_{k=1}^{N(A_{j})}m_{k}$, this is an upward bias. □

*Proof of Proposition 9*. Assuming that the expectation of ${\hat{F}}_{3}(A;B,C|A)$ is approximately equal to the ratio of expectations of ${\hat{F}}_{3}(A;B,C)$ and $2\hat{G}(A)$, we find that

$$\begin{array}{l} E[{\hat{F}}_{3}(A;B,C|A)]=E[\frac{{\hat{F}}_{3}(A;B,C)}{2\hat{G}(A)}]\\ \approx \frac{E[{\hat{F}}_{3}(A;B,C)]}{2E[\hat{G}(A)]}\\ =\frac{\left(1/J)\sum_{j=1}^{J}[F_{3}(A_{j};B_{j},C_{j})+\Phi_{2}(A_{j})G(A_{j})\right]}{\left(2/J)\sum_{j=1}^{J}[1-\Phi_{2}(A_{j})]G(A_{j}\right)}\\ =\frac{F_{3}(A;B,C)+(1/J)\sum_{j=1}^{J}\Phi_{2}(A_{j})G(A_{j})}{2G(A)-(2/J)\sum_{j=1}^{J}\Phi_{2}(A_{j})G(A_{j})}\\ =\frac{F_{3}(A;B,C)}{2G(A)}+\Delta (A;B,C|A)\\ =F_{3}(A;B,C|A)+\Delta (A;B,C|A), \end{array}$$

where we define the upward bias of ${\hat{F}}_{3}(A;B,C|A)$ as

$$\begin{array}{l} \Delta (A;B,C|A)=E[{\hat{F}}_{3}(A;B,C|A)]-F_{3}(A;B,C|A)\\ =\frac{F_{3}(A;B,C)+(1/J)\sum_{j=1}^{J}\Phi_{2}(A_{j})G(A_{j})}{2G(A)-(2/J)\sum_{j=1}^{J}\Phi_{2}(A_{j})G(A_{j})}-\frac{F_{3}(A;B,C)}{2G(A)}\\ =\frac{\left[F_{3}(A;B,C)+G(A)](1/J)\sum_{j=1}^{J}\Phi_{2}(A_{j})G(A_{j}\right)}{2G(A)[G(A)-(1/J)\sum_{j=1}^{J}\Phi_{2}(A_{j})G(A_{j})]}\\ =\frac{\left(1/J)\sum_{j=1}^{J}\Phi_{2}(A_{j})G(A_{j}\right)}{G(A)-(1/J)\sum_{j=1}^{J}\Phi_{2}(A_{j})G(A_{j})}[F_{3}(A;B,C|A)+\frac{1}{2}]. \end{array}$$

We can also see that ${\tilde{F}}_{3}(A;B,C|A)$ is an approximately unbiased estimator of $F_{3}(A;B,C|A)$ because

$$\begin{array}{l} E[{\tilde{F}}_{3}(A;B,C|A)]=E[\frac{{\tilde{F}}_{3}(A;B,C)}{2\tilde{G}(A)}]\\ \approx \frac{E[{\tilde{F}}_{3}(A;B,C)]}{2E[\tilde{G}(A)]}\\ =\frac{F_{3}(A;B,C)}{2G(A)}\\ =F_{3}(A;B,C|A).\;□ \end{array}$$

*Proof of Corollary 10*. Plugging the definition of $\Phi_{2}(A_{j})$ derived in the proof of Corollary 2 as well as

$$\overset{⌣}{G}(A_{j})=\frac{\sum_{k=1}^{N(A_{j})}m_{k}}{\sum_{k=1}^{N(A_{j})}m_{k}-1}\hat{G}(A_{j})$$

of Corollary 2 into the definitions of $\text{Bias}[{\hat{F}}_{3}(A;B,C|A)]$ and ${\tilde{F}}_{3}(A;B,C|A)$ within Proposition 9 gives the desired result. □

*Proof of Corollary 11*. From Corollaries 2, 7, and 10, we have

$${\overset{⌣}{F}}_{3}(A;B,C|A)=\frac{{\overset{⌣}{F}}_{3}(A;B,C)}{2\overset{⌣}{G}(A)},$$

where

$$\begin{array}{l} \overset{⌣}{G}(A)=\frac{1}{J}\sum_{j=1}^{J}\overset{⌣}{G}(A_{j})=\frac{1}{J}\sum_{j=1}^{J}\frac{\sum_{k=1}^{N(A_{j})}m_{k}}{\sum_{k=1}^{N(A_{j})}m_{k}-1}\hat{G}(A_{j})\\ {\overset{⌣}{F}}_{3}(A;B,C)=\frac{1}{J}\sum_{j=1}^{J}[{\hat{F}}_{3}(A_{j};B_{j},C_{j})-\frac{1}{\sum_{k=1}^{N(A_{j})}m_{k}-1}\hat{G}(A_{j})]. \end{array}$$

Because population *A* may have related or inbred individuals within it, from the proofs of Lemma 1 and Proposition 6, we have

$$\begin{array}{l} E[{\hat{F}}_{3}(A_{j};B_{j},C_{j})]=F_{3}(A_{j};B_{j},C_{j})+\Phi_{2}(A_{j})G(A_{j})\\ E[\hat{G}(A_{j})]=[1-\Phi_{2}(A_{j})]G(A_{j}) \end{array}$$

yielding

$$\begin{array}{l} E[{\overset{⌣}{F}}_{3}(A;B,C|A)]\approx \frac{E[{\overset{⌣}{F}}_{3}(A;B,C)]}{2E[\overset{⌣}{G}(A)]}\\ =\frac{F_{3}(A;B,C)+(1/J)\sum_{j=1}^{J}G(A_{j})[\Phi_{2}(A_{j})\sum_{k=1}^{N(A_{j})}m_{k}-1]/[\sum_{k=1}^{N(A_{j})}m_{k}-1]}{\left(2/J)\sum_{j=1}^{J}\frac{\left[1-\Phi_{2}(A_{j})][\sum_{k=1}^{N(A_{j})}m_{k}\right]}{\sum_{k=1}^{N(A_{j})}m_{k}-1}G(A_{j}\right)}\\ =\frac{F_{3}(A;B,C)+X(A)}{2Y(A)}. \end{array}$$

It follows that the approximate bias is

$$\begin{array}{l} \text{Bias}[{\overset{⌣}{F}}_{3}(A;B,C|A)]=E[{\tilde{F}}_{3}(A;B,C|A)]-F_{3}(A;B,C|A)\\ \approx \frac{1}{2}[\frac{1}{Y(A)}-\frac{1}{G(A)}]F_{3}(A;B,C)+\frac{X(A)}{2Y(A)}, \end{array}$$

and because $\Phi_{2}(A_{j})\geq 1/\sum_{k=1}^{N(A_{j})}m_{k}$, then 0<Y(A)≤G(A) and X(A)≥0 making this is an upward approximate bias. □

*Proof of Proposition 12*. We first calculate

$$\begin{array}{l} E[{\hat{F}}_{4}(A_{j},B_{j};C_{j},D_{j})]=E[({\hat{a}}_{j}-{\hat{b}}_{j})({\hat{c}}_{j}-{\hat{d}}_{j}]\\ =E[{\hat{a}}_{j}-{\hat{b}}_{j}]E[{\hat{c}}_{j}-{\hat{d}}_{j}]\\ =(E[{\hat{a}}_{j}]-E[{\hat{b}}_{j}])(E[{\hat{c}}_{j}]-E[{\hat{d}}_{j}])\\ =(a_{j}-b_{j})(c_{j}-d_{j})\\ =F_{4}(A_{j},B_{j};C_{j},D_{j}). \end{array}$$

We show that ${\hat{F}}_{4}(A,B;C,D)$ is unbiased estimator of $F_{4}(A,B;C,D)$ because

$$\begin{array}{l} E[{\hat{F}}_{4}(A,B;C,D)]=\frac{1}{J}\sum_{j=1}^{J}E[{\hat{F}}_{4}(A_{j},B_{j};C_{j},D_{j})]\\ =\frac{1}{J}\sum_{j=1}^{J}F_{4}(A_{j},B_{j};C_{j},D_{j})\\ =F_{4}(A,B;C,D).\;□ \end{array}$$

*Proof of Proposition 13*. Assuming that the expectation of ${\hat{F}}_{4}(A,B;C,D|P)$ is approximately equal to the ratio of expectations of ${\hat{F}}_{4}(A,B;C,D)$ and $\hat{G}(P)$ for some P∈{A,B,C,D}, we find that

$$\begin{array}{l} E[{\hat{F}}_{4}(A,B;C,D|P)]=E[\frac{{\hat{F}}_{4}(A,B;C,D)}{\hat{G}(P)}]\\ \approx \frac{E[{\hat{F}}_{4}(A,B;C,D)]}{E[\hat{G}(P)]}\\ =\frac{F_{4}(A,B;C,D)}{\left(1/J)\sum_{j=1}^{J}[1-\Phi_{2}(P_{j})]G(P_{j}\right)}\\ =\frac{F_{4}(A,B;C,D)}{G(P)-(1/J)\sum_{j=1}^{J}\Phi_{2}(P_{j})G(P_{j})}\\ =\frac{F_{4}(A,B;C,D)}{G(P)}+\Delta (A,B;C,D|P)\\ =F_{4}(A,B;C,D|P)+\Delta (A,B;C,D|P), \end{array}$$

where we define the approximate upward bias of ${\hat{F}}_{4}(A,B;C,D|P)$ as

$$\begin{array}{l} \Delta (A,B;C,D|P)=E[\hat{F}(A,B;C,D|P)]-F_{4}(A,B;C,D|P)\\ =\frac{F_{4}(A,B;C,D)}{G(P)-(1/J)\sum_{j=1}^{J}\Phi_{2}(P_{j})G(P_{j})}-\frac{F_{4}(A,B;C,D)}{G(P)}\\ =\frac{F_{4}(A,B;C,D)(1/J)\sum_{j=1}^{J}\Phi_{2}(P_{j})G(P_{j})}{G(P)[G(P)-(1/J)\sum_{j=1}^{J}\Phi_{2}(P_{j})G(P_{j})]}\\ =\frac{\left(1/J)\sum_{j=1}^{J}\Phi_{2}(P_{j})G(P_{j}\right)}{G(P)-(1/J)\sum_{j=1}^{J}\Phi_{2}(P_{j})G(P_{j})}F_{4}(A,B;C,D|P). \end{array}$$

We can also see that ${\tilde{F}}_{4}(A,B;C,D|P)$ is an approximately unbiased estimator of $F_{4}(A,B;C,D|P)$ because

$$\begin{array}{l} E[{\tilde{F}}_{4}(A,B;C,D|P)]=E[\frac{{\hat{F}}_{4}(A,B;C,D)}{\tilde{G}(P)}]\\ \approx \frac{E[{\hat{F}}_{4}(A,B;C,D)]}{E[\tilde{G}(P)]}\\ =\frac{F_{4}(A,B;C,D)}{G(P)}\\ =F_{4}(A,B;C,D|P).\;□ \end{array}$$

*Proof of Corollary 14*. Plugging the definition of $\Phi_{2}(P_{j}),\;P\in \{A,B,C,D\}$, derived in the proof of Corollary 2 as well as

$$\overset{⌣}{G}(P_{j})=\frac{\sum_{k=1}^{N(A_{j})}m_{k}}{\sum_{k=1}^{N(A_{j})}m_{k}-1}\hat{G}(P_{j})$$

of Corollary 2 into the definitions of $\text{Bias}[{\hat{F}}_{4}(A,B;C,D|P)]$ and ${\tilde{F}}_{4}(A,B;C,D|P)$ within Proposition 13 gives the desired result. □

*Proof of Corollary 15*. From Corollaries 2 and 14, we have

$${\overset{⌣}{F}}_{4}(A,B;C,D|P)=\frac{{\hat{F}}_{4}(A,B;C,D)}{\overset{⌣}{G}(P)},$$

where

$$\begin{array}{l} \overset{⌣}{G}(P)=\frac{1}{J}\sum_{j=1}^{J}\overset{⌣}{G}(P_{j})=\frac{1}{J}\sum_{j=1}^{J}\frac{\sum_{k=1}^{N(P_{j})}m_{k}}{\sum_{k=1}^{N(P_{j})}m_{k}-1}\hat{G}(P_{j})\\ {\hat{F}}_{4}(A,B;C,D)=\frac{1}{J}\sum_{j=1}^{J}{\hat{F}}_{4}(A_{j},B_{j};C_{j},D_{j}). \end{array}$$

Because population *P* may have related or inbred individuals within it, from the proofs of Lemma 1 and Proposition 12, we have

$$\begin{array}{l} E[{\hat{F}}_{4}(A,B;C,D]=F_{4}(A,B;B,D)\\ E[\hat{G}(P_{j})]=[1-\Phi_{2}(P_{j})]G(P_{j}) \end{array}$$

yielding

$$\begin{array}{l} E[{\overset{⌣}{F}}_{4}(A,B;C,D|P)]\approx \frac{E[{\hat{F}}_{4}(A,B;C,D)]}{E[\overset{⌣}{G}(P)]}\\ =\frac{F_{4}(A,B,C,D)}{\left(1/J)\sum_{j=1}^{J}\frac{\left[1-\Phi_{2}(P_{j})][\sum_{k=1}^{N(P_{j})}m_{k}\right]}{\sum_{k=1}^{N(P_{j})}m_{k}-1}G(P_{j}\right)}\\ =\frac{F_{4}(A,B,C,D)}{Y(P)}. \end{array}$$

It follows that the approximate bias is

$$\begin{array}{l} \text{Bias}[{\overset{⌣}{F}}_{4}(A,B;C,D|P)]=E[{\overset{⌣}{F}}_{4}(A,B;C,D|P)]-F_{4}(A,B;C,D|P)\\ \approx [\frac{1}{Y(P)}-\frac{1}{G(P)}]F_{4}(A,B;C,D), \end{array}$$

and because $\Phi_{2}(P_{j})\geq 1/\sum_{k=1}^{N(P_{j})}m_{k}$, then 0<Y(P)≤G(P) making this is an upward approximate bias. □

**Lemma 17.** Consider *J* polymorphic loci in populations *A*, *B*, *C*, and *D* with respective parametric reference allele frequencies $a_{j},b_{j},c_{j},d_{j}\in (0,1)$, and suppose we take a random sample of $N(P_{j})$ individuals at locus *j* in population P∈{A,B,C,D}, some of which may be related or inbred. The estimator $\hat{H}(A,B,C,D)$ is unbiased. 
*Proof*. We first calculate

$$\begin{array}{l} E[\hat{H}(A_{j},B_{j},C_{j},D_{j})]=E[({\hat{a}}_{j}+{\hat{b}}_{j}-2{\hat{a}}_{j}{\hat{b}}_{j})({\hat{c}}_{j}+{\hat{d}}_{j}-2{\hat{c}}_{j}{\hat{d}}_{j})]\\ =E[({\hat{a}}_{j}+{\hat{b}}_{j}-2{\hat{a}}_{j}{\hat{b}}_{j})]E[({\hat{c}}_{j}+{\hat{d}}_{j}-2{\hat{c}}_{j}{\hat{d}}_{j})]\\ =(E[{\hat{a}}_{j}]+E[{\hat{b}}_{j}]-2E[{\hat{a}}_{j}]E[{\hat{b}}_{j}])(E[{\hat{c}}_{j}]+E[{\hat{d}}_{j}]-2E[{\hat{c}}_{j}]E[{\hat{d}}_{j}])\\ =(a_{j}+b_{j}-2a_{j}b_{j})(c_{j}+d_{j}-2c_{j}d_{j})\\ =H(A_{j},B_{j},C_{j},D_{j}). \end{array}$$

We show that $\hat{H}(A,B,C,D)$ is unbiased estimator of H(A,B,C,D) because

$$\begin{array}{l} E[\hat{H}(A,B,C,D)]=\frac{1}{J}\sum_{j=1}^{J}E[\hat{H}(A_{j},B_{j},C_{j},D_{j})]\\ =\frac{1}{J}\sum_{j=1}^{J}H(A_{j},B_{j},C_{j},D_{j})\\ =H(A,B,C,D).\;□ \end{array}$$

*Proof of Proposition 16*. Assuming that the expectation of $\hat{D}(A,B,C,D)$ is approximately equal to the ratio of expectations of $-{\hat{F}}_{4}(A,B;C,D)$ and $\hat{H}(A,B,C,D),\;\hat{D}(A,B,C,D)$ is an approximately unbiased estimator of D(A,B,C,D) because

$$\begin{array}{l} E[\hat{D}(A,B,C,D)]=-E[\frac{{\hat{F}}_{4}(A,B;C,D)}{\hat{H}(A,B,C,D)}]\\ \approx -\frac{E[{\hat{F}}_{4}(A,B;C,D)]}{E[\hat{H}(A,B,C,D)]}\\ =-\frac{F_{4}(A,B;C,D)}{H(A,B,C,D)}\\ =D(A,B,C,D).\;□ \end{array}$$

**Lemma 18.** Consider *J* independent polymorphic loci in a population *P* with parametric reference allele frequencies $p_{j}\in (0,1)$, and suppose we take a random sample of $N(P_{j})$ individuals at locus *j*, some of which may be related or inbred, where individual $k\in \{1,2,\ldots ,N(P_{j})$} has ploidy *m_k_*. Moreover, assume that no individual is related to more than one other individual, which makes the terms $\Phi_{3}(P_{j}),\;\Phi_{4}(P_{j}),\;\Phi_{2,2}(P_{j})$, and $\Phi_{2}{\left(P_{j}\right)}^{2}$ negligible to $\Phi_{2}(P_{j})$. Based on this simplifying assumption, the estimator $\hat{G}(P)$ has an approximate variance

$$\mathrm{Var}[\hat{G}(P)]\approx \frac{1}{J^{2}}\sum_{j=1}^{J}\Phi_{2}(P_{j})G(P_{j})-\frac{4}{J^{2}}\sum_{j=1}^{J}\Phi_{2}(P_{j})G{\left(P_{j}\right)}^{2}.$$

Moreover, the respective approximate variance for the unbiased estimator $\tilde{G}(P)$ and the estimator $\overset{⌣}{G}(P)$ are

$$\begin{array}{l} Var[\tilde{G}(P)]\approx \frac{1}{J^{2}}\sum_{j=1}^{J}\frac{\Phi_{2}(P_{j})}{1-2\Phi_{2}(P_{j})}G(P_{j})-\frac{4}{J^{2}}\sum_{j=1}^{J}\frac{\Phi_{2}(P_{j})}{1-2\Phi_{2}(P_{j})}G{\left(P_{j}\right)}^{2}\\ Var[\overset{⌣}{G}(P)]\approx \frac{1}{J^{2}}\sum_{j=1}^{J}\Phi_{2}(P_{j}){\left[\frac{\sum_{k=1}^{N(P_{j})}m_{k}}{\sum_{k=1}^{N(P_{j})}m_{k}-1}\right]}^{2}G(P_{j})-\frac{4}{J^{2}}\sum_{j=1}^{J}\Phi_{2}(P_{j}){\left[\frac{\sum_{k=1}^{N(P_{j})}m_{k}}{\sum_{k=1}^{N(P_{j})}m_{k}-1}\right]}^{2}G{\left(P_{j}\right)}^{2}. \end{array}$$

*Proof*. From the proof of Lemma 1, we have

$$E[\hat{G}(P_{j})]=[1-\Phi_{2}(P_{j})]G(P_{j})$$

and we calculate

$$\begin{array}{l} E[\hat{G}{\left(P_{j}\right)}^{2}]=E[{\hat{p}}_{j}^{2}{\left(1-{\hat{p}}_{j}\right)}^{2}]\\ =E[{\hat{p}}_{j}^{2}]-2E[{\hat{p}}_{j}^{3}]+E[{\hat{p}}_{j}^{4}]\\ \approx p_{j}^{2}+\Phi_{2}(P_{j})p_{j}(1-p_{j})-2[p_{j}^{3}+3\Phi_{2}(P_{j})p_{j}^{2}(1-p_{j})]+p_{j}^{4}+6\Phi_{2}(P_{j})p_{j}^{3}(1-p_{j})\\ =p_{j}^{2}-2p_{j}^{3}+p_{j}^{4}+\Phi_{2}(P_{j})p_{j}(1-p_{j})[1-6p_{j}+6p_{j}^{2}]\\ =p_{j}^{2}{\left(1-p_{j}\right)}^{2}+\Phi_{2}(P_{j})p_{j}(1-p_{j})[1-6p_{j}(1-p_{j})]\\ =G{\left(P_{j}\right)}^{2}+\Phi_{2}(P_{j})G(P_{j})[1-6G(P_{j})]\\ =\Phi_{2}(P_{j})G(P_{j})+[1-6\Phi_{2}(P_{j})]G{\left(P_{j}\right)}^{2}. \end{array}$$

Therefore, we have that

$$\begin{array}{l} Var[\hat{G}(P_{j})]=E[\hat{G}{\left(P_{j}\right)}^{2}]-E{\left[\hat{G}(P_{j})\right]}^{2}\\ \approx \Phi_{2}(P_{j})G(P_{j})+[1-6\Phi_{2}(P_{j})]G{\left(P_{j}\right)}^{2}-{\left[1-\Phi_{2}(P_{j})\right]}^{2}G{\left(P_{j}\right)}^{2}\\ =\Phi_{2}(P_{j})G(P_{j})+[1-6\Phi_{2}(P_{j})]G{\left(P_{j}\right)}^{2}-[1-2\Phi_{2}(P_{j})+\Phi_{2}{\left(P_{j}\right)}^{2}]G{\left(P_{j}\right)}^{2}\\ \approx \Phi_{2}(P_{j})G(P_{j})+[1-6\Phi_{2}(P_{j})]G{\left(P_{j}\right)}^{2}-[1-2\Phi_{2}(P_{j})]G{\left(P_{j}\right)}^{2}\\ =\Phi_{2}(P_{j})G(P_{j})-4\Phi_{2}(P_{j})G{\left(P_{j}\right)}^{2}, \end{array}$$

which gives

$$\begin{array}{l} Var[\hat{G}(P)]=\mathrm{Var}[\frac{1}{J}\sum_{j=1}^{J}\hat{G}(P_{j})]\\ =\frac{1}{J^{2}}\sum_{j=1}^{J}Var[\hat{G}(P_{j})]\\ \approx \frac{1}{J^{2}}\sum_{j=1}^{J}\Phi_{2}(P_{j})G(P_{j})-\frac{4}{J^{2}}\sum_{j=1}^{J}\Phi_{2}(P_{j})G{\left(P_{j}\right)}^{2}. \end{array}$$

Recall that

$$\begin{array}{l} \tilde{G}(P)=\frac{1}{J}\sum_{j=1}^{J}\tilde{G}(P_{j})\\ \overset{⌣}{G}(P)=\frac{1}{J}\sum_{j=1}^{J}\overset{⌣}{G}(P_{j}), \end{array}$$

where

$$\begin{array}{l} \tilde{G}(P_{j})=\frac{1}{1-\Phi_{2}(P_{j})}\hat{G}(P_{j})\\ \overset{⌣}{G}(P_{j})=\frac{\sum_{k=1}^{N(P_{j})}m_{k}}{\sum_{k=1}^{N(P_{j})}m_{k}-1}\hat{G}(P_{j}). \end{array}$$

It follows that

$$\begin{array}{l} Var[\tilde{G}(P)]=\mathrm{Var}[\frac{1}{J}\sum_{j=1}^{J}\tilde{G}(P_{j})]\\ =\frac{1}{J^{2}}\sum_{j=1}^{J}Var[\tilde{G}(P_{j})]\\ =\frac{1}{J^{2}}\sum_{j=1}^{J}\frac{1}{{\left[1-\Phi_{2}(P_{j})\right]}^{2}}Var[\hat{G}(P_{j})]\\ \approx \frac{1}{J^{2}}\sum_{j=1}^{J}\frac{\Phi_{2}(P_{j})}{1-2\Phi_{2}(P_{j})}G(P_{j})-\frac{4}{J^{2}}\sum_{j=1}^{J}\frac{\Phi_{2}(P_{j})}{1-2\Phi_{2}(P_{j})}G{\left(P_{j}\right)}^{2} \end{array}$$

and similarly

$$\begin{array}{l} Var[\overset{⌣}{G}(P)]=\mathrm{Var}[\frac{1}{J}\sum_{j=1}^{J}\overset{⌣}{G}(P_{j})]\\ =\frac{1}{J^{2}}\sum_{j=1}^{J}Var[\overset{⌣}{G}(P_{j})]\\ =\frac{1}{J^{2}}\sum_{j=1}^{J}[\frac{\sum_{k=1}^{N(P_{j})}m_{k}}{\sum_{k=1}^{N(P_{j})}m_{k}-1}{]}^{2}Var[\hat{G}(P_{j})]\\ \approx \frac{1}{J^{2}}\sum_{j=1}^{J}\Phi_{2}(P_{j}){\left[\frac{\sum_{k=1}^{N(P_{j})}m_{k}}{\sum_{k=1}^{N(P_{j})}m_{k}-1}\right]}^{2}G(P_{j})-\frac{4}{J^{2}}\sum_{j=1}^{J}\Phi_{2}(P_{j}){\left[\frac{\sum_{k=1}^{N(P_{j})}m_{k}}{\sum_{k=1}^{N(P_{j})}m_{k}-1}\right]}^{2}G{\left(P_{j}\right)}^{2}.\;□ \end{array}$$

**Lemma 19.** Consider *J* independent polymorphic loci in populations *A* and *B* with respective parametric reference allele frequencies $a_{j},b_{j}\in (0,1)$, and suppose we take a random sample of $N(P_{j})$ individuals at locus *j* in population P∈{A,B}, some of which may be related or inbred. Moreover, assume that no individual is related to more than one other individual, which makes the terms $\Phi_{3}(P_{j}),\;\Phi_{4}(P_{j}),\;\Phi_{2,2}(P_{j})$, and $\Phi_{2}{\left(P_{j}\right)}^{2}$ negligible to $\Phi_{2}(P_{j})$. Based on this simplifying assumption, the estimators ${\hat{F}}_{2}(A,B)$ and $\hat{G}(B)$ have an approximate covariance

$$\begin{array}{l} Cov[{\hat{F}}_{2}(A,B),\hat{G}(B)]\approx \frac{2}{J^{2}}\sum_{j=1}^{J}\Phi_{2}(B_{j})G{\left(B_{j}\right)}^{2}-\frac{2}{J^{2}}\sum_{j=1}^{J}\Phi_{2}(B_{j})G(A_{j})G(B_{j})\\ -\frac{2}{J^{2}}\sum_{j=1}^{J}\Phi_{2}(B_{j})F_{2}(A_{j},B_{j})G(B_{j}). \end{array}$$

*Proof*. From the proofs of Lemma 1 and Proposition 3, we have

$$E[\hat{G}(B_{j})]=[1-\Phi_{2}(B_{j})]G(B_{j})$$

and

$$E[{\hat{F}}_{2}(A_{j},B_{j})]=F_{2}(A_{j},B_{j})+\Phi_{2}(A_{j})G(A_{j})+\Phi_{2}(B_{j})G(B_{j}),$$

yielding

$$\begin{array}{l} E[{\hat{F}}_{2}(A_{j},B_{j})]E[\hat{G}(B_{j})]=[F_{2}(A_{j},B_{j})+\Phi_{2}(A_{j})G(A_{j})+\Phi_{2}(B_{j})G(B_{j})][1-\Phi_{2}(B_{j})]G(B_{j})\\ =F_{2}(A_{j},B_{j})G(B_{j})+\Phi_{2}(A_{j})G(A_{j})G(B_{j})+\Phi_{2}(B_{j})G{\left(B_{j}\right)}^{2}\\ -\Phi_{2}(B_{j})F_{2}(A_{j},B_{j})G(B_{j})-\Phi_{2}(A_{j})\Phi_{2}(B_{j})G(A_{j})G(B_{j})\\ -\Phi_{2}{\left(B_{j}\right)}^{2}G{\left(B_{j}\right)}^{2}\\ \approx F_{2}(A_{j},B_{j})G(B_{j})+\Phi_{2}(A_{j})G(A_{j})G(B_{j})+\Phi_{2}(B_{j})G{\left(B_{j}\right)}^{2}\\ -\Phi_{2}(B_{j})F_{2}(A_{j},B_{j})G(B_{j})-\Phi_{2}(A_{j})\Phi_{2}(B_{j})G(A_{j})G(B_{j}), \end{array}$$

where we used the fact that $\Phi_{2}{\left(B_{j}\right)}^{2}$ is negligible compared to $\Phi_{2}(B_{j})$ as an approximation. We also calculate

$$\begin{array}{l} E[{\hat{F}}_{2}(A_{j},B_{j})\hat{G}(B_{j})]=E[{\left({\hat{a}}_{j}-{\hat{b}}_{j}\right)}^{2}{\hat{b}}_{j}(1-{\hat{b}}_{j})]\\ =E[({\hat{a}}_{j}^{2}-2{\hat{a}}_{j}{\hat{b}}_{j}+{\hat{b}}_{j}^{2})({\hat{b}}_{j}-{\hat{b}}_{j}^{2})]\\ =E[{\hat{a}}_{j}^{2}({\hat{b}}_{j}-{\hat{b}}_{j}^{2})-2{\hat{a}}_{j}({\hat{b}}_{j}^{2}-{\hat{b}}_{j}^{3})+{\hat{b}}_{j}^{3}-{\hat{b}}_{j}^{4}]\\ =E[{\hat{a}}_{j}^{2}](E[{\hat{b}}_{j}]-E[{\hat{b}}_{j}^{2}])-2E[{\hat{a}}_{j}](E[{\hat{b}}_{j}^{2}]-E[{\hat{b}}_{j}^{3}])+E[{\hat{b}}_{j}^{3}]-E[{\hat{b}}_{j}^{4}]\\ \approx [a_{j}^{2}+\Phi_{2}(A_{j})a_{j}(1-a_{j})][b_{j}-b_{j}^{2}-\Phi_{2}(B_{j})b_{j}(1-b_{j})]\\ -2a_{j}[b_{j}^{2}+\Phi_{2}(B_{j})b_{j}(1-b_{j})-b_{j}^{3}-3\Phi_{2}(B_{j})b_{j}^{2}(1-b_{j})]\\ +b_{j}^{3}+3\Phi_{2}(B_{j})b_{j}^{2}(1-b_{j})-b_{j}^{4}-6\Phi_{2}(B_{j})b_{j}^{3}(1-b_{j}). \end{array}$$

Recognizing that $G(A_{j})=a_{j}(1-a_{j}),\;G(B_{j})=b_{j}(1-b_{j})$, and $F_{2}(A_{j},B_{j})=a_{j}^{2}-2a_{j}b_{j}+b_{j}^{2}$, we have

$$\begin{array}{l} E[{\hat{F}}_{2}(A_{j},B_{j})\hat{G}(B_{j})]\approx [a_{j}^{2}+\Phi_{2}(A_{j})G(A_{j})][1-\Phi_{2}(B_{j})]G(B_{j})\\ -2a_{j}[\Phi_{2}(B_{j})+[1-3\Phi_{2}(B_{j})]b_{j}]G(B_{j})\\ +[3\Phi_{2}(B_{j})b_{j}+[1-6\Phi_{2}(B_{j})]b_{j}^{2}]G(B_{j})\\ =G(B_{j})[a_{j}^{2}-\Phi_{2}(B_{j})a_{j}^{2}+\Phi_{2}(A_{j})G(A_{j})-\Phi_{2}(A_{j})\Phi_{2}(B_{j})G(A_{j})\\ -2\Phi_{2}(B_{j})a_{j}-2a_{j}b_{j}+2(3)\Phi_{2}(B_{j})a_{j}b_{j}+3\Phi_{2}(B_{j})b_{j}+b_{j}^{2}\\ -6\Phi_{2}(B_{j})b_{j}^{2}]\\ =G(B_{j})[F_{2}(A_{j},B_{j})-\Phi_{2}(B_{j})[3a_{j}^{2}-2(3)a_{j}b_{j}+3b_{j}^{2}-2a_{j}^{2}+3b_{j}^{2}]\\ -2\Phi_{2}(B_{j})a_{j}+3\Phi_{2}(B_{j})b_{j}+[\Phi_{2}(A_{j})-\Phi_{2}(A_{j})\Phi_{2}(B_{j})]G(A_{j})]\\ =G(B_{j})[F_{2}(A_{j},B_{j})-3\Phi_{2}(B_{j})[a_{j}^{2}-2a_{j}b_{j}+b_{j}^{2}]\\ -2\Phi_{2}(B_{j})[a_{j}-a_{j}^{2}]+3\Phi_{2}(B_{j})[b_{j}-b_{j}^{2}]\\ +[\Phi_{2}(A_{j})-\Phi_{2}(A_{j})\Phi_{2}(B_{j})]G(A_{j})]\\ =G(B_{j})[F_{2}(A_{j},B_{j})-3\Phi_{2}(B_{j})F_{2}(A_{j},B_{j})-2\Phi_{2}(B_{j})G(A_{j})+3\Phi_{2}(B_{j})G(B_{j})\\ +[\Phi_{2}(A_{j})-\Phi_{2}(A_{j})\Phi_{2}(B_{j})]G(A_{j})]\\ =[1-3\Phi_{2}(B_{j})]F_{2}(A_{j},B_{j})G(B_{j})+3\Phi_{2}(B_{j})G{\left(B_{j}\right)}^{2}\\ +[\Phi_{2}(A_{j})-2\Phi_{2}(B_{j})-\Phi_{2}(A_{j})\Phi_{2}(B_{j})]G(A_{j})G(B_{j}). \end{array}$$

Therefore, we have that

$$\begin{array}{l} Cov[{\hat{F}}_{2}(A_{j},B_{j}),\hat{G}(B_{j})]=E[{\hat{F}}_{2}(A_{j},B_{j})\hat{G}(B_{j})]-E[{\hat{F}}_{2}(A_{j},B_{j})]E[\hat{G}(B_{j})]\\ \approx [1-3\Phi_{2}(B_{j})]F_{2}(A_{j},B_{j})G(B_{j})+3\Phi_{2}(B_{j})G{\left(B_{j}\right)}^{2}\\ +[\Phi_{2}(A_{j})-2\Phi_{2}(B_{j})-\Phi_{2}(A_{j})\Phi_{2}(B_{j})]G(A_{j})G(B_{j})\\ -[F_{2}(A_{j},B_{j})G(B_{j})+\Phi_{2}(A_{j})G(A_{j})G(B_{j})+\Phi_{2}(B_{j})G{\left(B_{j}\right)}^{2}\\ -\Phi_{2}(B_{j})F_{2}(A_{j},B_{j})G(B_{j})-\Phi_{2}(A_{j})\Phi_{2}(B_{j})G(A_{j})G(B_{j})]\\ =2\Phi_{2}(B_{j})G{\left(B_{j}\right)}^{2}-2\Phi_{2}(B_{j})G(A_{j})G(B_{j})-2\Phi_{2}(B_{j})F_{2}(A_{j},B_{j})G(B_{j})\\ =2\Phi_{2}(B_{j})G(B_{j})[G(B_{j})-G(A_{j})-F_{2}(A_{j},B_{j})], \end{array}$$

which gives

$$\begin{array}{l} Cov[{\hat{F}}_{2}(A,B),\hat{G}(B)]=\mathrm{Cov}[\frac{1}{J}\sum_{j=1}^{J}{\hat{F}}_{2}(A_{j},B_{j}),\frac{1}{J}\sum_{j=1}^{J}\hat{G}(B_{j})]\\ =\frac{1}{J^{2}}\sum_{j=1}^{J}Cov[{\hat{F}}_{2}(A_{j},B_{j}),\hat{G}(B_{j})]\\ \approx \frac{2}{J^{2}}\sum_{j=1}^{J}\Phi_{2}(B_{j})G{\left(B_{j}\right)}^{2}-\frac{2}{J^{2}}\sum_{j=1}^{J}\Phi_{2}(B_{j})G(A_{j})G(B_{j})\\ -\frac{2}{J^{2}}\sum_{j=1}^{J}\Phi_{2}(B_{j})F_{2}(A_{j},B_{j})G(B_{j}).\;□ \end{array}$$

**Proposition 20.** Consider *J* independent polymorphic loci in a populations *A* and *B* with respective parametric reference allele frequencies $a_{j},b_{j}\in (0,1)$, and suppose we take a random sample of $N(P_{j})$ individuals at locus *j* in population P∈{A,B}, some of which may be related or inbred. Moreover, assume that no individual is related to more than one other individual, which makes the terms $\Phi_{3}(P_{j}),\;\Phi_{4}(P_{j}),\;\Phi_{2,2}(P_{j}),\;\Phi_{2}{\left(P_{j}\right)}^{2}$, and $\Phi_{2}(A_{j})\Phi_{2}(B_{j})$ negligible to $\Phi_{2}(P_{j})$. Based on this simplifying assumption, the estimator ${\hat{F}}_{2}(A,B)$ has approximate variance

$$\mathrm{Var}[{\hat{F}}_{2}(A,B)]\approx \frac{4}{J^{2}}\sum_{j=1}^{J}\Phi_{2}(A_{j})F_{2}(A_{j},B_{j})G(A_{j})+\frac{4}{J^{2}}\sum_{j=1}^{J}\Phi_{2}(B_{j})F_{2}(A_{j},B_{j})G(B_{j}).$$

*Proof*. From the proof of Proposition 3, we have

$$E[{\hat{F}}_{2}(A_{j},B_{j})]=F_{2}(A_{j},B_{j})+\Phi_{2}(A_{j})G(A_{j})+\Phi_{2}(B_{j})G(B_{j}),$$

which gives

$$\begin{array}{l} E{\left[{\hat{F}}_{2}(A_{j},B_{j})\right]}^{2}={\left[F_{2}(A_{j},B_{j})+\Phi_{2}(A_{j})G(A_{j})+\Phi_{2}(B_{j})G(B_{j})\right]}^{2}\\ =F_{2}{\left(A_{j},B_{j}\right)}^{2}+2\Phi_{2}(A_{j})F_{2}(A_{j},B_{j})G(A_{j})+2\Phi_{2}(B_{j})F_{2}(A_{j},B_{j})G(B_{j})\\ +2\Phi_{2}(A_{j})\Phi_{2}(B_{j})G(A_{j})G(B_{j})+\Phi_{2}{\left(A_{j}\right)}^{2}G{\left(A_{j}\right)}^{2}+\Phi_{2}{\left(B_{j}\right)}^{2}G(B_{j})\\ \approx F_{2}{\left(A_{j},B_{j}\right)}^{2}+2\Phi_{2}(A_{j})F_{2}(A_{j},B_{j})G(A_{j})+2\Phi_{2}(B_{j})F_{2}(A_{j},B_{j})G(B_{j}), \end{array}$$

where we used the fact that $\Phi_{2}{\left(A_{j}\right)}^{2},\;\Phi_{2}(B_{j})$, and $\Phi_{2}(A_{j})\Phi_{2}(B_{j})$ are negligible compared to $\Phi_{2}(A_{j})$ and $\Phi_{2}(B_{j})$ as an approximation. We also calculate

$$\begin{array}{l} E[{\hat{F}}_{2}{\left(A_{j},B_{j}\right)}^{2}]=E[{\left({\hat{a}}_{j}-{\hat{b}}_{j}\right)}^{4}]\\ =E[{\hat{a}}_{j}^{4}]-4E[{\hat{a}}_{j}^{3}]E[{\hat{b}}_{j}]+6E[{\hat{a}}_{j}^{2}]E[{\hat{b}}_{j}^{2}]-4E[{\hat{a}}_{j}]E[{\hat{b}}_{j}^{3}]+E[{\hat{b}}_{j}^{4}]\\ \approx a_{j}^{4}+6\Phi_{2}(A_{j})a_{j}^{3}(1-a_{j})-4[a_{j}^{3}+3\Phi_{2}(A_{j})a_{j}^{2}(1-a_{j})]b_{j}\\ +6[a_{j}^{2}+\Phi_{2}(A_{j})a_{j}(1-a_{j})][b_{j}^{2}+\Phi_{2}(B_{j})b_{j}(1-b_{j})]\\ -4a_{j}[b_{j}^{3}+3\Phi_{2}(B_{j})b_{j}^{2}(1-b_{j})]+b_{j}^{4}+6\Phi_{2}(B_{j})b_{j}^{3}(1-b_{j})\\ =a_{j}^{4}-4a_{j}^{3}b_{j}+6a_{j}^{2}b_{j}^{2}-4a_{j}b_{j}^{3}+b_{j}^{4}+6\Phi_{2}(A_{j})a_{j}(1-a_{j})[a_{j}^{2}-2a_{j}b_{j}+b_{j}^{2}]\\ +6\Phi_{2}(B_{j})b_{j}(1-b_{j})[a_{j}^{2}-2a_{j}b_{j}+b_{j}^{2}]+6\Phi_{2}(A_{j})\Phi_{2}(B_{j})a_{j}(1-a_{j})b_{j}(1-b_{j})\\ ={\left(a_{j}-b_{j}\right)}^{4}+6\Phi_{2}(A_{j})a_{j}(1-a_{j}){\left(a_{j}-b_{j}\right)}^{2}+6\Phi_{2}(B_{j})b_{j}(1-b_{j}){\left(a_{j}-b_{j}\right)}^{2}\\ +6\Phi_{2}(A_{j})\Phi_{2}(B_{j})a_{j}(1-a_{j})b_{j}(1-b_{j})\\ =F_{2}{\left(A_{j},B_{j}\right)}^{2}+6\Phi_{2}(A_{j})F_{2}(A_{j},B_{j})G(A_{j})+6\Phi_{2}(B_{j})F_{2}(A_{j},B_{j})G(B_{j})\\ +6\Phi_{2}(A_{j})\Phi_{2}(B_{j})G(A_{j})G(B_{j})\\ \approx F_{2}{\left(A_{j},B_{j}\right)}^{2}+6\Phi_{2}(A_{j})F_{2}(A_{j},B_{j})G(A_{j})+6\Phi_{2}(B_{j})F_{2}(A_{j},B_{j})G(B_{j}). \end{array}$$

Therefore, we have that

$$\begin{array}{l} Var[{\hat{F}}_{2}(A_{j},B_{j})]=E[{\hat{F}}_{2}{\left(A_{j},B_{j}\right)}^{2}]-E{\left[{\hat{F}}_{2}(A_{j},B_{j})\right]}^{2}\\ \approx F_{2}{\left(A_{j},B_{j}\right)}^{2}+6\Phi_{2}(A_{j})F_{2}(A_{j},B_{j})G(A_{j})+6\Phi_{2}(B_{j})F_{2}(A_{j},B_{j})G(B_{j})\\ -[F_{2}{\left(A_{j},B_{j}\right)}^{2}+2\Phi_{2}(A_{j})F_{2}(A_{j},B_{j})G(A_{j})+2\Phi_{2}(B_{j})F_{2}(A_{j},B_{j})G(B_{j})]\\ =4\Phi_{2}(A_{j})F_{2}(A_{j},B_{j})G(A_{j})+4\Phi_{2}(B_{j})F_{2}(A_{j},B_{j})G(B_{j}), \end{array}$$

which gives

$$\begin{array}{l} Var[{\hat{F}}_{2}(A,B)]=\mathrm{Var}[\frac{1}{J}\sum_{j=1}^{J}{\hat{F}}_{2}(A_{j},B_{j})]\\ =\frac{1}{J^{2}}\sum_{j=1}^{J}Var[{\hat{F}}_{2}(A_{j},B_{j})]\\ \approx \frac{4}{J^{2}}\sum_{j=1}^{J}\Phi_{2}(A_{j})F_{2}(A_{j},B_{j})G(A_{j})+\frac{4}{J^{2}}\sum_{j=1}^{J}\Phi_{2}(B_{j})F_{2}(A_{j},B_{j})G(B_{j}).\;□ \end{array}$$

**Proposition 21.** Consider *J* independent polymorphic loci in a populations *A* and *B* with respective parametric reference allele frequencies $a_{j},b_{j}\in (0,1)$, and suppose we take a random sample of $N(P_{j})$ individuals at locus *j* in population P∈{A,B}, some of which may be related or inbred. Moreover, assume that no individual is related to more than one other individual, which makes the terms $\Phi_{3}(P_{j}),\;\Phi_{4}(P_{j}),\;\Phi_{2,2}(P_{j}),\;\Phi_{2}{\left(P_{j}\right)}^{2}$, and $\Phi_{2}(A_{j})\Phi_{2}(B_{j})$ negligible to $\Phi_{2}(P_{j})$. Based on this simplifying assumption, the unbiased estimator ${\tilde{F}}_{2}(A,B)$ has approximate variance

$$\mathrm{Var}[{\tilde{F}}_{2}(A,B)]\approx \frac{4}{J^{2}}\sum_{j=1}^{J}\Phi_{2}(A_{j})F_{2}(A_{j},B_{j})G(A_{j})+\frac{4}{J^{2}}\sum_{j=1}^{J}\Phi_{2}(B_{j})F_{2}(A_{j},B_{j})G(B_{j}).$$

*Proof*. Recall that

$${\tilde{F}}_{2}(A_{j},B_{j})={\hat{F}}_{2}(A_{j},B_{j})-\Phi_{2}(A_{j})\tilde{G}(A_{j})-\Phi_{2}(B_{j})\tilde{G}(B_{j}),$$

where ${\tilde{F}}_{2}(A_{j},B_{j})$ is an unbiased estimator for $F_{2}(A_{j},B_{j})$ and $\tilde{G}(P_{j})$ is an unbiased estimator of $G(P_{j})$ for P∈{A,B} at locus j∈{1,2,…,J}. Also, from the proof of Proposition 3, we have

$$E[{\hat{F}}_{2}(A_{j},B_{j})]=F_{2}(A_{j},B_{j})+\Phi_{2}(A_{j})G(A_{j})+\Phi_{2}(B_{j})G(B_{j}).$$

Therefore, we have that

$$\begin{array}{l} Var[{\tilde{F}}_{2}(A_{j},B_{j})]=\mathrm{Var}[{\hat{F}}_{2}(A_{j},B_{j})-\Phi_{2}(A_{j})\tilde{G}(A_{j})-\Phi_{2}(B_{j})\tilde{G}(B_{j})]\\ =\mathrm{Var}[{\hat{F}}_{2}(A_{j},B_{j})]+\Phi_{2}{\left(A_{j}\right)}^{2}Var[\tilde{G}(A_{j})]+\Phi_{2}{\left(B_{j}\right)}^{2}Var[\tilde{G}(B_{j})]\\ -2\Phi_{2}(A_{j})\mathrm{Cov}[{\hat{F}}_{2}(A_{j},B_{j}),\tilde{G}(A_{j})]-2\Phi_{2}(B_{j})\mathrm{Cov}[{\hat{F}}_{2}(A_{j},B_{j}),\tilde{G}(B_{j})]\\ +2\Phi_{2}(A_{j})\Phi_{2}(B_{j})\mathrm{Cov}[\tilde{G}(A_{j}),\tilde{G}(B_{j})]\\ \approx \mathrm{Var}[{\hat{F}}_{2}(A_{j},B_{j})]-2\Phi_{2}(A_{j})\mathrm{Cov}[{\hat{F}}_{2}(A_{j},B_{j}),\tilde{G}(A_{j})]\\ -2\Phi_{2}(B_{j})\mathrm{Cov}[{\hat{F}}_{2}(A_{j},B_{j}),\tilde{G}(B_{j})], \end{array}$$

where we used the fact that $\Phi_{2}{\left(A_{j}\right)}^{2}$ and $\Phi_{2}{\left(B_{j}\right)}^{2}$ are negligible compared to $\Phi_{2}(A_{j})$ and $\Phi_{2}(B_{j})$ as an approximation, and where $\mathrm{Cov}[\tilde{G}(A_{j}),\tilde{G}(B_{j})]=0$ because drawing alleles in population *A* is independent of population *B*. Moreover, because $\tilde{G}(P_{j})=\hat{G}(P_{j})/[1-\Phi_{2}(P_{j})]$, we have

$$\begin{array}{l} Var[{\tilde{F}}_{2}(A_{j},B_{j})]\approx \mathrm{Var}[{\hat{F}}_{2}(A_{j},B_{j})]-\frac{2\Phi_{2}(A_{j})}{1-\Phi_{2}(A_{j})}Cov[{\hat{F}}_{2}(A_{j},B_{j}),\hat{G}(A_{j})]\\ -\frac{2\Phi_{2}(B_{j})}{1-\Phi_{2}(B_{j})}Cov[{\hat{F}}_{2}(A_{j},B_{j}),\hat{G}(B_{j})]\\ \approx 4\Phi_{2}(A_{j})F_{2}(A_{j},B_{j})G(A_{j})+4\Phi_{2}(B_{j})F_{2}(A_{j},B_{j})G(B_{j})\\ +4\Phi_{2}(A_{j})\Phi_{2}(B_{j})G(A_{j})G(B_{j})\\ -\frac{2\Phi_{2}(A_{j})}{1-\Phi_{2}(A_{j})}[2\Phi_{2}(A_{j})G{\left(A_{j}\right)}^{2}-2\Phi_{2}(A_{j})G(A_{j})G(B_{j})\\ -2\Phi_{2}(A_{j})F_{2}(A_{j},B_{j})G(A_{j})]\\ -\frac{2\Phi_{2}(B_{j})}{1-\Phi_{2}(B_{j})}[2\Phi_{2}(B_{j})G{\left(B_{j}\right)}^{2}-2\Phi_{2}(B_{j})G(A_{j})G(B_{j})\\ -2\Phi_{2}(B_{j})F_{2}(A_{j},B_{j})G(B_{j})]. \end{array}$$

Recalling the assumption that $\Phi_{2}{\left(A_{j}\right)}^{2},\;\Phi_{2}{\left(B_{j}\right)}^{2}$, and $\Phi_{2}(A_{j})\Phi_{2}(B_{j})$ are negligible compared to $\Phi_{2}(A_{j})$ and $\Phi_{2}(B_{j})$, we have

$$\begin{array}{l} Var[{\tilde{F}}_{2}(A_{j},B_{j})]\approx 4\Phi_{2}(A_{j})F_{2}(A_{j},B_{j})G(A_{j})+4\Phi_{2}(B_{j})F_{2}(A_{j},B_{j})G(B_{j})\\ \approx \mathrm{Var}[{\hat{F}}_{2}(A_{j},B_{j})], \end{array}$$

and it follows that

$$\mathrm{Var}[{\tilde{F}}_{2}(A,B)]\approx \mathrm{Var}[{\hat{F}}_{2}(A,B)].\;□$$

**Proposition 22.** Consider *J* independent polymorphic loci in a populations *A* and *B* with respective parametric reference allele frequencies $a_{j},b_{j}\in (0,1)$, and suppose we take a random sample of $N(P_{j})$ individuals at locus *j* in population P∈{A,B}, some of which may be related or inbred. Moreover, assume that no individual is related to more than one other individual, which makes the terms $\Phi_{3}(P_{j}),\;\Phi_{4}(P_{j}),\;\Phi_{2,2}(P_{j}),\;\Phi_{2}{\left(P_{j}\right)}^{2},\;\Phi_{2}(A_{j})\Phi_{2}(B_{j})$, and other terms of similar order negligible to $\Phi_{2}(P_{j})$. Based on this simplifying assumption, the estimator ${\overset{⌣}{F}}_{2}(A,B)$ has approximate variance

$$\mathrm{Var}[{\overset{⌣}{F}}_{2}(A,B)]\approx \frac{4}{J^{2}}\sum_{j=1}^{J}\Phi_{2}(A_{j})F_{2}(A_{j},B_{j})G(A_{j})+\frac{4}{J^{2}}\sum_{j=1}^{J}\Phi_{2}(B_{j})F_{2}(A_{j},B_{j})G(B_{j}).$$

*Proof*. Recall that

$${\overset{⌣}{F}}_{2}(A_{j},B_{j})={\hat{F}}_{2}(A_{j},B_{j})-\frac{1}{\sum_{k=1}^{N(A_{j})}m_{k}-1}\hat{G}(A_{j})-\frac{1}{\sum_{k=1}^{N(A_{j})}m_{k}-1}\hat{G}(A_{j}),$$

where ${\overset{⌣}{F}}_{2}(A_{j},B_{j})$ and ${\hat{F}}_{2}(A_{j},B_{j})$ are estimators for $F_{2}(A_{j},B_{j})$ and $\hat{G}(P_{j})$ is an estimator of $G(P_{j})$ for P∈{A,B} at locus j∈{1,2,…,J}. Also, from the proof of Proposition 3, we have

$$E[{\hat{F}}_{2}(A_{j},B_{j})]=F_{2}(A_{j},B_{j})+\Phi_{2}(A_{j})G(A_{j})+\Phi_{2}(B_{j})G(B_{j}).$$

Therefore, we have that

$$\begin{array}{l} Var[{\overset{⌣}{F}}_{2}(A_{j},B_{j})]=\mathrm{Var}[{\hat{F}}_{2}(A_{j},B_{j})-\frac{1}{\sum_{k=1}^{N(A_{j})}m_{k}-1}\hat{G}(A_{j})-\frac{1}{\sum_{k=1}^{N(A_{j})}m_{k}-1}\hat{G}(A_{j})]\\ =\mathrm{Var}[{\hat{F}}_{2}(A_{j},B_{j})]+{\left[\frac{1}{\sum_{k=1}^{N(A_{j})}m_{k}-1}\right]}^{2}Var[\hat{G}(A_{j})]+{\left[\frac{1}{\sum_{k=1}^{N(B_{j})}m_{k}-1}\right]}^{2}Var[\hat{G}(B_{j})]\\ -2[\frac{1}{\sum_{k=1}^{N(A_{j})}m_{k}-1}]\mathrm{Cov}[{\hat{F}}_{2}(A_{j},B_{j}),\hat{G}(A_{j})]-2[\frac{1}{\sum_{k=1}^{N(B_{j})}m_{k}-1}]\mathrm{Cov}[{\hat{F}}_{2}(A_{j},B_{j}),\hat{G}(B_{j})]\\ +2[\frac{1}{\sum_{k=1}^{N(A_{j})}m_{k}-1}][\frac{1}{\sum_{k=1}^{N(B_{j})}m_{k}-1}]\mathrm{Cov}[\hat{G}(A_{j}),\hat{G}(B_{j})]\\ \approx \mathrm{Var}[{\hat{F}}_{2}(A_{j},B_{j})]-2[\frac{1}{\sum_{k=1}^{N(A_{j})}m_{k}-1}]\mathrm{Cov}[{\hat{F}}_{2}(A_{j},B_{j}),\hat{G}(A_{j})]\\ -2[\frac{1}{\sum_{k=1}^{N(B_{j})}m_{k}-1}]\mathrm{Cov}[{\hat{F}}_{2}(A_{j},B_{j}),\hat{G}(B_{j})], \end{array}$$

where we used the fact that $\Phi_{2}{\left(A_{j}\right)}^{2}$ and $\Phi_{2}{\left(B_{j}\right)}^{2}$ are negligible compared to $\Phi_{2}(A_{j})$ and $\Phi_{2}(B_{j})$ as an approximation (and hence $1/[\sum_{k=1}^{N(P_{j})}m_{k}-1]$ assuming $N(P_{j})$ is large enough), and where $\mathrm{Cov}[\hat{G}(A_{j}),\hat{G}(B_{j})]=0$ because drawing alleles in population *A* is independent of population *B*. Moreover, because

$$\begin{array}{l} Cov[{\hat{F}}_{2}(A_{j},B_{j}),\hat{G}(A_{j})]\approx \Phi_{2}(A_{j})F_{2}(A_{j},B_{j})G(A_{j})\\ Cov[{\hat{F}}_{2}(A_{j},B_{j}),\hat{G}(B_{j})]\approx \Phi_{2}(B_{j})F_{2}(A_{j},B_{j})G(B_{j}) \end{array}$$

we have

$$\begin{array}{l} {}[\frac{1}{\sum_{k=1}^{N(A_{j})}m_{k}-1}]\mathrm{Cov}[{\hat{F}}_{2}(A_{j},B_{j}),\hat{G}(A_{j})]\approx 0\\ {}[\frac{1}{\sum_{k=1}^{N(B_{j})}m_{k}-1}]\mathrm{Cov}[{\hat{F}}_{2}(A_{j},B_{j}),\hat{G}(B_{j})]\approx 0 \end{array}$$

by recalling the assumption that $\Phi_{2}{\left(A_{j}\right)}^{2},\;\Phi_{2}{\left(B_{j}\right)}^{2}$, or any other term of similar magnitude (such as the $\Phi_{2}(P_{j})/[\sum_{k=1}^{N(P_{j})}m_{k}-1]$ terms that appear for populations P∈{A,B}) and $\Phi_{2}(A_{j})\Phi_{2}(B_{j})$ are negligible compared to $\Phi_{2}(A_{j})$ and $\Phi_{2}(B_{j})$. Because of this, we have

$$\mathrm{Var}[{\overset{⌣}{F}}_{2}(A_{j},B_{j})]\approx \mathrm{Var}[{\hat{F}}_{2}(A_{j},B_{j})],$$

and it follows that

$$\mathrm{Var}[{\overset{⌣}{F}}_{2}(A,B)]\approx \mathrm{Var}[{\hat{F}}_{2}(A,B)].\;□$$

**Proposition 23.** Consider *J* independent polymorphic loci in populations *A*, *B*, and *C* with respective parametric reference allele frequencies $a_{j},b_{j},c_{j}\in (0,1)$, and suppose we take a random sample of $N(P_{j})$ individuals at locus *j* in population P∈{A,B,C}, some of which may be related or inbred. Moreover, assume that no individual is related to more than one other individual, which makes the terms $\Phi_{3}(P_{j}),\;\Phi_{4}(P_{j}),\;\Phi_{2,2}(P_{j}),\;\Phi_{2}{\left(P_{j}\right)}^{2}$, $\Phi_{2}(A_{j})\Phi_{2}(B_{j}),\;\Phi_{2}(A_{j})\Phi_{2}(C_{j})$, and $\Phi_{2}(B_{j})\Phi_{2}(C_{j})$ negligible to $\Phi_{2}(P_{j})$. Based on this simplifying assumption, the estimator ${\hat{F}}_{3}(A;B,C)$ has approximate variance

$$\begin{array}{l} Var[{\hat{F}}_{3}(A;B,C)]\approx \frac{4}{J^{2}}\sum_{j=1}^{J}\Phi_{2}(A_{j})F_{3}(A_{j};B_{j},C_{j})G(A_{j})+\frac{1}{J^{2}}\sum_{j=1}^{J}\Phi_{2}(A_{j})F_{2}(B_{j},C_{j})G(A_{j})\\ +\frac{1}{J^{2}}\sum_{j=1}^{J}\Phi_{2}(B_{j})F_{2}(A_{j},C_{j})G(B_{j})+\frac{1}{J^{2}}\sum_{j=1}^{J}\Phi_{2}(C_{j})F_{2}(A_{j},B_{j})G(C_{j}). \end{array}$$

*Proof*. From the proof of Proposition 6, we have

$$E[{\hat{F}}_{3}(A_{j};B_{j},C_{j})]=F_{3}(A_{j};B_{j},C_{j})+\Phi_{2}(A_{j})G(A_{j}),$$

which gives

$$\begin{array}{l} E{\left[{\hat{F}}_{3}(A_{j};B_{j},C_{j})\right]}^{2}={\left[F_{3}(A_{j};B_{j},C_{j})+\Phi_{2}(A_{j})G(A_{j})\right]}^{2}\\ =F_{3}{\left(A_{j};B_{j},C_{j}\right)}^{2}+2\Phi_{2}(A_{j})F_{3}(A_{j};B_{j},C_{j})G(A_{j})+\Phi_{2}{\left(A_{j}\right)}^{2}G{\left(A_{j}\right)}^{2}\\ \approx F_{3}{\left(A_{j};B_{j},C_{j}\right)}^{2}+2\Phi_{2}(A_{j})F_{3}(A_{j};B_{j},C_{j})G(A_{j}), \end{array}$$

where we used the fact that $\Phi_{2}{\left(A_{j}\right)}^{2}$ is negligible compared to $\Phi_{2}(A_{j})$ as an approximation. We also calculate

$$\begin{array}{l} E[{\hat{F}}_{3}{\left(A_{j};B_{j},C_{j}\right)}^{2}]=E[{\left({\hat{a}}_{j}-{\hat{b}}_{j}\right)}^{2}{\left({\hat{a}}_{j}-{\hat{c}}_{j}\right)}^{2}]\\ =E[{\hat{a}}_{j}^{4}-2{\hat{a}}_{j}^{3}{\hat{c}}_{j}+{\hat{a}}_{j}^{2}{\hat{c}}_{j}^{2}-2{\hat{a}}_{j}^{3}{\hat{b}}_{j}+4{\hat{a}}_{j}^{2}{\hat{b}}_{j}{\hat{c}}_{j}-2{\hat{a}}_{j}{\hat{b}}_{j}{\hat{c}}_{j}^{2}+{\hat{a}}_{j}^{2}{\hat{b}}_{j}^{2}-2{\hat{a}}_{j}{\hat{b}}_{j}^{2}{\hat{c}}_{j}+{\hat{b}}_{j}^{2}{\hat{c}}_{j}^{2}]\\ \approx a_{j}^{4}+6\Phi_{2}(A_{j})a_{j}^{3}(1-a_{j})-2[a_{j}^{3}+3\Phi_{2}(A_{j})a_{j}^{2}(1-a_{j})]c_{j}\\ +[a_{j}^{2}+\Phi_{2}(A_{j})a_{j}(1-a_{j})][c_{j}^{2}+\Phi_{2}(C_{j})c_{j}(1-c_{j})]-2[a_{j}^{3}+3\Phi_{2}(A_{j})a_{j}^{2}(1-a_{j})]b_{j}\\ +4[a_{j}^{2}+\Phi_{2}(A_{j})a_{j}(1-a_{j})]b_{j}c_{j}-2a_{j}b_{j}[c_{j}^{2}+\Phi_{2}(C_{j})c_{j}(1-c_{j})]\\ +[a_{j}^{2}+\Phi_{2}(A_{j})a_{j}(1-a_{j})][b_{j}^{2}+\Phi_{2}(B_{j})b_{j}(1-b_{j})]-2a_{j}[b_{j}^{2}+\Phi_{2}(B_{j})b_{j}(1-b_{j})]c_{j}\\ +[b_{j}^{2}+\Phi_{2}(B_{j})b_{j}(1-b_{j})][c_{j}^{2}+\Phi_{2}(C_{j})c_{j}(1-c_{j})]\\ ={\left(a_{j}-b_{j}\right)}^{2}{\left(a_{j}-c_{j}\right)}^{2}+\Phi_{2}(A_{j})[6(a_{j}-b_{j})(a_{j}-c_{j})+{\left(b_{j}-c_{j}\right)}^{2}]a_{j}(1-a_{j})\\ +\Phi_{2}(B_{j}){\left(a_{j}-c_{j}\right)}^{2}b_{j}(1-b_{j})+\Phi_{2}(C_{j}){\left(a_{j}-b_{j}\right)}^{2}c_{j}(1-c_{j})\\ +\Phi_{2}(A_{j})\Phi_{2}(B_{j})a_{j}(1-a_{j})b_{j}(1-b_{j})+\Phi_{2}(A_{j})\Phi_{2}(C_{j})a_{j}(1-a_{j})c_{j}(1-c_{j})\\ +\Phi_{2}(B_{j})\Phi_{2}(C_{j})b_{j}(1-b_{j})c_{j}(1-c_{j})\\ =F_{3}{\left(A_{j};B_{j},C_{j}\right)}^{2}+6\Phi_{2}(A_{j})F_{3}(A_{j};B_{j},C_{j})G(A_{j})+\Phi_{2}(A_{j})F_{2}(B_{j},C_{j})G(A_{j})\\ +\Phi_{2}(B_{j})F_{2}(A_{j},C_{j})G(B_{j})+\Phi_{2}(C_{j})F_{2}(A_{j},B_{j})G(C_{j})+\Phi_{2}(A_{j})\Phi_{2}(B_{j})G(A_{j})G(B_{j})\\ +\Phi_{2}(A_{j})\Phi_{2}(C_{j})G(A_{j})G(C_{j})+\Phi_{2}(B_{j})\Phi_{2}(C_{j})G(B_{j})G(C_{j})\\ \approx F_{3}{\left(A_{j};B_{j},C_{j}\right)}^{2}+6\Phi_{2}(A_{j})F_{3}(A_{j};B_{j},C_{j})G(A_{j})+\Phi_{2}(A_{j})F_{2}(B_{j},C_{j})G(A_{j})\\ +\Phi_{2}(B_{j})F_{2}(A_{j},C_{j})G(B_{j})+\Phi_{2}(C_{j})F_{2}(A_{j},B_{j})G(C_{j}), \end{array}$$

where we used the fact that $\Phi_{2}(A_{j})\Phi_{2}(B_{j}),\;\Phi_{2}(A_{j})\Phi_{2}(C_{j})$, and $\Phi_{2}(B_{j})\Phi_{2}(C_{j})$ are negligible compared to $\Phi_{2}(A_{j}),\;\Phi_{2}(B_{j})$, and $\Phi_{2}(C_{j})$ as an approximation. Therefore, we have that

$$\begin{array}{l} Var[{\hat{F}}_{3}(A_{j};B_{j},C_{j})]=E[{\hat{F}}_{3}{\left(A_{j};B_{j},C_{j}\right)}^{2}]-E{\left[{\hat{F}}_{3}(A_{j};B_{j},C_{j})\right]}^{2}\\ \approx F_{3}{\left(A_{j};B_{j},C_{j}\right)}^{2}+6\Phi_{2}(A_{j})F_{3}(A_{j};B_{j},C_{j})G(A_{j})+\Phi_{2}(A_{j})F_{2}(B_{j},C_{j})G(A_{j})\\ +\Phi_{2}(B_{j})F_{2}(A_{j},C_{j})G(B_{j})+\Phi_{2}(C_{j})F_{2}(A_{j},B_{j})G(C_{j})\\ -[F_{3}{\left(A_{j};B_{j},C_{j}\right)}^{2}+2\Phi_{2}(A_{j})F_{3}(A_{j};B_{j},C_{j})G(A_{j})]\\ =4\Phi_{2}(A_{j})F_{3}(A_{j};B_{j},C_{j})G(A_{j})+\Phi_{2}(A_{j})F_{2}(B_{j},C_{j})G(A_{j})\\ +\Phi_{2}(B_{j})F_{2}(A_{j},C_{j})G(B_{j})+\Phi_{2}(C_{j})F_{2}(A_{j},B_{j})G(C_{j}), \end{array}$$

which gives

$$\begin{array}{l} Var[{\hat{F}}_{3}(A;B,C)]=\mathrm{Var}[\frac{1}{J}\sum_{j=1}^{J}{\hat{F}}_{3}(A_{j};B_{j},C_{j})]\\ =\frac{1}{J^{2}}\sum_{j=1}^{J}Var[{\hat{F}}_{3}(A_{j};B_{j},C_{j})]\\ \approx \frac{4}{J^{2}}\sum_{j=1}^{J}\Phi_{2}(A_{j})F_{3}(A_{j};B_{j},C_{j})G(A_{j})+\frac{1}{J^{2}}\sum_{j=1}^{J}\Phi_{2}(A_{j})F_{2}(B_{j},C_{j})G(A_{j})\\ +\frac{1}{J^{2}}\sum_{j=1}^{J}\Phi_{2}(B_{j})F_{2}(A_{j},C_{j})G(B_{j})+\frac{1}{J^{2}}\sum_{j=1}^{J}\Phi_{2}(C_{j})F_{2}(A_{j},B_{j})G(C_{j}).\;□ \end{array}$$

**Lemma 24.** Consider *J* independent polymorphic loci in populations *A*, *B*, and *C* with respective parametric reference allele frequencies $a_{j},b_{j},c_{j}\in (0,1)$, and suppose we take a random sample of $N(P_{j})$ individuals at locus *j* in population P∈{A,B,C}, some of which may be related or inbred. Moreover, assume that no individual is related to more than one other individual, which makes the terms $\Phi_{3}(P_{j}),\;\Phi_{4}(P_{j}),\;\Phi_{2,2}(P_{j})$, and $\Phi_{2}{\left(P_{j}\right)}^{2}$ negligible to $\Phi_{2}(P_{j})$. Based on this simplifying assumption, the estimators ${\hat{F}}_{3}(A;B,C)$ and $\hat{g}(A)$ have an approximate covariance

$$\mathrm{Cov}[{\hat{F}}_{3}(A;B,C),\hat{G}(A)]\approx \frac{1}{J^{2}}\sum_{j=1}^{J}\Phi_{2}(A_{j})G(A_{j})[2G(A_{j})-2F_{3}(A_{j};B_{j},C_{j})-b_{j}(1-c_{j})-(1-b_{j})c_{j}].$$

*Proof*. From the proofs of Lemma 1 and Proposition 6, we have

$$E[\hat{G}(A_{j})]=[1-\Phi_{2}(A_{j})]G(A_{j})$$

and

$$E[{\hat{F}}_{3}(A_{j};B_{j},C_{j})]=F_{3}(A_{j};B_{j},C_{j})+\Phi_{2}(A_{j})G(A_{j}),$$

yielding

$$\begin{array}{l} E[{\hat{F}}_{3}(A_{j};B_{j},C_{j})]E[\hat{G}(A_{j})]=[F_{3}(A_{j};B_{j},C_{j})+\Phi_{2}(A_{j})G(A_{j})][1-\Phi_{2}(A_{j})]G(A_{j})\\ =F_{3}(A_{j};B_{j},C_{j})G(A_{j})+\Phi_{2}(A_{j})G{\left(A_{j}\right)}^{2}-\Phi_{2}(A_{j})F_{3}(A_{j};B_{j},C_{j})G(A_{j})\\ -\Phi_{2}{\left(A_{j}\right)}^{2}G{\left(A_{j}\right)}^{2}\\ \approx F_{3}(A_{j};B_{j},C_{j})G(A_{j})+\Phi_{2}(A_{j})G{\left(A_{j}\right)}^{2}-\Phi_{2}(A_{j})F_{3}(A_{j};B_{j},C_{j})G(A_{j}), \end{array}$$

where we used the fact that $\Phi_{2}{\left(A_{j}\right)}^{2}$ is negligible compared to $\Phi_{2}(A_{j})$ as an approximation. We also calculate

$$\begin{array}{l} E[{\hat{F}}_{3}(A_{j};B_{j},C_{j})\hat{G}(A_{j})]=E[({\hat{a}}_{j}-{\hat{b}}_{j})({\hat{a}}_{j}-{\hat{c}}_{j}){\hat{a}}_{j}(1-{\hat{a}}_{j})]\\ =E[({\hat{a}}_{j}^{2}-{\hat{a}}_{j}{\hat{b}}_{j}-{\hat{a}}_{j}{\hat{c}}_{j}+{\hat{b}}_{j}{\hat{c}}_{j})({\hat{a}}_{j}-{\hat{a}}_{j}^{2})]\\ =E[{\hat{a}}_{j}^{3}]-E[{\hat{a}}_{j}^{2}]E[{\hat{b}}_{j}]-E[{\hat{a}}_{j}^{2}]E[{\hat{c}}_{j}]+E[{\hat{a}}_{j}]E[{\hat{b}}_{j}]E[{\hat{c}}_{j}]-E[{\hat{a}}_{j}^{4}]+E[{\hat{a}}_{j}^{3}]E[{\hat{b}}_{j}]\\ +E[{\hat{a}}_{j}^{3}]E[{\hat{c}}_{j}]-E[{\hat{a}}_{j}^{2}]E[{\hat{b}}_{j}]E[{\hat{c}}_{j}]\\ \approx a_{j}^{3}+3\Phi_{2}(A_{j})a_{j}^{2}(1-a_{j})-[a_{j}^{2}+\Phi_{2}(A_{j})a_{j}(1-a_{j})]b_{j}\\ -[a_{j}^{2}+\Phi_{2}(A_{j})a_{j}(1-a_{j})]c_{j}+a_{j}b_{j}c_{j}-[a_{j}^{4}+6\Phi_{2}(A_{j})a_{j}^{3}(1-a_{j})]\\ +[a_{j}^{3}+3\Phi_{2}(A_{j})a_{j}^{2}(1-a_{j})]b_{j}+[a_{j}^{3}+3\Phi_{2}(A_{j})a_{j}^{2}(1-a_{j})]c_{j}\\ -[a_{j}^{2}+\Phi_{2}(A_{j})a_{j}(1-a_{j})]b_{j}c_{j}. \end{array}$$

Recognizing that $G(A_{j})=a_{j}(1-a_{j})$ and $F_{3}(A_{j};B_{j},C_{j})=a_{j}^{2}-a_{j}b_{j}-a_{j}c_{j}+b_{j}c_{j}$, we have

$$\begin{array}{l} E[{\hat{F}}_{3}(A_{j};B_{j},C_{j})\hat{G}(B_{j})]\approx a_{j}^{3}-a_{j}^{2}b_{j}-a_{j}^{2}c_{j}+a_{j}b_{j}c_{j}-a_{j}^{4}+a_{j}^{3}b_{j}+a_{j}^{3}c_{j}-a_{j}^{2}b_{j}c_{j}\\ +\Phi_{2}(A_{j})[3a_{j}-b_{j}-c_{j}-6a_{j}^{2}+3a_{j}b_{j}+3a_{j}c_{j}-b_{j}c_{j}]G(A_{j})\\ =(a_{j}^{2}-a_{j}b_{j}-a_{j}c_{j}+b_{j}c_{j})(a_{j}-a_{j}^{2})\\ +\Phi_{2}(A_{j})[3(a_{j}-a_{j}^{2})-3(a_{j}^{2}-a_{j}b_{j}-a_{j}c_{j}+b_{j}c_{j})-b_{j}(1-c_{j})\\ -(1-b_{j})c_{j}]G(A_{j})\\ =F_{3}(A_{j};B_{j},C_{j})G(A_{j})\\ +\Phi_{2}(A_{j})[3G(A_{j})-3F_{3}(A_{j};B_{j},C_{j})-b_{j}(1-c_{j})-(1-b_{j})c_{j}]G(A_{j})\\ =F_{3}(A_{j};B_{j},C_{j})G(A_{j})+3\Phi_{2}(A_{j})G{\left(A_{j}\right)}^{2}-3\Phi_{2}(A_{j})F_{3}(A_{j};B_{j},C_{j})G(A_{j})\\ -\Phi_{2}(A_{j})G(A_{j})b_{j}(1-c_{j})-\Phi_{2}(A_{j})G(A_{j})(1-b_{j})c_{j}\\ =[1-3\Phi_{2}(A_{j})]F_{3}(A_{j};B_{j},C_{j})G(A_{j})+3\Phi_{2}(A_{j})G{\left(A_{j}\right)}^{2}\\ -\Phi_{2}(A_{j})G(A_{j})b_{j}(1-c_{j})-\Phi_{2}(A_{j})G(A_{j})(1-b_{j})c_{j}. \end{array}$$

Therefore, we have that

$$\begin{array}{l} Cov[{\hat{F}}_{3}(A_{j};B_{j},C_{j}),\hat{G}(A_{j})]=E[{\hat{F}}_{3}(A_{j};B_{j},C_{j})\hat{G}(A_{j})]-E[{\hat{F}}_{3}(A_{j};B_{j},C_{j})]E[\hat{G}(A_{j})]\\ \approx [1-3\Phi_{2}(A_{j})]F_{3}(A_{j};B_{j},C_{j})G(A_{j})+3\Phi_{2}(A_{j})G{\left(A_{j}\right)}^{2}\\ -\Phi_{2}(A_{j})G(A_{j})b_{j}(1-c_{j})-\Phi_{2}(A_{j})G(A_{j})(1-b_{j})c_{j}\\ -[F_{3}(A_{j};B_{j},C_{j})G(A_{j})+\Phi_{2}(A_{j})G{\left(A_{j}\right)}^{2}\\ -\Phi_{2}(A_{j})F_{3}(A_{j};B_{j},C_{j})G(A_{j})]\\ =2\Phi_{2}(A_{j})G{\left(A_{j}\right)}^{2}-2\Phi_{2}(A_{j})F_{3}(A_{j};B_{j},C_{j})G(A_{j})\\ -\Phi_{2}(A_{j})G(A_{j})b_{j}(1-c_{j})-\Phi_{2}(A_{j})G(A_{j})(1-b_{j})c_{j}\\ =\Phi_{2}(A_{j})G(A_{j})[2G(A_{j})-2F_{3}(A_{j};B_{j},C_{j})-b_{j}(1-c_{j})-(1-b_{j})c_{j}], \end{array}$$

which gives

$$\begin{array}{l} Cov[{\hat{F}}_{3}(A;B,C),\hat{G}(A)]=\mathrm{Cov}[\frac{1}{J}\sum_{j=1}^{J}{\hat{F}}_{3}(A_{j};B_{j},C_{j}),\frac{1}{J}\sum_{j=1}^{J}\hat{G}(A_{j})]\\ =\frac{1}{J^{2}}\sum_{j=1}^{J}Cov[{\hat{F}}_{3}(A_{j};B_{j},C_{j}),\hat{G}(A_{j})]\\ \approx \frac{1}{J^{2}}\sum_{j=1}^{J}\Phi_{2}(A_{j})G(A_{j})[2G(A_{j})-2F_{3}(A_{j};B_{j},C_{j})-b_{j}(1-c_{j})\\ -(1-b_{j})c_{j}].\;□ \end{array}$$

**Proposition 25.** Consider *J* independent polymorphic loci in populations *A*, *B*, and *C* with respective parametric reference allele frequencies $a_{j},b_{j},c_{j}\in (0,1)$, and suppose we take a random sample of $N(P_{j})$ individuals at locus *j* in population P∈{A,B,C}, some of which may be related or inbred. Moreover, assume that no individual is related to more than one other individual, which makes the terms $\Phi_{3}(P_{j}),\;\Phi_{4}(P_{j}),\;\Phi_{2,2}(P_{j}),\;\Phi_{2}{\left(P_{j}\right)}^{2},\;\Phi_{2}(A_{j})\Phi_{2}(B_{j}),\;\Phi_{2}(A_{j})\Phi_{2}(C_{j})$, and $\Phi_{2}(B_{j})\Phi_{2}(C_{j})$ negligible to $\Phi_{2}(P_{j})$. Based on this simplifying assumption, the unbiased estimator ${\tilde{F}}_{3}(A;B,C)$ has approximate variance

$$\begin{array}{l} Var[{\tilde{F}}_{3}(A;B,C)]\approx \frac{4}{J^{2}}\sum_{j=1}^{J}\Phi_{2}(A_{j})F_{3}(A_{j};B_{j},C_{j})G(A_{j})+\frac{1}{J^{2}}\sum_{j=1}^{J}\Phi_{2}(A_{j})F_{2}(B_{j},C_{j})G(A_{j})\\ +\frac{1}{J^{2}}\sum_{j=1}^{J}\Phi_{2}(B_{j})F_{2}(A_{j},C_{j})G(B_{j})+\frac{1}{J^{2}}\sum_{j=1}^{J}\Phi_{2}(C_{j})F_{2}(A_{j},B_{j})G(C_{j}). \end{array}$$

*Proof*. Recall that

$${\tilde{F}}_{3}(A_{j};B_{j},C_{j})={\hat{F}}_{3}(A_{j};B_{j},C_{j})-\Phi_{2}(A_{j})\tilde{G}(A_{j}),$$

where ${\tilde{F}}_{3}(A_{j};B_{j},C_{j})$ is an unbiased estimator for $F_{3}(A_{j};B_{j},C_{j})$ and $\tilde{G}(A_{j})=\hat{G}(A_{j})/[1-\Phi_{2}(A_{j})]$ is an unbiased estimator of $G(A_{j})$ at locus j∈{1,2,…,J}. Also, from the proof of Proposition 6, we have

$$E[{\hat{F}}_{3}(A_{j};B_{j},C_{j})]=F_{3}(A_{j};B_{j},C_{j})+\Phi_{2}(A_{j})G(A_{j}).$$

Therefore, we have that

$$\begin{array}{l} Var[{\tilde{F}}_{3}(A_{j};B_{j};C_{j})]=\mathrm{Var}[{\hat{F}}_{3}(A_{j};B_{j},C_{j})-\Phi_{2}(A_{j})\tilde{G}(A_{j})]\\ =\mathrm{Var}[{\hat{F}}_{3}(A_{j};B_{j},C_{j})]+\Phi_{2}{\left(A_{j}\right)}^{2}Var[\tilde{G}(A_{j})]\\ -2\Phi_{2}(A_{j})\mathrm{Cov}[{\hat{F}}_{3}(A_{j};B_{j},C_{j}),\tilde{G}(A_{j})]\\ \approx \mathrm{Var}[{\hat{F}}_{3}(A_{j};B_{j},C_{j})]-\frac{2\Phi_{2}(A_{j})}{1-\Phi_{2}(A_{j})}Cov[{\hat{F}}_{3}(A_{j};B_{j},C_{j}),\hat{G}(A_{j})], \end{array}$$

where we used the fact that $\Phi_{2}{\left(A_{j}\right)}^{2}$ is negligible compared to $\Phi_{2}(A_{j})$ as an approximation. Moreover, because $\tilde{G}(A_{j})=\hat{G}(A_{j})/[1-\Phi_{2}(A_{j})]$, we have

$$\begin{array}{l} Var[{\tilde{F}}_{3}(A_{j};B_{j};C_{j})]\approx \mathrm{Var}[{\hat{F}}_{3}(A_{j};B_{j},C_{j})]-\frac{2\Phi_{2}(A_{j})}{1-\Phi_{2}(A_{j})}Cov[{\hat{F}}_{3}(A_{j};B_{j},C_{j}),\hat{g}(A_{j})]\\ =\mathrm{Var}[{\hat{F}}_{3}(A_{j};B_{j},C_{j})]\\ -\frac{2\Phi_{2}{\left(A_{j}\right)}^{2}}{1-\Phi_{2}(A_{j})}G(A_{j})[2G(A_{j})-2F_{3}(A_{j};B_{j},C_{j})-b_{j}(1-c_{j})-(1-b_{j})c_{j}]. \end{array}$$

Recalling the assumption that $\Phi_{2}{\left(A_{j}\right)}^{2}$ is negligible compared to $\Phi_{2}(A_{j})$, we have

$$\mathrm{Var}[{\tilde{F}}_{3}(A_{j};B_{j},C_{j})]\approx \mathrm{Var}[{\hat{F}}_{3}(A_{j};B_{j},C_{j})].\;□$$

**Proposition 26.** Consider *J* independent polymorphic loci in populations *A*, *B*, and *C* with respective parametric reference allele frequencies $a_{j},b_{j},c_{j}\in (0,1)$, and suppose we take a random sample of $N(P_{j})$ individuals at locus *j* in population P∈{A,B,C}, some of which may be related or inbred. Moreover, assume that no individual is related to more than one other individual, which makes the terms $\Phi_{3}(P_{j}),\;\Phi_{4}(P_{j}),\;\Phi_{2,2}(P_{j}),\;\Phi_{2}{\left(P_{j}\right)}^{2},\;\Phi_{2}(A_{j})\Phi_{2}(B_{j}),\;\Phi_{2}(A_{j})\Phi_{2}(C_{j}),\;\Phi_{2}(B_{j})\Phi_{2}(C_{j})$ and any other terms of similar order negligible to $\Phi_{2}(P_{j})$. Based on this simplifying assumption, the estimator ${\overset{⌣}{F}}_{3}(A;B,C)$ has approximate variance

$$\begin{array}{l} Var[{\overset{⌣}{F}}_{3}(A;B,C)]\approx \frac{4}{J^{2}}\sum_{j=1}^{J}\Phi_{2}(A_{j})F_{3}(A_{j};B_{j},C_{j})G(A_{j})+\frac{1}{J^{2}}\sum_{j=1}^{J}\Phi_{2}(A_{j})F_{2}(B_{j},C_{j})G(A_{j})\\ +\frac{1}{J^{2}}\sum_{j=1}^{J}\Phi_{2}(B_{j})F_{2}(A_{j},C_{j})G(B_{j})+\frac{1}{J^{2}}\sum_{j=1}^{J}\Phi_{2}(C_{j})F_{2}(A_{j},B_{j})G(C_{j}). \end{array}$$

*Proof*. Recall that

$${\overset{⌣}{F}}_{3}(A_{j};B_{j},C_{j})={\hat{F}}_{3}(A_{j};B_{j},C_{j})-\frac{1}{\sum_{k=1}^{N(A_{j})}m_{k}-1}\hat{G}(A_{j}),$$

where ${\overset{⌣}{F}}_{3}(A_{j};B_{j},C_{j})$ and ${\hat{F}}_{3}(A_{j};B_{j},C_{j})$ are estimators for $F_{3}(A_{j};B_{j},C_{j})$ and $\hat{G}(A_{j})$ is an estimator of $G(A_{j})$ at locus j∈{1,2,…,J}. Also, from the proof of Proposition 6, we have

$$E[{\hat{F}}_{3}(A_{j};B_{j},C_{j})]=F_{3}(A_{j};B_{j},C_{j})+\Phi_{2}(A_{j})G(A_{j}).$$

Therefore, we have that

$$\begin{array}{l} Var[{\overset{⌣}{F}}_{3}(A_{j};B_{j};C_{j})]=\mathrm{Var}[{\hat{F}}_{3}(A_{j};B_{j},C_{j})-\frac{1}{\sum_{k=1}^{N(A_{j})}m_{k}-1}\hat{G}(A_{j})]\\ =\mathrm{Var}[{\hat{F}}_{3}(A_{j};B_{j},C_{j})]+{\left[\frac{1}{\sum_{k=1}^{N(A_{j})}m_{k}-1}\right]}^{2}Var[\hat{G}(A_{j})]\\ -2[\frac{1}{\sum_{k=1}^{N(A_{j})}m_{k}-1}]\mathrm{Cov}[{\hat{F}}_{3}(A_{j};B_{j},C_{j}),\hat{G}(A_{j})]\\ \approx \mathrm{Var}[{\hat{F}}_{3}(A_{j};B_{j},C_{j})]-[\frac{2}{\sum_{k=1}^{N(A_{j})}m_{k}-1}]\mathrm{Cov}[{\hat{F}}_{3}(A_{j};B_{j},C_{j}),\hat{G}(A_{j})], \end{array}$$

where we used the fact that $\Phi_{2}{\left(A_{j}\right)}^{2}$ is negligible compared to $\Phi_{2}(A_{j})$ as an approximation (and hence $1/[\sum_{k=1}^{N(A_{j})}m_{k}-1]$ assuming $N(A_{j})$ is large enough). Moreover, because

$$\mathrm{Cov}[{\hat{F}}_{3}(A_{j};B_{j},C_{j}),\hat{G}(A_{j})]=\Phi_{2}(A_{j})G(A_{j})[2G(A_{j})-2F_{3}(A_{j};B_{j},C_{j})-b_{j}(1-c_{j})-(1-b_{j})c_{j}]$$

we have

$$[\frac{2}{\sum_{k=1}^{N(A_{j})}m_{k}-1}]\mathrm{Cov}[{\hat{F}}_{3}(A_{j};B_{j},C_{j}),\hat{G}(A_{j})]\approx 0$$

by recalling the assumption that $\Phi_{2}{\left(A_{j}\right)}^{2}$ or any other term of similar magnitude (such as the $\Phi_{2}(A_{j})/[\sum_{k=1}^{N(A_{j})}m_{k}-1]$ term) are negligible compared to $\Phi_{2}(A_{j})$. Because of this, we have

$$\mathrm{Var}[{\overset{⌣}{F}}_{3}(A_{j};B_{j},C_{j})]\approx \mathrm{Var}[{\hat{F}}_{3}(A_{j};B_{j},C_{j})]$$

and it follows that

$$\mathrm{Var}[{\overset{⌣}{F}}_{3}(A;B,C)]\approx \mathrm{Var}[{\hat{F}}_{3}(A;B,C)].\;□$$

**Proposition 27.** Consider *J* independent polymorphic loci in populations *A*, *B*, *C*, and *D* with respective parametric reference allele frequencies $a_{j},b_{j},c_{j},d_{j}\in (0,1)$, and suppose we take a random sample of $N(P_{j})$ individuals at locus *j* in population P∈{A,B,C,D}, some of which may be related or inbred. Moreover, assume that no individual is related to more than one other individual, which makes the terms $\Phi_{3}(P_{j}),\;\Phi_{4}(P_{j}),\;\Phi_{2,2}(P_{j}),\;\Phi_{2}{\left(P_{j}\right)}^{2},\;\Phi_{2}(A_{j})\Phi_{2}(C_{j}),\;\Phi_{2}(A_{j})\Phi_{2}(D_{j}),\;\Phi_{2}(B_{j})\Phi_{2}(C_{j})$, and $\Phi_{2}(B_{j})\Phi_{2}(D_{j})$ negligible to $\Phi_{2}(P_{j})$. Based on this simplifying The unbiased estimator ${\hat{F}}_{4}(A,B;C,D)$ has approximate variance

$$\begin{array}{l} Var[{\hat{F}}_{4}(A,B;C,D)]\approx \frac{1}{J^{2}}\sum_{j=1}^{J}[\Phi_{2}(C_{j})G(C_{j})+\Phi_{2}(D_{j})G(D_{j})]F_{2}(A_{j},B_{j})\\ +\frac{1}{J^{2}}\sum_{j=1}^{J}[\Phi_{2}(A_{j})G(A_{j})+\Phi_{2}(B_{j})G(B_{j})]F_{2}(C_{j},D_{j}). \end{array}$$

*Proof*. From the proofs of Propositions 3 and 12, we have

$$E[{\hat{F}}_{2}(A_{j},B_{j})]=F_{2}(A_{j},B_{j})+\Phi_{2}(A_{j})G(A_{j})+\Phi_{2}(B_{j})G(B_{j})$$

and

$$E[{\hat{F}}_{4}(A_{j},B_{j};C_{j},D_{j})]=F_{4}(A_{j},B_{j};C_{j},D_{j}).$$

We calculate

$$\begin{array}{l} E[{\hat{F}}_{4}{\left(A_{j},B_{j};C_{j},D_{j}\right)}^{2}]=E[{\left({\hat{a}}_{j}-{\hat{b}}_{j}\right)}^{2}{\left({\hat{c}}_{j}-{\hat{d}}_{j}\right)}^{2}]\\ =E[{\hat{F}}_{2}(A_{j},B_{j}){\hat{F}}_{2}(C_{j},D_{j})]\\ =E[{\hat{F}}_{2}(A_{j},B_{j})]E[{\hat{F}}_{2}(C_{j},D_{j})]\\ =[F_{2}(A_{j},B_{j})+\Phi_{2}(A_{j})G(A_{j})+\Phi_{2}(B_{j})G(B_{j})]\\ \times [F_{2}(C_{j},D_{j})+\Phi_{2}(C_{j})G(C_{j})+\Phi_{2}(D_{j})G(D_{j})]\\ =F_{4}{\left(A_{j},B_{j};C_{j},D_{j}\right)}^{2}+F_{2}(A_{j},B_{j})[\Phi_{2}(C_{j})G(C_{j})+\Phi_{2}(D_{j})G(D_{j})]\\ +F_{2}(C_{j},D_{j})[\Phi_{2}(A_{j})G(A_{j})+\Phi_{2}(B_{j})G(B_{j})]\\ +[\Phi_{2}(A_{j})G(A_{j})+\Phi_{2}(B_{j})G(B_{j})][\Phi_{2}(C_{j})G(C_{j})+\Phi_{2}(D_{j})G(D_{j})], \end{array}$$

where we use the identity that

$$\begin{array}{l} F_{4}{\left(A_{j},B_{j};C_{j},D_{j}\right)}^{2}={\left(a_{j}-b_{j}\right)}^{2}{\left(c_{j}-d_{j}\right)}^{2}\\ =F_{2}(A_{j},B_{j})F_{2}(C_{j},D_{j}). \end{array}$$

Therefore, we have that

$$\begin{array}{l} Var[{\hat{F}}_{4}(A_{j},B_{j};C_{j},D_{j})]=E[{\hat{F}}_{4}{\left(A_{j},B_{j};C_{j},D_{j}\right)}^{2}]-E{\left[{\hat{F}}_{4}(A_{j},B_{j};C_{j},D_{j})\right]}^{2}\\ =F_{4}{\left(A_{j},B_{j};C_{j},D_{j}\right)}^{2}+F_{2}(A_{j},B_{j})[\Phi_{2}(C_{j})G(C_{j})+\Phi_{2}(D_{j})G(D_{j})]\\ +F_{2}(C_{j},D_{j})[\Phi_{2}(A_{j})G(A_{j})+\Phi_{2}(B_{j})G(B_{j})]\\ +[\Phi_{2}(A_{j})G(A_{j})+\Phi_{2}(B_{j})G(B_{j})][\Phi_{2}(C_{j})G(C_{j})+\Phi_{2}(D_{j})G(D_{j})]\\ -F_{4}{\left(A_{j},B_{j};C_{j},D_{j}\right)}^{2}\\ =F_{2}(A_{j},B_{j})[\Phi_{2}(C_{j})G(C_{j})+\Phi_{2}(D_{j})G(D_{j})]\\ +F_{2}(C_{j},D_{j})[\Phi_{2}(A_{j})G(A_{j})+\Phi_{2}(B_{j})G(B_{j})]\\ +[\Phi_{2}(A_{j})G(A_{j})+\Phi_{2}(B_{j})G(B_{j})][\Phi_{2}(C_{j})G(C_{j})+\Phi_{2}(D_{j})G(D_{j})]\\ \approx F_{2}(A_{j},B_{j})[\Phi_{2}(C_{j})G(C_{j})+\Phi_{2}(D_{j})G(D_{j})]\\ +F_{2}(C_{j},D_{j})[\Phi_{2}(A_{j})G(A_{j})+\Phi_{2}(B_{j})G(B_{j})], \end{array}$$

where we used the fact that $\Phi_{2}(A_{j})\Phi_{2}(C_{j}),\;\Phi_{2}(A_{j})\Phi_{2}(D_{j}),\;\Phi_{2}(B_{j})\Phi_{2}(C_{j})$, and $\Phi_{2}(C_{j})\Phi_{2}(D_{j})$ are negligible compared to $\Phi_{2}(A_{j}),\;\Phi_{2}(B_{j}),\;\Phi_{2}(C_{j})$ and $\Phi_{2}(D_{j})$ as an approximation. It follows that

$$\begin{array}{l} Var[{\hat{F}}_{4}(A,B;C,D)]=\mathrm{Var}[\frac{1}{J}\sum_{j=1}^{J}{\hat{F}}_{4}(A_{j},B_{j};C_{j},D_{j})]\\ =\frac{1}{J^{2}}\sum_{j=1}^{J}Var[{\hat{F}}_{4}(A_{j},B_{j};C_{j},D_{j})]\\ \approx \frac{1}{J^{2}}\sum_{j=1}^{J}[\Phi_{2}(C_{j})G(C_{j})+\Phi_{2}(D_{j})G(D_{j})]F_{2}(A_{j},B_{j})\\ +\frac{1}{J^{2}}\sum_{j=1}^{J}[\Phi_{2}(A_{j})G(A_{j})+\Phi_{2}(B_{j})G(B_{j})]F_{2}(C_{j},D_{j}).\;□ \end{array}$$

Following Wolter (2007), we have that an approximation to the variance of the ratio estimator *X*/*Y* is

$$\mathrm{Var}[\frac{X}{Y}]\approx \frac{E{\left[X\right]}^{2}}{E{\left[Y\right]}^{2}}[\frac{\mathrm{Var}[X]}{E{\left[X\right]}^{2}}+\frac{\mathrm{Var}[Y]}{E{\left[Y\right]}^{2}}-2\frac{\mathrm{Cov}[X,Y]}{E[X]E[Y]}]$$

**Proposition 28.** Consider *J* polymorphic loci in populations *A*, *B*, and *C* with respective parametric reference allele frequencies $a_{j},b_{j},c_{j}\in (0,1)$, and suppose we take a random sample of $N(P_{j})$ individuals at locus *j* in population P∈{A,B,C}, some of which may be related or inbred. Moreover, assume that no individual is related to more than one other individual, which makes the terms $\Phi_{3}(P_{j}),\;\Phi_{4}(P_{j}),\;\Phi_{2,2}(P_{j}),\;\Phi_{2}{\left(P_{j}\right)}^{2},\;\Phi_{2}(A_{j})\Phi_{2}(B_{j}),\;\Phi_{2}(A_{j})\Phi_{2}(C_{j})$, and $\Phi_{2}(B_{j})\Phi_{2}(C_{j})$ negligible to $\Phi_{2}(P_{j})$. Based on this simplifying assumption, the ratio estimator ${\hat{F}}_{3}(A;B,C|A)$ has approximate variance

$$\mathrm{Var}[{\hat{F}}_{3}(A;B,C|A)]\approx \frac{E{\left[{\hat{F}}_{3}(A;B,C)\right]}^{2}}{4E{\left[\hat{G}(A)\right]}^{2}}[\frac{\mathrm{Var}[{\hat{F}}_{3}(A;B,C)]}{E{\left[{\hat{F}}_{3}(A;B,C)\right]}^{2}}+\frac{\mathrm{Var}[\hat{G}(A)]}{E{\left[\hat{G}(A)\right]}^{2}}-2\frac{\mathrm{Cov}[{\hat{F}}_{3}(A;B,C),\hat{G}(A)]}{E[{\hat{F}}_{3}(A;B,C)]E[\hat{G}(A)]}],$$

where the expectations are

$$\begin{array}{l} E[{\hat{F}}_{3}(A;B,C)]=F_{3}(A;B,C)+\frac{1}{J}\sum_{j=1}^{J}\Phi_{2}(A_{j})G(A_{j})\\ E[\hat{G}(A)]=G(A)-\frac{1}{J}\sum_{j=1}^{J}\Phi_{2}(A_{j})G(A_{j}) \end{array}$$

the variances are

$$\begin{array}{l} Var[{\hat{F}}_{3}(A;B,C)]\approx \frac{4}{J^{2}}\sum_{j=1}^{J}\Phi_{2}(A_{j})F_{3}(A_{j};B_{j},C_{j})G(A_{j})+\frac{1}{J^{2}}\sum_{j=1}^{J}\Phi_{2}(A_{j})F_{2}(B_{j},C_{j})G(A_{j})\\ +\frac{1}{J^{2}}\sum_{j=1}^{J}\Phi_{2}(B_{j})F_{2}(A_{j},C_{j})G(B_{j})+\frac{1}{J^{2}}\sum_{j=1}^{J}\Phi_{2}(C_{j})F_{2}(A_{j},B_{j})G(C_{j})\\ Var[\hat{G}(A)]\approx \frac{1}{J^{2}}\sum_{j=1}^{J}\Phi_{2}(A_{j})G(A_{j})-\frac{4}{J^{2}}\sum_{j=1}^{J}\Phi_{2}(A_{j})G{\left(A_{j}\right)}^{2} \end{array}$$

and the covariance is

$$\mathrm{Cov}[{\hat{F}}_{3}(A;B,C),\hat{G}(A)]\approx \frac{1}{J^{2}}\sum_{j=1}^{J}\Phi_{2}(A_{j})G(A_{j})[2G(A_{j})-2F_{3}(A_{j};B_{j},C_{j})-b_{j}(1-c_{j})-(1-b_{j})c_{j}].$$

*Proof*. Recall that

$${\hat{F}}_{3}(A;B,C,|A)=\frac{{\hat{F}}_{3}(A;B,C)}{2\hat{G}(A)}.$$

Assuming that $X={\hat{F}}_{3}(A;B,C)$ and $Y=2\hat{G}(A)$, following the approximation in Wolter (2007) we have

$$\mathrm{Var}[{\hat{F}}_{3}(A;B,C|A)]\approx \frac{E{\left[{\hat{F}}_{3}(A;B,C)\right]}^{2}}{4E{\left[\hat{G}(A)\right]}^{2}}[\frac{\mathrm{Var}[{\hat{F}}_{3}(A;B,C)]}{E{\left[{\hat{F}}_{3}(A;B,C)\right]}^{2}}+\frac{\mathrm{Var}[\hat{G}(A)]}{E{\left[\hat{G}(A)\right]}^{2}}-2\frac{\mathrm{Cov}[{\hat{F}}_{3}(A;B,C),\hat{G}(A)]}{E[{\hat{F}}_{3}(A;B,C)]E[\hat{G}(A)]}],$$

where $E[{\hat{F}}_{3}(A;B,C)]$ is given in Proposition 6, $E[\hat{G}(A)]$ in Lemma 1, $\mathrm{Var}[{\hat{F}}_{3}(A;B,C)]$ in Proposition 23, $\mathrm{Var}[\hat{G}(A)]$ in Lemma 18, and $\mathrm{Cov}[{\hat{F}}_{3}(A;B,C),\hat{G}(A)]$ in Lemma 24. □

**Lemma 29.** Consider *J* independent polymorphic loci in populations *A*, *B*, and *C* with respective parametric reference allele frequencies $a_{j},b_{j},c_{j}\in (0,1)$, and suppose we take a random sample of $N(P_{j})$ individuals at locus *j* in population P∈{A,B,C}, some of which may be related or inbred. Moreover, assume that no individual is related to more than one other individual, which makes the terms $\Phi_{3}(P_{j}),\;\Phi_{4}(P_{j}),\;\Phi_{2,2}(P_{j})$, and $\Phi_{2}{\left(P_{j}\right)}^{2}$ negligible to $\Phi_{2}(P_{j})$. Based on this simplifying assumption, the unbiased estimators ${\tilde{F}}_{3}(A;B,C)$ and $\tilde{G}(A)$ have an approximate covariance

$$\mathrm{Cov}[{\tilde{F}}_{3}(A;B,C),\tilde{G}(A)]\approx \frac{1}{J^{2}}\sum_{j=1}^{J}\frac{\Phi_{2}(A_{j})}{1-\Phi_{2}(A_{j})}G(A_{j})[2G(A_{j})-2F_{3}(A_{j};B_{j},C_{j})-b_{j}(1-c_{j})-(1-b_{j})c_{j}].$$

*Proof*. Recall that

$${\tilde{F}}_{3}(A_{j};B_{j},C_{j})={\hat{F}}_{3}(A_{j};B_{j},C_{j})-\Phi_{2}(A_{j})\tilde{G}(A_{j}),$$

where $\tilde{G}(A_{j})=\hat{G}(A_{j})/[1-\Phi_{2}(A_{j})]$. It follows that

$$\begin{array}{l} Cov[{\tilde{F}}_{3}(A_{j};B_{j},C_{j}),\tilde{G}(A_{j})]=\mathrm{Cov}[{\hat{F}}_{3}(A_{j};B_{j},C_{j})-\Phi_{2}(A_{j})\tilde{G}(A_{j}),\tilde{G}(A_{j})]\\ =\mathrm{Cov}[{\hat{F}}_{3}(A_{j};B_{j},C_{j}),\tilde{G}(A_{j})]-\Phi_{2}(A_{j})\mathrm{Var}[\tilde{G}(A_{j})]\\ =\frac{1}{1-\Phi_{2}(A_{j})}Cov[{\hat{F}}_{3}(A_{j};B_{j},C_{j}),\hat{G}(A_{j})]-\frac{\Phi_{2}(A_{j})}{{\left[1-\Phi_{2}(A_{j})\right]}^{2}}Var[\hat{G}(A_{j})]\\ \approx \frac{1}{1-\Phi_{2}(A_{j})}Cov[{\hat{F}}_{3}(A_{j};B_{j},C_{j}),\hat{G}(A_{j})]-\frac{\Phi_{2}(A_{j})}{1-2\Phi_{2}(A_{j})}Var[\hat{G}(A_{j})], \end{array}$$

where we used the fact that $\Phi_{2}{\left(A_{j}\right)}^{2}$ is negligible compared to $\Phi_{2}(A_{j})$ as an approximation. From the proofs of Lemmas 18 and 24, we have

$$\mathrm{Var}[\hat{G}(A_{j})]\approx \Phi_{2}(A_{j})G(A_{j})-4\Phi_{2}(A_{j})G{\left(A_{j}\right)}^{2}$$

and

$$\mathrm{Cov}[{\hat{F}}_{3}(A_{j};B_{j},C_{j}),\hat{G}(A_{j})]=\Phi_{2}(A_{j})G(A_{j})[2G(A_{j})-2F_{3}(A_{j};B_{j},C_{j})-b_{j}(1-c_{j})-(1-b_{j})c_{j}].$$

Assuming that $\Phi_{2}{\left(A_{j}\right)}^{2}$ is negligible compared to $\Phi_{2}(A_{j})$, we have that

$$\Phi_{2}(A_{j})\mathrm{Var}[\hat{G}(A_{j})]\approx 0.$$

We therefore have that

$$\mathrm{Cov}[{\tilde{F}}_{3}(A_{j};B_{j},C_{j}),\tilde{G}(A_{j})]\approx \frac{1}{1-\Phi_{2}(A_{j})}Cov[{\hat{F}}_{3}(A_{j};B_{j},C_{j}),\hat{G}(A_{j})],$$

and thus by independence of loci we have

$$\begin{array}{l} Cov[{\tilde{F}}_{3}(A;B,C),\tilde{G}(A)]=\mathrm{Cov}[\frac{1}{J}\sum_{j=1}^{J}{\tilde{F}}_{3}(A_{j};B_{j},C_{j}),\frac{1}{J}\sum_{j=1}^{J}\tilde{G}(A_{j})]\\ =\frac{1}{J^{2}}\sum_{j=1}^{J}Cov[{\tilde{F}}_{3}(A_{j};B_{j},C_{j}),\tilde{G}(A_{j})]\\ \approx \frac{1}{J^{2}}\sum_{j=1}^{J}\frac{\Phi_{2}(A_{j})}{1-\Phi_{2}(A_{j})}G(A_{j})[2G(A_{j})-2F_{3}(A_{j};B_{j},C_{j})\\ -b_{j}(1-c_{j})-(1-b_{j})c_{j}].\;\;□ \end{array}$$

**Lemma 30.** Consider *J* independent polymorphic loci in populations *A*, *B*, and *C* with respective parametric reference allele frequencies $a_{j},b_{j},c_{j}\in (0,1)$, and suppose we take a random sample of $N(P_{j})$ individuals at locus *j* in population P∈{A,B,C}, some of which may be related or inbred, where individual $k\in \{1,2,\ldots ,N(P_{j})$} has ploidy *m_k_*. Moreover, assume that no individual is related to more than one other individual, which makes the terms $\Phi_{3}(P_{j}),\;\Phi_{4}(P_{j}),\;\Phi_{2,2}(P_{j}),\;\Phi_{2}{\left(P_{j}\right)}^{2}$, and any other terms of similar order negligible to $\Phi_{2}(P_{j})$. Based on this simplifying assumption, the estimators ${\overset{⌣}{F}}_{3}(A;B,C)$ and $\overset{⌣}{G}(A)$ have an approximate covariance

$$\mathrm{Cov}[{\overset{⌣}{F}}_{3}(A;B,C),\overset{⌣}{G}(A)]\approx \frac{1}{J^{2}}\sum_{j=1}^{J}\frac{\Phi_{2}(A_{j})\sum_{k=1}^{N(A_{j})}m_{k}}{\sum_{k=1}^{N(A_{j})}m_{k}-1}G(A_{j})[2G(A_{j})-2F_{3}(A_{j};B_{j},C_{j})-b_{j}(1-c_{j})-(1-b_{j})c_{j}].$$

*Proof*. Recall that

$${\overset{⌣}{F}}_{3}(A_{j};B_{j},C_{j})={\hat{F}}_{3}(A_{j};B_{j},C_{j})-\frac{1}{\sum_{k=1}^{N(A_{j})}m_{k}-1}\hat{G}(A_{j}),$$

where

$$\overset{⌣}{G}(A_{j})=\frac{\sum_{k=1}^{N(A_{j})}m_{k}}{\sum_{k=1}^{N(A_{j})}m_{k}-1}G(A_{j}).$$

It follows that

$$\begin{array}{l} Cov[{\overset{⌣}{F}}_{3}(A_{j};B_{j},C_{j}),\overset{⌣}{G}(A_{j})]=\mathrm{Cov}[{\hat{F}}_{3}(A_{j};B_{j},C_{j})-\frac{1}{\sum_{k=1}^{N(A_{j})}m_{k}-1}\hat{G}(A_{j}),\overset{⌣}{G}(A_{j})]\\ =\mathrm{Cov}[{\hat{F}}_{3}(A_{j};B_{j},C_{j}),\overset{⌣}{G}(A_{j})]-\frac{1}{\sum_{k=1}^{N(A_{j})}m_{k}-1}Cov[\hat{G}(A_{j}),\overset{⌣}{G}(A_{j})]\\ =\frac{\sum_{k=1}^{N(A_{j})}m_{k}}{\sum_{k=1}^{N(A_{j})}m_{k}-1}Cov[{\hat{F}}_{3}(A_{j};B_{j},C_{j}),\hat{G}(A_{j})]-\frac{\sum_{k=1}^{N(A_{j})}m_{k}}{{\left[\sum_{k=1}^{N(A_{j})}m_{k}-1\right]}^{2}}Var[\hat{G}(A_{j})]. \end{array}$$

From the proofs of Lemmas 18 and 24, we have

$$\mathrm{Var}[\hat{G}(A_{j})]\approx \Phi_{2}(A_{j})G(A_{j})-4\Phi_{2}(A_{j})G{\left(A_{j}\right)}^{2}$$

and

$$\mathrm{Cov}[{\hat{F}}_{3}(A_{j};B_{j},C_{j}),\hat{G}(A_{j})]=\Phi_{2}(A_{j})G(A_{j})[2G(A_{j})-2F_{3}(A_{j};B_{j},C_{j})-b_{j}(1-c_{j})-(1-b_{j})c_{j}].$$

By recalling the assumption that $\Phi_{2}{\left(A_{j}\right)}^{2}$ or any other term of similar magnitude (such as the $\Phi_{2}(A_{j})/[\sum_{k=1}^{N(A_{j})}m_{k}-1]$ term for $N(A_{j})$ large enough) is negligible compared to $\Phi_{2}(A_{j})$, we have that

$$\frac{1}{\sum_{k=1}^{N(A_{j})}m_{k}-1}Var[\hat{G}(A_{j})]\approx 0.$$

We therefore have that

$$\mathrm{Cov}[{\overset{⌣}{F}}_{3}(A_{j};B_{j},C_{j}),\overset{⌣}{G}(A_{j})]\approx \frac{\sum_{k=1}^{N(A_{j})}m_{k}}{\sum_{k=1}^{N(A_{j})}m_{k}-1}Cov[{\hat{F}}_{3}(A_{j};B_{j},C_{j}),\hat{G}(A_{j})],$$

and thus by independence of loci we have

$$\begin{array}{l} Cov[{\overset{⌣}{F}}_{3}(A;B,C),\overset{⌣}{G}(A)]=\mathrm{Cov}[\frac{1}{J}\sum_{j=1}^{J}{\overset{⌣}{F}}_{3}(A_{j};B_{j},C_{j}),\frac{1}{J}\sum_{j=1}^{J}\overset{⌣}{G}(A_{j})]\\ =\frac{1}{J^{2}}\sum_{j=1}^{J}Cov[{\overset{⌣}{F}}_{3}(A_{j};B_{j},C_{j}),\overset{⌣}{G}(A_{j})]\\ \approx \frac{1}{J^{2}}\sum_{j=1}^{J}\frac{\Phi_{2}(A_{j})\sum_{k=1}^{N(A_{j})}m_{k}}{\sum_{k=1}^{N(A_{j})}m_{k}-1}G(A_{j})[2G(A_{j})-2F_{3}(A_{j};B_{j},C_{j})\\ -b_{j}(1-c_{j})-(1-b_{j})c_{j}].\;\;□ \end{array}$$

**Proposition 31.** Consider *J* polymorphic loci in populations *A*, *B*, and *C* with respective parametric reference allele frequencies $a_{j},b_{j},c_{j}\in (0,1)$, and suppose we take a random sample of $N(P_{j})$ individuals at locus *j* in population P∈{A,B,C}, some of which may be related or inbred. Moreover, assume that no individual is related to more than one other individual, which makes the terms $\Phi_{3}(P_{j}),\;\Phi_{4}(P_{j}),\;\Phi_{2,2}(P_{j}),\;\Phi_{2}{\left(P_{j}\right)}^{2},\;\Phi_{2}(A_{j})\Phi_{2}(B_{j}),\;\Phi_{2}(A_{j})\Phi_{2}(C_{j})$, and $\Phi_{2}(B_{j})\Phi_{2}(C_{j})$ negligible to $\Phi_{2}(P_{j})$. Based on this simplifying assumption, the approximately unbiased ratio estimator ${\tilde{F}}_{3}(A;B,C|A)$ has approximate variance

$$\mathrm{Var}[{\tilde{F}}_{3}(A;B,C|A)]\approx \frac{F_{3}{\left(A;B,C\right)}^{2}}{4G{\left(A\right)}^{2}}[\frac{\mathrm{Var}[{\tilde{F}}_{3}(A;B,C)]}{F_{3}{\left(A;B,C\right)}^{2}}+\frac{\mathrm{Var}[\tilde{G}(A)]}{G{\left(A\right)}^{2}}-2\frac{\mathrm{Cov}[{\tilde{F}}_{3}(A;B,C),\tilde{G}(A)]}{F_{3}(A;B,C)G(A)}],$$

where the variances are

$$\begin{array}{l} Var[{\tilde{F}}_{3}(A;B,C)]\approx \frac{4}{J^{2}}\sum_{j=1}^{J}\Phi_{2}(A_{j})F_{3}(A_{j};B_{j},C_{j})G(A_{j})+\frac{1}{J^{2}}\sum_{j=1}^{J}\Phi_{2}(A_{j})F_{2}(B_{j},C_{j})G(A_{j})\\ +\frac{1}{J^{2}}\sum_{j=1}^{J}\Phi_{2}(B_{j})F_{2}(A_{j},C_{j})G(B_{j})+\frac{1}{J^{2}}\sum_{j=1}^{J}\Phi_{2}(C_{j})F_{2}(A_{j},B_{j})G(C_{j})\\ Var[\tilde{G}(A)]\approx \frac{1}{J^{2}}\sum_{j=1}^{J}\frac{\Phi_{2}(A_{j})}{1-2\Phi_{2}(A_{j})}G(A_{j})-\frac{4}{J^{2}}\sum_{j=1}^{J}\frac{\Phi_{2}(A_{j})}{1-2\Phi_{2}(A_{j})}G{\left(A_{j}\right)}^{2} \end{array}$$

and the covariance is

$$\mathrm{Cov}[{\tilde{F}}_{3}(A;B,C),\tilde{G}(A)]\approx \frac{1}{J^{2}}\sum_{j=1}^{J}\frac{\Phi_{2}(A_{j})}{1-\Phi_{2}(A_{j})}G(A_{j})[2G(A_{j})-2F_{3}(A_{j};B_{j},C_{j})-b_{j}(1-c_{j})-(1-b_{j})c_{j}].$$

*Proof*. Recall that

$${\tilde{F}}_{3}(A;B,C,|A)=\frac{{\tilde{F}}_{3}(A;B,C)}{2\tilde{G}(A)},$$

where ${\tilde{F}}_{3}(A;B,C)$ is an unbiased estimator for $F_{3}(A;B,C)$ and $\tilde{G}(A)$ is an unbiased estimator of *G*(*A*). Assuming that $X={\tilde{F}}_{3}(A;B,C)$ and $Y=2\tilde{G}(A)$, following the approximation in Wolter (2007) we have

$$\mathrm{Var}[{\tilde{F}}_{3}(A;B,C|A)]\approx \frac{F_{3}{\left(A;B,C\right)}^{2}}{4G{\left(A\right)}^{2}}[\frac{\mathrm{Var}[{\tilde{F}}_{3}(A;B,C)]}{F_{3}{\left(A;B,C\right)}^{2}}+\frac{\mathrm{Var}[\tilde{G}(A)]}{G{\left(A\right)}^{2}}-2\frac{\mathrm{Cov}[{\tilde{F}}_{3}(A;B,C),\tilde{G}(A)]}{F_{3}(A;B,C)G(A)}],$$

where $\mathrm{Var}[{\tilde{F}}_{3}(A;B,C)]$ is given in Proposition 25, $\mathrm{Var}[\tilde{G}(A)]$ in Lemma 18, and $\mathrm{Cov}[{\tilde{F}}_{3}(A;B,C),\tilde{G}(A)]$ in Lemma 29. □

**Proposition 32**. Consider *J* polymorphic loci in populations *A*, *B*, and *C* with respective parametric reference allele frequencies $a_{j},b_{j},c_{j}\in (0,1)$, and suppose we take a random sample of $N(P_{j})$ individuals at locus *j* in population P∈{A,B,C}, some of which may be related or inbred, where individual $k\in \{1,2,\ldots ,N(P_{j})$} has ploidy *m_k_*. Moreover, assume that no individual is related to more than one other individual, which makes the terms $\Phi_{3}(P_{j}),\;\Phi_{4}(P_{j}),\;\Phi_{2,2}(P_{j}),\;\Phi_{2}{\left(P_{j}\right)}^{2},\;\Phi_{2}(A_{j})\Phi_{2}(B_{j}),\;\Phi_{2}(A_{j})\Phi_{2}(C_{j}),\;\Phi_{2}(B_{j})\Phi_{2}(C_{j})$, and any other terms of similar order negligible to $\Phi_{2}(P_{j})$. Based on this simplifying assumption, the ratio estimator ${\overset{⌣}{F}}_{3}(A;B,C|A)$ has approximate variance

$$\mathrm{Var}[{\overset{⌣}{F}}_{3}(A;B,C|A)]\approx \frac{X{\left(A;B,C\right)}^{2}}{4Y{\left(A\right)}^{2}}[\frac{\mathrm{Var}[{\overset{⌣}{F}}_{3}(A;B,C)]}{X{\left(A;B,C\right)}^{2}}+\frac{\mathrm{Var}[\overset{⌣}{G}(A)]}{Y{\left(A\right)}^{2}}-2\frac{\mathrm{Cov}[{\overset{⌣}{F}}_{3}(A;B,C),\overset{⌣}{G}(A)]}{X(A;B,C)Y(A)}],$$

where the variances are

$$\begin{array}{l} Var[{\overset{⌣}{F}}_{3}(A;B,C)]\approx \frac{4}{J^{2}}\sum_{j=1}^{J}\Phi_{2}(A_{j})F_{3}(A_{j};B_{j},C_{j})G(A_{j})+\frac{1}{J^{2}}\sum_{j=1}^{J}\Phi_{2}(A_{j})F_{2}(B_{j},C_{j})G(A_{j})\\ +\frac{1}{J^{2}}\sum_{j=1}^{J}\Phi_{2}(B_{j})F_{2}(A_{j},C_{j})G(B_{j})+\frac{1}{J^{2}}\sum_{j=1}^{J}\Phi_{2}(C_{j})F_{2}(A_{j},B_{j})G(C_{j})\\ Var[\overset{⌣}{G}(A)]\approx \frac{1}{J^{2}}\sum_{j=1}^{J}\Phi_{2}(A_{j}){\left[\frac{\sum_{k=1}^{N(A_{j})}m_{k}}{\sum_{k=1}^{N(A_{j})}m_{k}-1}\right]}^{2}G(A_{j})-\frac{4}{J^{2}}\sum_{j=1}^{J}\Phi_{2}(A_{j}){\left[\frac{\sum_{k=1}^{N(A_{j})}m_{k}}{\sum_{k=1}^{N(A_{j})}m_{k}-1}\right]}^{2}G{\left(A_{j}\right)}^{2} \end{array}$$

the covariance is

$$\mathrm{Cov}[{\overset{⌣}{F}}_{3}(A;B,C),\overset{⌣}{G}(A)]\approx \frac{1}{J^{2}}\sum_{j=1}^{J}\frac{\Phi_{2}(A_{j})\sum_{k=1}^{N(A_{j})}m_{k}}{\sum_{k=1}^{N(A_{j})}m_{k}-1}G(A_{j})[2G(A_{j})-2F_{3}(A_{j};B_{j},C_{j})-b_{j}(1-c_{j})-(1-b_{j})c_{j}],$$

and where

$$\begin{array}{l} X(A;B,C)=F_{3}(A;B,C)+\frac{1}{J}\sum_{j=1}^{J}\frac{\Phi_{2}(A_{j})\sum_{k=1}^{N(A_{j})}m_{k}-1}{\sum_{k=1}^{N(A_{j})}m_{k}-1}G(A_{j})\\ Y(A)=\frac{1}{J}\sum_{j=1}^{J}\frac{[1-\Phi_{2}(A_{j})]\sum_{k=1}^{N(A_{j})}m_{k}}{\sum_{k=1}^{N(A_{j})}m_{k}-1}G(A_{j}). \end{array}$$

*Proof*. Recall that

$${\overset{⌣}{F}}_{3}(A;B,C,|A)=\frac{{\overset{⌣}{F}}_{3}(A;B,C)}{2\overset{⌣}{G}(A)},$$

where ${\overset{⌣}{F}}_{3}(A;B,C)$ is an estimator for $F_{3}(A;B,C)$ and $\overset{⌣}{G}(A)$ is an estimator of *G*(*A*). Assuming that $X={\overset{⌣}{F}}_{3}(A;B,C)$ and $Y=2\overset{⌣}{G}(A)$, following the approximation in Wolter (2007) we have

$$\mathrm{Var}[{\overset{⌣}{F}}_{3}(A;B,C|A)]\approx \frac{X{\left(A;B,C\right)}^{2}}{4Y{\left(A\right)}^{2}}[\frac{\mathrm{Var}[{\overset{⌣}{F}}_{3}(A;B,C)]}{X{\left(A;B,C\right)}^{2}}+\frac{\mathrm{Var}[\overset{⌣}{G}(A)]}{Y{\left(A\right)}^{2}}-2\frac{\mathrm{Cov}[{\overset{⌣}{F}}_{3}(A;B,C),\overset{⌣}{G}(A)]}{X(A;B,C)Y(A)}],$$

where $\mathrm{Var}[{\overset{⌣}{F}}_{3}(A;B,C)]$ is given in Proposition 26, $\mathrm{Var}[\overset{⌣}{G}(A)]$ in Lemma 18, $\mathrm{Cov}[{\overset{⌣}{F}}_{3}(A;B,C),\overset{⌣}{G}(A)]$ in Lemma 30, $X(A;B,C)=E[{\overset{⌣}{F}}_{3}(A;B,C)]$ in proof of Corollary 8, and $Y(A)=E[\overset{⌣}{G}(A)]$ in proof of Corollary 11. □

Lemma 33. Consider *J* independent polymorphic loci in populations *A*, *B*, *C*, and *D* with respective parametric reference allele frequencies $a_{j},b_{j},c_{j},d_{j}\in (0,1)$, and suppose we take a random sample of $N(P_{j})$ individuals at locus *j* in population P∈{A,B,C,D}, some of which may be related or inbred. Moreover, assume that no individual is related to more than one other individual, which makes the terms $\Phi_{3}(P_{j}),\;\Phi_{4}(P_{j}),\;\Phi_{2,2}(P_{j})$, and $\Phi_{2}{\left(P_{j}\right)}^{2}$ negligible to $\Phi_{2}(P_{j})$. Based on this simplifying assumption, the estimators ${\hat{F}}_{4}(A,B;C,D)$ and $\hat{G}(P),\;P\in \{A,B,C,D\},$ have approximate covariances

$$\begin{array}{l} Cov[{\hat{F}}_{4}(A,B;C,D),\hat{G}(A)]\approx \frac{1}{J^{2}}\sum_{j=1}^{J}\Phi_{2}(A_{j})G(A_{j})(1-2a_{j})(c_{j}-d_{j})\\ Cov[{\hat{F}}_{4}(A,B;C,D),\hat{G}(B)]\approx -\frac{1}{J^{2}}\sum_{j=1}^{J}\Phi_{2}(B_{j})G(B_{j})(1-2b_{j})(c_{j}-d_{j})\\ Cov[{\hat{F}}_{4}(A,B;C,D),\hat{G}(C)]\approx \frac{1}{J^{2}}\sum_{j=1}^{J}\Phi_{2}(C_{j})G(C_{j})(1-2c_{j})(a_{j}-b_{j})\\ Cov[{\hat{F}}_{4}(A,B;C,D),\hat{G}(D)]\approx -\frac{1}{J^{2}}\sum_{j=1}^{J}\Phi_{2}(D_{j})G(D_{j})(1-2d_{j})(a_{j}-b_{j}). \end{array}$$

*Proof*. From the proofs of Lemma 1 and Proposition 12, we that have

$$E[\hat{G}(P_{j})]=[1-\Phi_{2}(P_{j})]G(P_{j})$$

and

$$E[{\hat{F}}_{4}(A_{j},B_{j};C_{j},D_{j})]=F_{4}(A_{j},B_{j};C_{j},D_{j}),$$

yielding

$$E[{\hat{F}}_{4}(A_{j},B_{j};C_{j},D_{j})]E[\hat{G}(P_{j})]=[1-\Phi_{2}(P_{j})]F_{4}(A_{j},B_{j};C_{j},D_{j})G(P_{j}).$$

We first calculate

$$\begin{array}{l} E[{\hat{F}}_{4}(A_{j},B_{j};C_{j},D_{j})\hat{G}(A_{j})]=E[({\hat{a}}_{j}-{\hat{b}}_{j})({\hat{c}}_{j}-{\hat{d}}_{j}){\hat{a}}_{j}(1-{\hat{a}}_{j})]\\ =E[({\hat{a}}_{j}-{\hat{b}}_{j})({\hat{a}}_{j}-{\hat{a}}_{j}^{2})]E[{\hat{c}}_{j}-{\hat{d}}_{j}]\\ =[E[{\hat{a}}_{j}^{2}]-E[{\hat{a}}_{j}^{3}]-E[{\hat{a}}_{j}]E[{\hat{b}}_{j}]+E[{\hat{a}}_{j}^{2}]E[{\hat{b}}_{j}]][E[{\hat{c}}_{j}]-E[{\hat{d}}_{j}]]\\ \approx [a_{j}^{2}+\Phi_{2}(A_{j})a_{j}(1-a_{j})-[a_{j}^{3}+3\Phi_{2}(A_{j})a_{j}^{3}(1-a_{j})-a_{j}b_{j}\\ +[a_{j}^{2}+\Phi_{2}(A_{j})a_{j}(1-a_{j})]b_{j}](c_{j}-d_{j})\\ =(a_{j}^{2}-a_{j}^{3}-a_{j}b_{j}+a_{j}^{2}b_{j})(c_{j}-d_{j})+\Phi_{2}(A_{j})a_{j}(1-a_{j})(1-3a_{j}+b_{j})(c_{j}-d_{j})\\ =(a_{j}-b_{j})(c_{j}-d_{j})a_{j}(1-a_{j})+\Phi_{2}(A_{j})a_{j}(1-a_{j})[1-2a_{j}-(a_{j}-b_{j})](c_{j}-d_{j})\\ =F_{4}(A_{j},B_{j};C_{j},D_{j})G(A_{j})-\Phi_{2}(A_{j})F_{4}(A_{j},B_{j};C_{j},D_{j})G(A_{j})\\ +\Phi_{2}(A_{j})G(A_{j})(1-2a_{j})(c_{j}-d_{j})\\ =[1-\Phi_{2}(A_{j})]F_{4}(A_{j},B_{j};C_{j},D_{j})G(A_{j})+\Phi_{2}(A_{j})G(A_{j})(1-2a_{j})(c_{j}-d_{j}). \end{array}$$

Hence, we have that

$$\begin{array}{l} Cov[{\hat{F}}_{4}(A_{j},B_{j};C_{j},D_{j}),\hat{G}(A_{j})]=E[{\hat{F}}_{4}(A_{j},B_{j};C_{j},D_{j})\hat{G}(A_{j})]-E[{\hat{F}}_{4}(A_{j},B_{j};C_{j},D_{j})]E[\hat{G}(A_{j})]\\ \approx [1-\Phi_{2}(A_{j})]F_{4}(A_{j},B_{j};C_{j},D_{j})G(A_{j})+\Phi_{2}(A_{j})G(A_{j})(1-2a_{j})(c_{j}-d_{j})\\ -[1-\Phi_{2}(A_{j})]F_{4}(A_{j},B_{j};C_{j},D_{j})G(A_{j})\\ =\Phi_{2}(A_{j})G(A_{j})(1-2a_{j})(c_{j}-d_{j}). \end{array}$$

Similarly, we have that

$$\begin{array}{l} E[{\hat{F}}_{4}(A_{j},B_{j};C_{j},D_{j})\hat{G}(B_{j})]=E[({\hat{a}}_{j}-{\hat{b}}_{j})({\hat{c}}_{j}-{\hat{d}}_{j}){\hat{b}}_{j}(1-{\hat{b}}_{j})]\\ =E[({\hat{a}}_{j}-{\hat{b}}_{j})({\hat{b}}_{j}-{\hat{b}}_{j}^{2})]E[{\hat{c}}_{j}-{\hat{d}}_{j}]\\ =-E[({\hat{b}}_{j}-{\hat{a}}_{j})({\hat{b}}_{j}-{\hat{b}}_{j}^{2})]E[{\hat{c}}_{j}-{\hat{d}}_{j}]\\ =-[E[{\hat{b}}_{j}^{2}]-E[{\hat{b}}_{j}^{3}]-E[{\hat{a}}_{j}]E[{\hat{b}}_{j}]+E[{\hat{a}}_{j}]E[{\hat{b}}_{j}^{2}]][E[{\hat{c}}_{j}]-E[{\hat{d}}_{j}]]\\ \approx -[b_{j}^{2}+\Phi_{2}(B_{j})b_{j}(1-b_{j})-[b_{j}^{3}+3\Phi_{2}(B_{j})b_{j}^{3}(1-b_{j})-a_{j}b_{j}\\ +a_{j}[b_{j}^{2}+\Phi_{2}(B_{j})b_{j}(1-b_{j})]](c_{j}-d_{j})\\ =-(b_{j}^{2}-b_{j}^{3}-a_{j}b_{j}+a_{j}b_{j}^{2})(c_{j}-d_{j})-\Phi_{2}(B_{j})b_{j}(1-b_{j})(1-3b_{j}+a_{j})(c_{j}-d_{j})\\ =(a_{j}-b_{j})(c_{j}-d_{j})b_{j}(1-b_{j})-\Phi_{2}(B_{j})b_{j}(1-b_{j})[1-2b_{j}+(a_{j}-b_{j})](c_{j}-d_{j})\\ =F_{4}(A_{j},B_{j};C_{j},D_{j})G(A_{j})-\Phi_{2}(B_{j})F_{4}(A_{j},B_{j};C_{j},D_{j})G(B_{j})\\ -\Phi_{2}(B_{j})G(B_{j})(1-2b_{j})(c_{j}-d_{j})\\ =[1-\Phi_{2}(B_{j})]F_{4}(A_{j},B_{j};C_{j},D_{j})G(B_{j})-\Phi_{2}(B_{j})G(B_{j})(1-2b_{j})(c_{j}-d_{j}). \end{array}$$

Hence, we have that

$$\begin{array}{l} Cov[{\hat{F}}_{4}(A_{j},B_{j};C_{j},D_{j}),\hat{G}(B_{j})]=E[{\hat{F}}_{4}(A_{j},B_{j};C_{j},D_{j})\hat{G}(B_{j})]-E[{\hat{F}}_{4}(A_{j},B_{j};C_{j},D_{j})]E[\hat{G}(B_{j})]\\ \approx [1-\Phi_{2}(B_{j})]F_{4}(A_{j},B_{j};C_{j},D_{j})G(B_{j})-\Phi_{2}(B_{j})G(B_{j})(1-2b_{j})(c_{j}-d_{j})\\ -[1-\Phi_{2}(B_{j})]F_{4}(A_{j},B_{j};C_{j},D_{j})G(B_{j})\\ =-\Phi_{2}(B_{j})G(B_{j})(1-2b_{j})(c_{j}-d_{j}). \end{array}$$

Parallel to the derivation for *P *=* A*, we have

$$\mathrm{Cov}[{\hat{F}}_{4}(A_{j},B_{j};C_{j},D_{j}),\hat{G}(C_{j})]=\Phi_{2}(C_{j})G(C_{j})(1-2c_{j})(a_{j}-b_{j})$$

and parallel to the derivation for *P *=* B*, we have

$$\mathrm{Cov}[{\hat{F}}_{4}(A_{j},B_{j};C_{j},D_{j}),\hat{G}(D_{j})]=-\Phi_{2}(D_{j})G(D_{j})(1-2d_{j})(a_{j}-b_{j}).$$

We know that by independence of loci we have

$$\begin{array}{l} Cov[{\hat{F}}_{4}(A,B;C,D),\hat{G}(P)]=\mathrm{Cov}[\frac{1}{J}\sum_{j=1}^{J}{\hat{F}}_{4}(A_{j},B_{j};C_{j},D_{j}),\frac{1}{J}\sum_{j=1}^{J}\hat{G}(P_{j})]\\ =\frac{1}{J^{2}}\sum_{j=1}^{J}Cov[{\hat{F}}_{4}(A_{j},B_{j};C_{j},D_{j}),\hat{G}(P_{j})] \end{array}$$

which gives

$$\begin{array}{l} Cov[{\hat{F}}_{4}(A,B;C,D),\hat{G}(A)]\approx \frac{1}{J^{2}}\sum_{j=1}^{J}\Phi_{2}(A_{j})G(A_{j})(1-2a_{j})(c_{j}-d_{j})\\ Cov[{\hat{F}}_{4}(A,B;C,D),\hat{G}(B)]\approx -\frac{1}{J^{2}}\sum_{j=1}^{J}\Phi_{2}(B_{j})G(B_{j})(1-2b_{j})(c_{j}-d_{j})\\ Cov[{\hat{F}}_{4}(A,B;C,D),\hat{G}(C)]\approx \frac{1}{J^{2}}\sum_{j=1}^{J}\Phi_{2}(C_{j})G(C_{j})(1-2c_{j})(a_{j}-b_{j})\\ Cov[{\hat{F}}_{4}(A,B;C,D),\hat{G}(D)]\approx -\frac{1}{J^{2}}\sum_{j=1}^{J}\Phi_{2}(D_{j})G(D_{j})(1-2d_{j})(a_{j}-b_{j}).\;□ \end{array}$$

**Proposition 34**. Consider *J* polymorphic loci in populations *A*, *B*, *C*, and *D* with respective parametric reference allele frequencies $a_{j},b_{j},c_{j},d_{j}\in (0,1)$, and suppose we take a random sample of $N(P_{j})$ individuals at locus *j* in population P∈{A,B,C,D}, some of which may be related or inbred. Moreover, assume that no individual is related to more than one other individual, which makes the terms $\Phi_{3}(P_{j}),\;\Phi_{4}(P_{j}),\;\Phi_{2,2}(P_{j})$, and $\Phi_{2}{\left(P_{j}\right)}^{2}$ negligible to $\Phi_{2}(P_{j})$. Based on this simplifying assumption, the ratio estimator ${\hat{F}}_{4}(A,B;C,D|P)$ has approximate variance

$$\begin{array}{l} Var[{\hat{F}}_{4}(A,B;C,D|P)]\approx \frac{F_{4}{\left(A,B;C,D\right)}^{2}}{E{\left[\hat{G}(P)\right]}^{2}}[\frac{\mathrm{Var}[{\hat{F}}_{4}(A,B;C,D)]}{F_{4}{\left(A,B;C,D\right)}^{2}}+\frac{\mathrm{Var}[\hat{G}(A)]}{E{\left[\hat{G}(P)\right]}^{2}}\\ -2\frac{\mathrm{Cov}[{\hat{F}}_{4}(A,B;C,D),\hat{G}(P)]}{F_{4}(A,B;C,D)E[\hat{G}(P)]}], \end{array}$$

where the expectation is

$$E[\hat{G}(P)]=G(P)-\frac{1}{J}\sum_{j=1}^{J}\Phi_{2}(P_{j})G(P_{j})$$

the variances are

$$\begin{array}{l} Var[{\hat{F}}_{4}(A,B;C,D)]\approx \frac{1}{J^{2}}\sum_{j=1}^{J}[\Phi_{2}(C_{j})g(C_{j})+\Phi_{2}(D_{j})G(D_{j})]F_{2}(A_{j},B_{j})\\ +\frac{1}{J^{2}}\sum_{j=1}^{J}[\Phi_{2}(A_{j})G(A_{j})+\Phi_{2}(B_{j})G(B_{j})]F_{2}(C_{j},D_{j})\\ Var[\hat{G}(P)]\approx \frac{1}{J^{2}}\sum_{j=1}^{J}\Phi_{2}(P_{j})G(P_{j})-\frac{4}{J^{2}}\sum_{j=1}^{J}\Phi_{2}(P_{j})G{\left(P_{j}\right)}^{2} \end{array}$$

and the covariances are

$$\begin{array}{l} Cov[{\hat{F}}_{4}(A,B;C,D),\hat{G}(A)]\approx \frac{1}{J^{2}}\sum_{j=1}^{J}\Phi_{2}(A_{j})G(A_{j})(1-2a_{j})(c_{j}-d_{j})\\ Cov[{\hat{F}}_{4}(A,B;C,D),\hat{G}(B)]\approx -\frac{1}{J^{2}}\sum_{j=1}^{J}\Phi_{2}(B_{j})G(B_{j})(1-2b_{j})(c_{j}-d_{j})\\ Cov[{\hat{F}}_{4}(A,B;C,D),\hat{G}(C)]\approx \frac{1}{J^{2}}\sum_{j=1}^{J}\Phi_{2}(C_{j})G(C_{j})(1-2c_{j})(a_{j}-b_{j})\\ Cov[{\hat{F}}_{4}(A,B;C,D),\hat{G}(D)]\approx -\frac{1}{J^{2}}\sum_{j=1}^{J}\Phi_{2}(D_{j})G(D_{j})(1-2d_{j})(a_{j}-b_{j}). \end{array}$$

*Proof*. Recall that

$${\hat{F}}_{4}(A,B;C,D|P)=\frac{{\hat{F}}_{4}(A,B;C,D)}{\hat{G}(P)},$$

where ${\hat{F}}_{4}(A,B;C,D)$ is an unbiased estimator for $F_{4}(A,B;C,D)$. Assuming that $X={\hat{F}}_{4}(A,B;C,D)$ and $Y=\hat{G}(P)$, following the approximation in Wolter (2007) we have

$$\begin{array}{l} Var[{\hat{F}}_{4}(A,B;C,D|P)]\approx \frac{F_{4}{\left(A,B;C,D\right)}^{2}}{E{\left[\hat{G}(P)\right]}^{2}}[\frac{\mathrm{Var}[{\hat{F}}_{4}(A,B;C,D)]}{F_{4}{\left(A,B;C,D\right)}^{2}}+\frac{\mathrm{Var}[\hat{G}(A)]}{E{\left[\hat{G}(P)\right]}^{2}}\\ -2\frac{\mathrm{Cov}[{\hat{F}}_{4}(A,B;C,D),\hat{G}(P)]}{F_{4}(A,B;C,D)E[\hat{G}(P)]}], \end{array}$$

where $E[\hat{G}(P)]$ is given in Lemma 1, $\mathrm{Var}[{\hat{F}}_{4}(A,B;C,D)]$ in Proposition 27, $\mathrm{Var}[\hat{G}(P)]$ in Lemma 18, and $\mathrm{Cov}[{\hat{F}}_{4}(A,B;C,D),\hat{G}(P)]$ in Lemma 33 for each population P∈{A,B,C,D}. □

Lemma 35. Consider *J* independent polymorphic loci in populations *A*, *B*, *C*, and *D* with respective parametric reference allele frequencies $a_{j},b_{j},c_{j},d_{j}\in (0,1)$, and suppose we take a random sample of $N(P_{j})$ individuals at locus *j* in population P∈{A,B,C,D}, some of which may be related or inbred. Moreover, assume that no individual is related to more than one other individual, which makes the terms $\Phi_{3}(P_{j}),\;\Phi_{4}(P_{j}),\;\Phi_{2,2}(P_{j})$, and $\Phi_{2}{\left(P_{j}\right)}^{2}$ negligible to $\Phi_{2}(P_{j})$. Based on this simplifying assumption, the unbiased estimators ${\hat{F}}_{4}(A,B;C,D)$ and $\tilde{G}(P),\;P\in \{A,B,C,D\},$ have approximate covariances

$$\begin{array}{l} Cov[{\hat{F}}_{4}(A,B;C,D),\tilde{G}(A)]\approx \frac{1}{J^{2}}\sum_{j=1}^{J}\frac{\Phi_{2}(A_{j})}{1-\Phi_{2}(A_{j})}G(A_{j})(1-2a_{j})(c_{j}-d_{j})\\ Cov[{\hat{F}}_{4}(A,B;C,D),\tilde{G}(B)]\approx -\frac{1}{J^{2}}\sum_{j=1}^{J}\frac{\Phi_{2}(B_{j})}{1-\Phi_{2}(B_{j})}G(B_{j})(1-2b_{j})(c_{j}-d_{j})\\ Cov[{\hat{F}}_{4}(A,B;C,D),\tilde{G}(C)]\approx \frac{1}{J^{2}}\sum_{j=1}^{J}\frac{\Phi_{2}(C_{j})}{1-\Phi_{2}(C_{j})}G(C_{j})(1-2c_{j})(a_{j}-b_{j})\\ Cov[{\hat{F}}_{4}(A,B;C,D),\tilde{G}(D)]\approx -\frac{1}{J^{2}}\sum_{j=1}^{J}\frac{\Phi_{2}(D_{j})}{1-\Phi_{2}(D_{j})}G(D_{j})(1-2d_{j})(a_{j}-b_{j}). \end{array}$$

*Proof*. Recall that $\tilde{G}(P_{j})=\hat{G}(P_{j})/[1-\Phi_{2}(P_{j})]$. It follows that

$$\mathrm{Cov}[{\hat{F}}_{4}(A_{j},B_{j};C_{j},D_{j}),\tilde{G}(P_{j})]=\frac{1}{1-\Phi_{2}(P_{j})}Cov[{\hat{F}}_{4}(A_{j},B_{j};C_{j},D_{j}),\hat{G}(P_{j})].$$

From the proof of Lemma 33, we have

$$\begin{array}{l} Cov[{\hat{F}}_{4}(A_{j},B_{j};C_{j},D_{j}),\tilde{G}(A_{j})]\approx \frac{\Phi_{2}(A_{j})}{1-\Phi_{2}(A_{j})}G(A_{j})(1-2a_{j})(c_{j}-d_{j})\\ Cov[{\hat{F}}_{4}(A_{j},B_{j};C_{j},D_{j}),\tilde{G}(B_{j})]\approx -\frac{\Phi_{2}(B_{j})}{1-\Phi_{2}(B_{j})}G(B_{j})(1-2b_{j})(c_{j}-d_{j})\\ Cov[{\hat{F}}_{4}(A_{j},B_{j};C_{j},D_{j}),\tilde{G}(C_{j})]\approx \frac{\Phi_{2}(C_{j})}{1-\Phi_{2}(C_{j})}G(C_{j})(1-2c_{j})(a_{j}-b_{j})\\ Cov[{\hat{F}}_{4}(A_{j},B_{j};C_{j},D_{j}),\tilde{G}(D_{j})]\approx -\frac{\Phi_{2}(D_{j})}{1-\Phi_{2}(D_{j})}G(D_{j})(1-2d_{j})(a_{j}-b_{j}), \end{array}$$

yielding

$$\begin{array}{l} Cov[{\hat{F}}_{4}(A,B;C,D),\tilde{G}(A)]\approx \frac{1}{J^{2}}\sum_{j=1}^{J}\frac{\Phi_{2}(A_{j})}{1-\Phi_{2}(A_{j})}G(A_{j})(1-2a_{j})(c_{j}-d_{j})\\ Cov[{\hat{F}}_{4}(A,B;C,D),\tilde{G}(B)]\approx -\frac{1}{J^{2}}\sum_{j=1}^{J}\frac{\Phi_{2}(B_{j})}{1-\Phi_{2}(B_{j})}G(B_{j})(1-2b_{j})(c_{j}-d_{j})\\ Cov[{\hat{F}}_{4}(A,B;C,D),\tilde{G}(C)]\approx \frac{1}{J^{2}}\sum_{j=1}^{J}\frac{\Phi_{2}(C_{j})}{1-\Phi_{2}(C_{j})}G(C_{j})(1-2c_{j})(a_{j}-b_{j})\\ Cov[{\hat{F}}_{4}(A,B;C,D),\tilde{G}(D)]\approx -\frac{1}{J^{2}}\sum_{j=1}^{J}\frac{\Phi_{2}(D_{j})}{1-\Phi_{2}(D_{j})}G(D_{j})(1-2d_{j})(a_{j}-b_{j}).\;□ \end{array}$$

Lemma 36. Consider *J* independent polymorphic loci in populations *A*, *B*, *C*, and *D* with respective parametric reference allele frequencies $a_{j},b_{j},c_{j},d_{j}\in (0,1)$, and suppose we take a random sample of $N(P_{j})$ individuals at locus *j* in population P∈{A,B,C,D}, some of which may be related or inbred, where individual $k\in \{1,2,\ldots ,N(P_{j})$} has ploidy *m_k_*. Moreover, assume that no individual is related to more than one other individual, which makes the terms $\Phi_{3}(P_{j}),\;\Phi_{4}(P_{j}),\;\Phi_{2,2}(P_{j})$, and $\Phi_{2}{\left(P_{j}\right)}^{2}$ negligible to $\Phi_{2}(P_{j})$. Based on this simplifying assumption, the estimators ${\hat{F}}_{4}(A,B;C,D)$ and $\overset{⌣}{G}(P),\;P\in \{A,B,C,D\},$ have approximate covariances

$$\begin{array}{l} Cov[{\hat{F}}_{4}(A,B;C,D),\overset{⌣}{G}(A)]\approx \frac{1}{J^{2}}\sum_{j=1}^{J}\frac{\Phi_{2}(A_{j})\sum_{k=1}^{N(A_{j})}m_{k}}{\sum_{k=1}^{N(A_{j})}m_{k}-1}G(A_{j})(1-2a_{j})(c_{j}-d_{j})\\ Cov[{\hat{F}}_{4}(A,B;C,D),\overset{⌣}{G}(B)]\approx -\frac{1}{J^{2}}\sum_{j=1}^{J}\frac{\Phi_{2}(B_{j})\sum_{k=1}^{N(B_{j})}m_{k}}{\sum_{k=1}^{N(B_{j})}m_{k}-1}G(B_{j})(1-2b_{j})(c_{j}-d_{j})\\ Cov[{\hat{F}}_{4}(A,B;C,D),\overset{⌣}{G}(C)]\approx \frac{1}{J^{2}}\sum_{j=1}^{J}\frac{\Phi_{2}(C_{j})\sum_{k=1}^{N(C_{j})}m_{k}}{\sum_{k=1}^{N(C_{j})}m_{k}-1}G(C_{j})(1-2c_{j})(a_{j}-b_{j})\\ Cov[{\hat{F}}_{4}(A,B;C,D),\overset{⌣}{G}(D)]\approx -\frac{1}{J^{2}}\sum_{j=1}^{J}\frac{\Phi_{2}(D_{j})\sum_{k=1}^{N(D_{j})}m_{k}}{\sum_{k=1}^{N(D_{j})}m_{k}-1}G(D_{j})(1-2d_{j})(a_{j}-b_{j}). \end{array}$$

*Proof*. Recall that

$$\overset{⌣}{G}(P_{j})=\frac{\sum_{k=1}^{N(P_{j})}m_{k}}{\sum_{k=1}^{N(P_{j})}m_{k}-1}\hat{G}(P_{j}).$$

It follows that

$$\mathrm{Cov}[{\hat{F}}_{4}(A_{j},B_{j};C_{j},D_{j}),\overset{⌣}{G}(P_{j})]=\frac{\sum_{k=1}^{N(P_{j})}m_{k}}{\sum_{k=1}^{N(P_{j})}m_{k}-1}Cov[{\hat{F}}_{4}(A_{j},B_{j};C_{j},D_{j}),\hat{G}(P_{j})].$$

From the proof of Lemma 33, we have

$$\begin{array}{l} Cov[{\hat{F}}_{4}(A_{j},B_{j};C_{j},D_{j}),\overset{⌣}{G}(A_{j})]\approx \frac{\Phi_{2}(A_{j})\sum_{k=1}^{N(A_{j})}m_{k}}{\sum_{k=1}^{N(A_{j})}m_{k}-1}G(A_{j})(1-2a_{j})(c_{j}-d_{j})\\ Cov[{\hat{F}}_{4}(A_{j},B_{j};C_{j},D_{j}),\overset{⌣}{G}(B_{j})]\approx -\frac{\Phi_{2}(G_{j})\sum_{k=1}^{N(G_{j})}m_{k}}{\sum_{k=1}^{N(G_{j})}m_{k}-1}G(B_{j})(1-2b_{j})(c_{j}-d_{j})\\ Cov[{\hat{F}}_{4}(A_{j},B_{j};C_{j},D_{j}),\overset{⌣}{G}(C_{j})]\approx \frac{\Phi_{2}(C_{j})\sum_{k=1}^{N(C_{j})}m_{k}}{\sum_{k=1}^{N(C_{j})}m_{k}-1}G(C_{j})(1-2c_{j})(a_{j}-b_{j})\\ Cov[{\hat{F}}_{4}(A_{j},B_{j};C_{j},D_{j}),\overset{⌣}{G}(D_{j})]\approx -\frac{\Phi_{2}(D_{j})\sum_{k=1}^{N(D_{j})}m_{k}}{\sum_{k=1}^{N(D_{j})}m_{k}-1}G(D_{j})(1-2d_{j})(a_{j}-b_{j}), \end{array}$$

yielding

$$\begin{array}{l} Cov[{\hat{F}}_{4}(A,B;C,D),\overset{⌣}{G}(A)]\approx \frac{1}{J^{2}}\sum_{j=1}^{J}\frac{\Phi_{2}(A_{j})\sum_{k=1}^{N(A_{j})}m_{k}}{\sum_{k=1}^{N(A_{j})}m_{k}-1}G(A_{j})(1-2a_{j})(c_{j}-d_{j})\\ Cov[{\hat{F}}_{4}(A,B;C,D),\overset{⌣}{G}(B)]\approx -\frac{1}{J^{2}}\sum_{j=1}^{J}\frac{\Phi_{2}(B_{j})\sum_{k=1}^{N(B_{j})}m_{k}}{\sum_{k=1}^{N(B_{j})}m_{k}-1}G(B_{j})(1-2b_{j})(c_{j}-d_{j})\\ Cov[{\hat{F}}_{4}(A,B;C,D),\overset{⌣}{G}(C)]\approx \frac{1}{J^{2}}\sum_{j=1}^{J}\frac{\Phi_{2}(C_{j})\sum_{k=1}^{N(C_{j})}m_{k}}{\sum_{k=1}^{N(C_{j})}m_{k}-1}G(C_{j})(1-2c_{j})(a_{j}-b_{j})\\ Cov[{\hat{F}}_{4}(A,B;C,D),\overset{⌣}{G}(D)]\approx -\frac{1}{J^{2}}\sum_{j=1}^{J}\frac{\Phi_{2}(D_{j})\sum_{k=1}^{N(D_{j})}m_{k}}{\sum_{k=1}^{N(D_{j})}m_{k}-1}G(D_{j})(1-2d_{j})(a_{j}-b_{j}).\;□ \end{array}$$

**Proposition 37.** Consider *J* polymorphic loci in populations *A*, *B*, *C*, and *D* with respective parametric reference allele frequencies $a_{j},b_{j},c_{j},d_{j}\in (0,1)$, and suppose we take a random sample of $N(P_{j})$ individuals at locus *j* in population P∈{A,B,C,D}, some of which may be related or inbred. Moreover, assume that no individual is related to more than one other individual, which makes the terms $\Phi_{3}(P_{j}),\;\Phi_{4}(P_{j}),\;\Phi_{2,2}(P_{j})$, and $\Phi_{2}{\left(P_{j}\right)}^{2}$ negligible to $\Phi_{2}(P_{j})$. Based on this simplifying assumption, the approximately unbiased ratio estimator ${\tilde{F}}_{4}(A,B;C,D|P)$ has approximate variance

$$\begin{array}{l} Var[{\tilde{F}}_{4}(A,B;C,D|P)]\approx \frac{F_{4}{\left(A,B;C,D\right)}^{2}}{G{\left(P\right)}^{2}}[\frac{\mathrm{Var}[{\hat{F}}_{4}(A,B;C,D)]}{F_{4}{\left(A,B;C,D\right)}^{2}}+\frac{\mathrm{Var}[\tilde{G}(A)]}{G{\left(P\right)}^{2}}\\ -2\frac{\mathrm{Cov}[{\hat{F}}_{4}(A,B;C,D),\tilde{G}(P)]}{F_{4}(A,B;C,D)G(P)}], \end{array}$$

where the variances are

$$\begin{array}{l} Var[{\hat{F}}_{4}(A,B;C,D)]\approx \frac{1}{J^{2}}\sum_{j=1}^{J}[\Phi_{2}(C_{j})G(C_{j})+\Phi_{2}(D_{j})G(D_{j})]F_{2}(A_{j},B_{j})\\ +\frac{1}{J^{2}}\sum_{j=1}^{J}[\Phi_{2}(A_{j})G(A_{j})+\Phi_{2}(B_{j})G(B_{j})]F_{2}(C_{j},D_{j})\\ Var[\tilde{G}(P)]\approx \frac{1}{J^{2}}\sum_{j=1}^{J}\frac{\Phi_{2}(P_{j})}{1-2\Phi_{2}(P_{j})}G(P_{j})-\frac{4}{J^{2}}\sum_{j=1}^{J}\frac{\Phi_{2}(P_{j})}{1-2\Phi_{2}(P_{j})}G{\left(P_{j}\right)}^{2} \end{array}$$

and the covariances are

$$\begin{array}{l} Cov[{\hat{F}}_{4}(A,B;C,D),\tilde{G}(A)]\approx \frac{1}{J^{2}}\sum_{j=1}^{J}\frac{\Phi_{2}(A_{j})}{1-\Phi_{2}(A_{j})}G(A_{j})(1-2a_{j})(c_{j}-d_{j})\\ Cov[{\hat{F}}_{4}(A,B;C,D),\tilde{G}(B)]\approx -\frac{1}{J^{2}}\sum_{j=1}^{J}\frac{\Phi_{2}(B_{j})}{1-\Phi_{2}(B_{j})}G(B_{j})(1-2b_{j})(c_{j}-d_{j})\\ Cov[{\hat{F}}_{4}(A,B;C,D),\tilde{G}(C)]\approx \frac{1}{J^{2}}\sum_{j=1}^{J}\frac{\Phi_{2}(C_{j})}{1-\Phi_{2}(C_{j})}G(C_{j})(1-2c_{j})(a_{j}-b_{j})\\ Cov[{\hat{F}}_{4}(A,B;C,D),\tilde{G}(D)]\approx -\frac{1}{J^{2}}\sum_{j=1}^{J}\frac{\Phi_{2}(D_{j})}{1-\Phi_{2}(D_{j})}G(D_{j})(1-2d_{j})(a_{j}-b_{j}). \end{array}$$

*Proof*. Recall that

$${\tilde{F}}_{4}(A,B;C,D|P)=\frac{{\hat{F}}_{4}(A,B;C,D)}{\tilde{G}(P)},$$

where ${\hat{F}}_{4}(A,B;C,D)$ is an unbiased estimator for $F_{4}(A,B;C,D)$ and $\tilde{G}(P)$ is an unbiased estimator of *G*(*P*). Assuming that $X={\hat{F}}_{4}(A,B;C,D)$ and $Y=\tilde{G}(P)$, following the approximation in Wolter (2007) we have

$$\begin{array}{l} Var[{\tilde{F}}_{4}(A,B;C,D|P)]\approx \frac{F_{4}{\left(A,B;C,D\right)}^{2}}{G{\left(P\right)}^{2}}[\frac{\mathrm{Var}[{\hat{F}}_{4}(A,B;C,D)]}{F_{4}{\left(A,B;C,D\right)}^{2}}+\frac{\mathrm{Var}[\tilde{G}(A)]}{G{\left(P\right)}^{2}}\\ -2\frac{\mathrm{Cov}[{\hat{F}}_{4}(A,B;C,D),\tilde{G}(P)]}{F_{4}(A,B;C,D)G(P)}], \end{array}$$

where $\mathrm{Var}[{\hat{F}}_{4}(A,B;C,D)]$ is given in Proposition 27, $\mathrm{Var}[\tilde{G}(P)]$ in Lemma 18, and $\mathrm{Cov}[{\hat{F}}_{4}(A,B;C,D),\tilde{G}(P)]$ in Lemma 35 for each population P∈{A,B,C,D}. □

**Proposition 38.** Consider *J* polymorphic loci in populations *A*, *B*, *C*, and *D* with respective parametric reference allele frequencies $a_{j},b_{j},c_{j},d_{j}\in (0,1)$, and suppose we take a random sample of $N(P_{j})$ individuals at locus *j* in population P∈{A,B,C,D}, some of which may be related or inbred, where individual $k\in \{1,2,\ldots ,N(P_{j})$} has ploidy *m_k_*. Moreover, assume that no individual is related to more than one other individual, which makes the terms $\Phi_{3}(P_{j}),\;\Phi_{4}(P_{j}),\;\Phi_{2,2}(P_{j})$, and $\Phi_{2}{\left(P_{j}\right)}^{2}$ negligible to $\Phi_{2}(P_{j})$. Based on this simplifying assumption, the ratio estimator ${\overset{⌣}{F}}_{4}(A,B;C,D|P)$ has approximate variance

$$\begin{array}{l} Var[{\overset{⌣}{F}}_{4}(A,B;C,D|P)]\approx \frac{F_{4}{\left(A,B;C,D\right)}^{2}}{Y{\left(P\right)}^{2}}[\frac{\mathrm{Var}[{\hat{F}}_{4}(A,B;C,D)]}{F_{4}{\left(A,B;C,D\right)}^{2}}+\frac{\mathrm{Var}[\overset{⌣}{G}(A)]}{Y{\left(P\right)}^{2}}\\ -2\frac{\mathrm{Cov}[{\hat{F}}_{4}(A,B;C,D),\overset{⌣}{G}(P)]}{F_{4}(A,B;C,D)Y(P)}], \end{array}$$

where the variances are

$$\begin{array}{l} Var[{\hat{F}}_{4}(A,B;C,D)]\approx \frac{1}{J^{2}}\sum_{j=1}^{J}[\Phi_{2}(C_{j})G(C_{j})+\Phi_{2}(D_{j})G(D_{j})]F_{2}(A_{j},B_{j})\\ +\frac{1}{J^{2}}\sum_{j=1}^{J}[\Phi_{2}(A_{j})G(A_{j})+\Phi_{2}(B_{j})G(B_{j})]F_{2}(C_{j},D_{j})\\ Var[\overset{⌣}{G}(P)]\approx \frac{1}{J^{2}}\sum_{j=1}^{J}\Phi_{2}(P_{j}){\left[\frac{\sum_{k=1}^{N(P_{j})}m_{k}}{\sum_{k=1}^{N(P_{j})}m_{k}-1}\right]}^{2}G(P_{j})-\frac{4}{J^{2}}\sum_{j=1}^{J}\Phi_{2}(P_{j}){\left[\frac{\sum_{k=1}^{N(P_{j})}m_{k}}{\sum_{k=1}^{N(P_{j})}m_{k}-1}\right]}^{2}G{\left(P_{j}\right)}^{2}, \end{array}$$

the covariances are

$$\begin{array}{l} Cov[{\hat{F}}_{4}(A,B;C,D),\overset{⌣}{G}(A)]\approx \frac{1}{J^{2}}\sum_{j=1}^{J}\frac{\Phi_{2}(A_{j})\sum_{k=1}^{N(A_{j})}m_{k}}{\sum_{k=1}^{N(A_{j})}m_{k}-1}G(A_{j})(1-2a_{j})(c_{j}-d_{j})\\ Cov[{\hat{F}}_{4}(A,B;C,D),\overset{⌣}{G}(B)]\approx -\frac{1}{J^{2}}\sum_{j=1}^{J}\frac{\Phi_{2}(B_{j})\sum_{k=1}^{N(B_{j})}m_{k}}{\sum_{k=1}^{N(B_{j})}m_{k}-1}G(B_{j})(1-2b_{j})(c_{j}-d_{j})\\ Cov[{\hat{F}}_{4}(A,B;C,D),\overset{⌣}{G}(C)]\approx \frac{1}{J^{2}}\sum_{j=1}^{J}\frac{\Phi_{2}(C_{j})\sum_{k=1}^{N(C_{j})}m_{k}}{\sum_{k=1}^{N(C_{j})}m_{k}-1}G(C_{j})(1-2c_{j})(a_{j}-b_{j})\\ Cov[{\hat{F}}_{4}(A,B;C,D),\overset{⌣}{G}(D)]\approx -\frac{1}{J^{2}}\sum_{j=1}^{J}\frac{\Phi_{2}(D_{j})\sum_{k=1}^{N(D_{j})}m_{k}}{\sum_{k=1}^{N(D_{j})}m_{k}-1}G(D_{j})(1-2d_{j})(a_{j}-b_{j}), \end{array}$$

and where

$$Y(P)=\frac{1}{J}\sum_{j=1}^{J}\frac{[1-\Phi_{2}(P_{j})]\sum_{k=1}^{N(P_{j})}m_{k}}{\sum_{k=1}^{N(P_{j})}m_{k}-1}G(P_{j}).$$

*Proof*. Recall that

$${\overset{⌣}{F}}_{4}(A,B;C,D|P)=\frac{{\hat{F}}_{4}(A,B;C,D)}{\overset{⌣}{G}(P)},$$

where ${\hat{F}}_{4}(A,B;C,D)$ is an unbiased estimator for $F_{4}(A,B;C,D)$ and $\overset{⌣}{G}(P)$ is an estimator of *G*(*P*). Assuming that $X={\hat{F}}_{4}(A,B;C,D)$ and $Y=\overset{⌣}{G}(P)$, following the approximation in Wolter (2007) we have

$$\begin{array}{l} Var[{\overset{⌣}{F}}_{4}(A,B;C,D|P)]\approx \frac{F_{4}{\left(A,B;C,D\right)}^{2}}{Y{\left(P\right)}^{2}}[\frac{\mathrm{Var}[{\hat{F}}_{4}(A,B;C,D)]}{F_{4}{\left(A,B;C,D\right)}^{2}}+\frac{\mathrm{Var}[\overset{⌣}{G}(A)]}{Y{\left(P\right)}^{2}}\\ -2\frac{\mathrm{Cov}[{\hat{F}}_{4}(A,B;C,D),\overset{⌣}{G}(P)]}{F_{4}(A,B;C,D)Y(P)}], \end{array}$$

where $\mathrm{Var}[{\hat{F}}_{4}(A,B;C,D)]$ is given in Proposition 27, $\mathrm{Var}[\overset{⌣}{G}(P)]$ in Lemma 18, $\mathrm{Cov}[{\hat{F}}_{4}(A,B;C,D),\overset{⌣}{G}(P)]$ in Lemma 36, and $Y(P)=E[\overset{⌣}{G}(P)]$ in proof of Corollary 11 for each population P∈{A,B,C,D}. □

Lemma 39. Consider *J* independent polymorphic loci in populations *A*, *B*, *C*, and *D* with respective parametric reference allele frequencies $a_{j},b_{j},c_{j},d_{j}\in (0,1)$, and suppose we take a random sample of $N(P_{j})$ individuals at locus *j* in population P∈{A,B,C,D}, some of which may be related or inbred. Moreover, assume that no individual is related to more than one other individual, which makes the terms $\Phi_{3}(P_{j}),\;\Phi_{4}(P_{j}),\;\Phi_{2,2}(P_{j}),\;\Phi_{2}{\left(P_{j}\right)}^{2},\;\Phi_{2}(A_{j})\Phi_{2}(B_{j}),\;\Phi_{2}(A_{j})\Phi_{2}(C_{j}),\;\Phi_{2}(A_{j})\Phi_{2}(D_{j}),\;\Phi_{2}(B_{j})\Phi_{2}(C_{j}),\;\Phi_{2}(B_{j})\Phi_{2}(D_{j})$, and $\Phi_{2}(C_{j})\Phi_{2}(D_{j})$ negligible to $\Phi_{2}(P_{j})$. Based on this simplifying assumption, the unbiased estimator $\hat{H}(A,B,C,D)$ has approximate variance

$$\begin{array}{l} Var[\hat{H}(A,B;C,D)]\approx \frac{1}{J^{2}}\sum_{j=1}^{J}{\left(a_{j}+b_{j}-2a_{j}b_{j}\right)}^{2}[\Phi_{2}(C_{j})G(C_{j}){\left(1-2d_{j}\right)}^{2}+\Phi_{2}(D_{j})G(D_{j}){\left(1-2c_{j}\right)}^{2}]\\ +\frac{1}{J^{2}}\sum_{j=1}^{J}{\left(c_{j}+d_{j}-2c_{j}d_{j}\right)}^{2}[\Phi_{2}(A_{j})G(A_{j}){\left(1-2b_{j}\right)}^{2}+\Phi_{2}(B_{j})G(B_{j}){\left(1-2a_{j}\right)}^{2}]. \end{array}$$

*Proof*. From the proof of Lemma 17, we have that

$$E[\hat{H}(A_{j},B_{j},C_{j},D_{j})]=H(A_{j},B_{j},C_{j},D_{j}),$$

yielding

$$E{\left[\hat{H}(A_{j},B_{j},C_{j},D_{j})\right]}^{2}=H{\left(A_{j},B_{j},C_{j},D_{j}\right)}^{2}.$$

We first calculate

$$\begin{array}{l} E[\hat{H}{\left(A_{j},B_{j},C_{j},D_{j}\right)}^{2}]=E[{\left({\hat{a}}_{j}+{\hat{b}}_{j}-2{\hat{a}}_{j}{\hat{b}}_{j}\right)}^{2}{\left({\hat{c}}_{j}+{\hat{d}}_{j}-2{\hat{c}}_{j}{\hat{d}}_{j}\right)}^{2}]\\ =E[{\left({\hat{a}}_{j}+{\hat{b}}_{j}-2{\hat{a}}_{j}{\hat{b}}_{j}\right)}^{2}]E[{\left({\hat{c}}_{j}+{\hat{d}}_{j}-2{\hat{c}}_{j}{\hat{d}}_{j}\right)}^{2}]. \end{array}$$

We compute the first term as

$$\begin{array}{l} E[{\left({\hat{a}}_{j}+{\hat{b}}_{j}-2{\hat{a}}_{j}{\hat{b}}_{j}\right)}^{2}]=E[{\hat{a}}_{j}^{2}]+E[{\hat{b}}_{j}^{2}]+4E[{\hat{a}}_{j}^{2}]E[{\hat{b}}_{j}^{2}]+2E[{\hat{a}}_{j}]E[{\hat{b}}_{j}]-4E[{\hat{a}}_{j}^{2}]E[{\hat{b}}_{j}]-4E[{\hat{a}}_{j}]E[{\hat{b}}_{j}^{2}]\\ =E[{\hat{a}}_{j}^{2}]+E[{\hat{b}}_{j}^{2}]+4E[{\hat{a}}_{j}^{2}]E[{\hat{b}}_{j}^{2}]+2a_{j}b_{j}-4E[{\hat{a}}_{j}^{2}]b_{j}-4a_{j}E[{\hat{b}}_{j}^{2}]\\ \approx a_{j}^{2}+\Phi_{2}(A_{j})a_{j}(1-a_{j})+b_{j}^{2}+\Phi_{2}(B_{j})b_{j}(1-b_{j})\\ +4[a_{j}^{2}+\Phi_{2}(A_{j})a_{j}(1-a_{j})][b_{j}^{2}+\Phi_{2}(B_{j})b_{j}(1-b_{j})]+2a_{j}b_{j}\\ -4[a_{j}^{2}+\Phi_{2}(A_{j})a_{j}(1-a_{j})]b_{j}-4a_{j}[b_{j}^{2}+\Phi_{2}(B_{j})b_{j}(1-b_{j})]\\ =a_{j}^{2}+b_{j}^{2}+4a_{j}^{2}b_{j}^{2}+2a_{j}b_{j}-4a_{j}^{2}b_{j}-4a_{j}b_{j}^{2}+\Phi_{2}(A_{j})a_{j}(1-a_{j})+\Phi_{2}(B_{j})b_{j}(1-b_{j})\\ +4\Phi_{2}(A_{j})a_{j}(1-a_{j})b_{j}^{2}+4\Phi_{2}(B_{j})a_{j}^{2}b_{j}(1-b_{j})+4\Phi_{2}(A_{j})\Phi_{2}(B_{j})a_{j}(1-a_{j})b_{j}(1-b_{j})\\ -4\Phi_{2}(A_{j})a_{j}(1-a_{j})b_{j}-4\Phi_{2}(B_{j})a_{j}b_{j}(1-b_{j})\\ ={\left(a_{j}+b_{j}-2a_{j}b_{j}\right)}^{2}+\Phi_{2}(A_{j})G(A_{j})[1-4b_{j}+4b_{j}^{2}]+\Phi_{2}(B_{j})G(B_{j})[1-4a_{j}+4a_{j}^{2}]\\ +4\Phi_{2}(A_{j})\Phi_{2}(B_{j})G(A_{j})G(B_{j})\\ \approx {\left(a_{j}+b_{j}-2a_{j}b_{j}\right)}^{2}+\Phi_{2}(A_{j})G(A_{j}){\left(1-2b_{j}\right)}^{2}+\Phi_{2}(B_{j})G(B_{j}){\left(1-2a_{j}\right)}^{2}, \end{array}$$

where we used the fact that $\Phi_{2}(A_{j})\Phi_{2}(B_{j})$ is negligible compared to $\Phi_{2}(A_{j})$ and $\Phi_{2}(B_{j})$ as an approximation. Using a similar argument, we have that

$$E[{\left({\hat{c}}_{j}+{\hat{d}}_{j}-2{\hat{c}}_{j}{\hat{d}}_{j}\right)}^{2}]\approx {\left(c_{j}+d_{j}-2c_{j}d_{j}\right)}^{2}+\Phi_{2}(C_{j})G(C_{j}){\left(1-2d_{j}\right)}^{2}+\Phi_{2}(D_{j})G(D_{j}){\left(1-2c_{j}\right)}^{2}.$$

Hence, we have that

$$\begin{array}{l} E[\hat{H}{\left(A_{j},B_{j},C_{j},D_{j}\right)}^{2}]=E[{\left({\hat{a}}_{j}+{\hat{b}}_{j}-2{\hat{a}}_{j}{\hat{b}}_{j}\right)}^{2}]E[{\left({\hat{c}}_{j}+{\hat{d}}_{j}-2{\hat{c}}_{j}{\hat{d}}_{j}\right)}^{2}]\\ \approx [{\left(a_{j}+b_{j}-2a_{j}b_{j}\right)}^{2}+\Phi_{2}(A_{j})G(A_{j}){\left(1-2b_{j}\right)}^{2}+\Phi_{2}(B_{j})G(B_{j}){\left(1-2a_{j}\right)}^{2}]\\ \times [{\left(c_{j}+d_{j}-2c_{j}d_{j}\right)}^{2}+\Phi_{2}(C_{j})G(C_{j}){\left(1-2d_{j}\right)}^{2}+\Phi_{2}(D_{j})G(D_{j}){\left(1-2c_{j}\right)}^{2}]\\ \approx H{\left(A_{j},B_{j},C_{j},D_{j}\right)}^{2}\\ +{\left(a_{j}+b_{j}-2a_{j}b_{j}\right)}^{2}[\Phi_{2}(C_{j})G(C_{j}){\left(1-2d_{j}\right)}^{2}+\Phi_{2}(D_{j})G(D_{j}){\left(1-2c_{j}\right)}^{2}]\\ +{\left(c_{j}+d_{j}-2c_{j}d_{j}\right)}^{2}[\Phi_{2}(A_{j})G(A_{j}){\left(1-2b_{j}\right)}^{2}+\Phi_{2}(B_{j})G(B_{j}){\left(1-2a_{j}\right)}^{2}], \end{array}$$

where we used the fact that $\Phi_{2}(A_{j})\Phi_{2}(C_{j}),\;\Phi_{2}(A_{j})\Phi_{2}(D_{j}),\;\Phi_{2}(B_{j})\Phi_{2}(C_{j})$, and $\Phi_{2}(B_{j})\Phi_{2}(D_{j})$ are negligible compared to $\Phi_{2}(A_{j}),\;\Phi_{2}(B_{j}),\;\Phi_{2}(C_{j})$, and $\Phi_{2}(D_{j})$ as an approximation. Putting it together, we have

$$\begin{array}{l} Var[\hat{H}(A_{j},B_{j},C_{j},D_{j})]=E[\hat{H}{\left(A_{j},B_{j},C_{j},D_{j}\right)}^{2}]-E{\left[\hat{H}(A_{j},B_{j},C_{j},D_{j})\right]}^{2}\\ =E[\hat{H}{\left(A_{j},B_{j},C_{j},D_{j}\right)}^{2}]-H{\left(A_{j},B_{j},C_{j},D_{j}\right)}^{2}\\ \approx {\left(a_{j}+b_{j}-2a_{j}b_{j}\right)}^{2}[\Phi_{2}(C_{j})G(C_{j}){\left(1-2d_{j}\right)}^{2}+\Phi_{2}(D_{j})G(D_{j}){\left(1-2c_{j}\right)}^{2}]\\ +{\left(c_{j}+d_{j}-2c_{j}d_{j}\right)}^{2}[\Phi_{2}(A_{j})G(A_{j}){\left(1-2b_{j}\right)}^{2}+\Phi_{2}(B_{j})G(B_{j}){\left(1-2a_{j}\right)}^{2}]. \end{array}$$

Given the assumption of independent loci, we have

$$\begin{array}{l} Var[\hat{H}(A,B,C,D)]=\mathrm{Var}[\frac{1}{J}\sum_{j=1}^{J}\hat{H}(A_{j},B_{j},C_{j},D_{j})]\\ =\frac{1}{J^{2}}\sum_{j=1}^{J}Var[\hat{H}(A_{j},B_{j},C_{j},D_{j})]\\ \approx \frac{1}{J^{2}}\sum_{j=1}^{J}{\left(a_{j}+b_{j}-2a_{j}b_{j}\right)}^{2}[\Phi_{2}(C_{j})G(C_{j}){\left(1-2d_{j}\right)}^{2}+\Phi_{2}(D_{j})G(D_{j}){\left(1-2c_{j}\right)}^{2}]\\ +\frac{1}{J^{2}}\sum_{j=1}^{J}{\left(c_{j}+d_{j}-2c_{j}d_{j}\right)}^{2}[\Phi_{2}(A_{j})G(A_{j}){\left(1-2b_{j}\right)}^{2}+\Phi_{2}(B_{j})G(B_{j}){\left(1-2a_{j}\right)}^{2}].\;□ \end{array}$$

Lemma 40. Consider *J* independent polymorphic loci in populations *A*, *B*, *C*, and *D* with respective parametric reference allele frequencies $a_{j},b_{j},c_{j},d_{j}\in (0,1)$, and suppose we take a random sample of $N(P_{j})$ individuals at locus *j* in population P∈{A,B,C,D}, some of which may be related or inbred. Moreover, assume that no individual is related to more than one other individual, which makes the terms $\Phi_{3}(P_{j}),\;\Phi_{4}(P_{j}),\;\Phi_{2,2}(P_{j}),\;\Phi_{2}{\left(P_{j}\right)}^{2},\;\Phi_{2}(A_{j})\Phi_{2}(C_{j}),\;\Phi_{2}(A_{j})\Phi_{2}(D_{j}),\;\Phi_{2}(B_{j})\Phi_{2}(C_{j})$, and $\Phi_{2}(B_{j})\Phi_{2}(D_{j})$ negligible to $\Phi_{2}(P_{j})$. Based on this simplifying assumption, the unbiased estimators ${\hat{F}}_{4}(A,B;C,D)$ and $\hat{H}(A,B,C,D)$ have approximate covariance

$$\begin{array}{l} Cov[{\hat{F}}_{4}(A,B;C,D),\hat{H}(A,B,C,D)]\approx \frac{1}{J^{2}}\sum_{j=1}^{J}\Phi_{2}(A_{j})G(A_{j})(c_{j}-d_{j})(c_{j}+d_{j}-2c_{j}d_{j})(1-2b_{j})\\ -\frac{1}{J^{2}}\sum_{j=1}^{J}\Phi_{2}(B_{j})G(B_{j})(c_{j}-d_{j})(c_{j}+d_{j}-2c_{j}d_{j})(1-2a_{j})\\ +\frac{1}{J^{2}}\sum_{j=1}^{J}\Phi_{2}(C_{j})G(C_{j})(a_{j}-b_{j})(a_{j}+b_{j}-2a_{j}b_{j})(1-2d_{j})\\ -\frac{1}{J^{2}}\sum_{j=1}^{J}\Phi_{2}(D_{j})G(D_{j})(a_{j}-b_{j})(a_{j}+b_{j}-2a_{j}b_{j})(1-2c_{j}). \end{array}$$

*Proof*. From the proofs of Proposition 12 and Lemma 17, we that have

$$E[{\hat{F}}_{4}(A_{j},B_{j};C_{j},D_{j})]=F_{4}(A_{j},B_{j};C_{j},D_{j}),$$

and

$$E[\hat{H}(A_{j},B_{j},C_{j},D_{j})]=H(A_{j},B_{j},C_{j},D_{j}),$$

yielding

$$E[{\hat{F}}_{4}(A_{j},B_{j};C_{j},D_{j})]E[\hat{H}(A_{j},B_{j},C_{j},D_{j})]=F_{4}(A_{j},B_{j};C_{j},D_{j})H(A_{j},B_{j},C_{j},D_{j}).$$

We first calculate

$$\begin{array}{l} E[{\hat{F}}_{4}(A_{j},B_{j};C_{j},D_{j})\hat{H}(A_{j},B_{j},C_{j},D_{j})]=E[({\hat{a}}_{j}-{\hat{b}}_{j})({\hat{c}}_{j}-{\hat{d}}_{j})({\hat{a}}_{j}+{\hat{b}}_{j}-2{\hat{a}}_{j}{\hat{b}}_{j})({\hat{c}}_{j}+{\hat{d}}_{j}-2{\hat{c}}_{j}{\hat{d}}_{j})]\\ =E[({\hat{a}}_{j}-{\hat{b}}_{j})({\hat{a}}_{j}+{\hat{b}}_{j}-2{\hat{a}}_{j}{\hat{b}}_{j})]E[({\hat{c}}_{j}-{\hat{d}}_{j})({\hat{c}}_{j}+{\hat{d}}_{j}-2{\hat{c}}_{j}{\hat{d}}_{j})]. \end{array}$$

We compute the first term as

$$\begin{array}{l} E[({\hat{a}}_{j}-{\hat{b}}_{j})({\hat{a}}_{j}+{\hat{b}}_{j}-2{\hat{a}}_{j}{\hat{b}}_{j})]=E[{\hat{a}}_{j}^{2}]-2E[{\hat{a}}_{j}^{2}]E[{\hat{b}}_{j}]-E[{\hat{b}}_{j}^{2}]+2E[{\hat{a}}_{j}]E[{\hat{b}}_{j}^{2}]\\ =E[{\hat{a}}_{j}^{2}](1-2E[{\hat{b}}_{j}])-E[{\hat{b}}_{j}^{2}](1-2E[{\hat{a}}_{j}])\\ \approx [a_{j}^{2}+\Phi_{2}(A_{j})a_{j}(1-a_{j})][1-2b_{j}]-[b_{j}^{2}+\Phi_{2}(B_{j})b_{j}(1-b_{j})][1-2a_{j}]\\ =(a_{j}-b_{j})(a_{j}+b_{j}-2a_{j}b_{j})+\Phi_{2}(A_{j})G(A_{j})(1-2b_{j})-\Phi_{2}(B_{j})G(B_{j})(1-2a_{j}). \end{array}$$

Using a similar argument, we have that

$$E[({\hat{c}}_{j}-{\hat{d}}_{j})({\hat{c}}_{j}+{\hat{d}}_{j}-2{\hat{c}}_{j}{\hat{d}}_{j})]\approx (c_{j}-d_{j})(c_{j}+d_{j}-2c_{j}d_{j})+\Phi_{2}(C_{j})g(C_{j})(1-2d_{j})-\Phi_{2}(D_{j})g(D_{j})(1-2c_{j}).$$

Hence, we have that

$$\begin{array}{l} E[{\hat{F}}_{4}(A_{j},B_{j};C_{j},D_{j})\hat{H}(A_{j},B_{j},C_{j},D_{j})]=E[({\hat{a}}_{j}-{\hat{b}}_{j})({\hat{a}}_{j}+{\hat{b}}_{j}-2{\hat{a}}_{j}{\hat{b}}_{j})]E[({\hat{c}}_{j}-{\hat{d}}_{j})({\hat{c}}_{j}+{\hat{d}}_{j}-2{\hat{c}}_{j}{\hat{d}}_{j})]\\ \approx [(a_{j}-b_{j})(a_{j}+b_{j}-2a_{j}b_{j})+\Phi_{2}(A_{j})G(A_{j})(1-2b_{j})\\ -\Phi_{2}(B_{j})G(B_{j})(1-2a_{j})]\\ \times [(c_{j}-d_{j})(c_{j}+d_{j}-2c_{j}d_{j})+\Phi_{2}(C_{j})G(C_{j})(1-2d_{j})\\ -\Phi_{2}(D_{j})G(D_{j})(1-2c_{j})]\\ \approx F_{4}(A_{j},B_{j};C_{j},D_{j})H(A_{j},B_{j},C_{j},D_{j})\\ +\Phi_{2}(A_{j})G(A_{j})(c_{j}-d_{j})(c_{j}+d_{j}-2c_{j}d_{j})(1-2b_{j})\\ -\Phi_{2}(B_{j})G(B_{j})(c_{j}-d_{j})(c_{j}+d_{j}-2c_{j}d_{j})(1-2a_{j})\\ +\Phi_{2}(C_{j})G(C_{j})(a_{j}-b_{j})(a_{j}+b_{j}-2a_{j}b_{j})(1-2d_{j})\\ -\Phi_{2}(D_{j})G(D_{j})(a_{j}-b_{j})(a_{j}+b_{j}-2a_{j}b_{j})(1-2c_{j}), \end{array}$$

where we used the fact that $\Phi_{2}(A_{j})\Phi_{2}(C_{j}),\;\Phi_{2}(A_{j})\Phi_{2}(D_{j}),\;\Phi_{2}(B_{j})\Phi_{2}(C_{j})$, and $\Phi_{2}(B_{j})\Phi_{2}(D_{j})$ are negligible compared to $\Phi_{2}(A_{j}),\;\Phi_{2}(B_{j}),\;\Phi_{2}(C_{j})$, and $\Phi_{2}(D_{j})$ as an approximation. Putting it together, we have

$$\begin{array}{l} Cov[{\hat{F}}_{4}(A_{j},B_{j};C_{j},D_{j}),\hat{H}(A_{j},B_{j},C_{j},D_{j})]=E[{\hat{F}}_{4}(A_{j},B_{j};C_{j},D_{j})\hat{H}(A_{j},B_{j},C_{j},D_{j})]\\ -E[{\hat{F}}_{4}(A_{j},B_{j};C_{j},D_{j})]E[\hat{H}(A_{j},B_{j},C_{j},D_{j})]\\ =E[{\hat{F}}_{4}(A_{j},B_{j};C_{j},D_{j})\hat{H}(A_{j},B_{j},C_{j},D_{j})]\\ -F_{4}(A_{j},B_{j};C_{j},D_{j})H(A_{j},B_{j},C_{j},D_{j})\\ \approx \Phi_{2}(A_{j})G(A_{j})(c_{j}-d_{j})(c_{j}+d_{j}-2c_{j}d_{j})(1-2b_{j})\\ -\Phi_{2}(B_{j})G(B_{j})(c_{j}-d_{j})(c_{j}+d_{j}-2c_{j}d_{j})(1-2a_{j})\\ +\Phi_{2}(C_{j})G(C_{j})(a_{j}-b_{j})(a_{j}+b_{j}-2a_{j}b_{j})(1-2d_{j})\\ -\Phi_{2}(D_{j})G(D_{j})(a_{j}-b_{j})(a_{j}+b_{j}-2a_{j}b_{j})(1-2c_{j}). \end{array}$$

Given the assumption of independent loci, we have

$$\begin{array}{l} Cov[{\hat{F}}_{4}(A,B;C,D),\hat{H}(A,B,C,D)]=\mathrm{Cov}[\frac{1}{J}\sum_{j=1}^{J}{\hat{F}}_{4}(A_{j},B_{j};C_{j},D_{j}),\frac{1}{J}\sum_{j=1}^{J}\hat{H}(A_{j},B_{j},C_{j},D_{j})]\\ =\frac{1}{J^{2}}\sum_{j=1}^{J}Cov[{\hat{F}}_{4}(A_{j},B_{j};C_{j},D_{j}),\hat{H}(A_{j},B_{j},C_{j},D_{j})]\\ \approx \frac{1}{J^{2}}\sum_{j=1}^{J}\Phi_{2}(A_{j})G(A_{j})(c_{j}-d_{j})(c_{j}+d_{j}-2c_{j}d_{j})(1-2b_{j})\\ -\frac{1}{J^{2}}\sum_{j=1}^{J}\Phi_{2}(B_{j})G(B_{j})(c_{j}-d_{j})(c_{j}+d_{j}-2c_{j}d_{j})(1-2a_{j})\\ +\frac{1}{J^{2}}\sum_{j=1}^{J}\Phi_{2}(C_{j})G(C_{j})(a_{j}-b_{j})(a_{j}+b_{j}-2a_{j}b_{j})(1-2d_{j})\\ -\frac{1}{J^{2}}\sum_{j=1}^{J}\Phi_{2}(D_{j})G(D_{j})(a_{j}-b_{j})(a_{j}+b_{j}-2a_{j}b_{j})(1-2c_{j}).\;□ \end{array}$$

**Proposition 41.** Consider *J* polymorphic loci in populations *A*, *B*, *C*, and *D* with respective parametric reference allele frequencies $a_{j},b_{j},c_{j},d_{j}\in (0,1)$, and suppose we take a random sample of $N(P_{j})$ individuals at locus *j* in population P∈{A,B,C,D}, some of which may be related or inbred. Moreover, assume that no individual is related to more than one other individual, which makes the terms $\Phi_{3}(P_{j}),\;\Phi_{4}(P_{j}),\;\Phi_{2,2}(P_{j}),\;\Phi_{2}{\left(P_{j}\right)}^{2},\;\Phi_{2}(A_{j})\Phi_{2}(B_{j}),\;\Phi_{2}(A_{j})\Phi_{2}(C_{j}),\;\Phi_{2}(A_{j})\Phi_{2}(D_{j}),\;\Phi_{2}(B_{j})\Phi_{2}(C_{j}),\;\Phi_{2}(B_{j})\Phi_{2}(D_{j})$, and $\Phi_{2}(C_{j})\Phi_{2}(D_{j})$ negligible to $\Phi_{2}(P_{j})$. Based on this simplifying assumption, the approximately unbiased ratio estimator $\hat{D}(A,B,C,D)$ has approximate variance

$$\begin{array}{l} Var[\hat{D}(A,B;C,D)]\approx \frac{F_{4}{\left(A,B;C,D\right)}^{2}}{H{\left(A,B,C,D\right)}^{2}}[\frac{\mathrm{Var}[{\hat{F}}_{4}(A,B;C,D)]}{F_{4}{\left(A,B;C,D\right)}^{2}}+\frac{\mathrm{Var}[\hat{H}(A,B,C,D)]}{H{\left(A,B,C,D\right)}^{2}}\\ -2\frac{\mathrm{Cov}[{\hat{F}}_{4}(A,B;C,D),\hat{H}(A,B,C,D)]}{F_{4}(A,B;C,D)H(A,B,C,D)}], \end{array}$$

where the variances are

$$\begin{array}{l} Var[{\hat{F}}_{4}(A,B;C,D)]\approx \frac{1}{J^{2}}\sum_{j=1}^{J}[\Phi_{2}(C_{j})G(C_{j})+\Phi_{2}(D_{j})G(D_{j})]F_{2}(A_{j},B_{j})\\ +\frac{1}{J^{2}}\sum_{j=1}^{J}[\Phi_{2}(A_{j})G(A_{j})+\Phi_{2}(B_{j})G(B_{j})]F_{2}(C_{j},D_{j})\\ Var[\hat{H}(A,B;C,D)]\approx \frac{1}{J^{2}}\sum_{j=1}^{J}{\left(a_{j}+b_{j}-2a_{j}b_{j}\right)}^{2}[\Phi_{2}(C_{j})G(C_{j}){\left(1-2d_{j}\right)}^{2}+\Phi_{2}(D_{j})G(D_{j}){\left(1-2c_{j}\right)}^{2}]\\ +\frac{1}{J^{2}}\sum_{j=1}^{J}{\left(c_{j}+d_{j}-2c_{j}d_{j}\right)}^{2}[\Phi_{2}(A_{j})G(A_{j}){\left(1-2b_{j}\right)}^{2}+\Phi_{2}(B_{j})G(B_{j}){\left(1-2a_{j}\right)}^{2}]. \end{array}$$

and the covariance is

$$\begin{array}{l} Cov[{\hat{F}}_{4}(A,B;C,D),\hat{H}(A,B,C,D)]\approx \frac{1}{J^{2}}\sum_{j=1}^{J}\Phi_{2}(A_{j})G(A_{j})(c_{j}-d_{j})(c_{j}+d_{j}-2c_{j}d_{j})(1-2b_{j})\\ -\frac{1}{J^{2}}\sum_{j=1}^{J}\Phi_{2}(B_{j})G(B_{j})(c_{j}-d_{j})(c_{j}+d_{j}-2c_{j}d_{j})(1-2a_{j})\\ +\frac{1}{J^{2}}\sum_{j=1}^{J}\Phi_{2}(C_{j})G(C_{j})(a_{j}-b_{j})(a_{j}+b_{j}-2a_{j}b_{j})(1-2d_{j})\\ -\frac{1}{J^{2}}\sum_{j=1}^{J}\Phi_{2}(D_{j})G(D_{j})(a_{j}-b_{j})(a_{j}+b_{j}-2a_{j}b_{j})(1-2c_{j}). \end{array}$$

*Proof*. Recall that

$$\hat{D}(A,B;C,D)=\frac{{\hat{F}}_{4}(A,B;C,D)}{\hat{H}(A,B,C,D)},$$

where ${\hat{F}}_{4}(A,B;C,D)$ is an unbiased estimator for $F_{4}(A,B;C,D)$ and $\hat{H}(A,B,C,D)$ is an unbiased estimator of H(A,B,C,D). Assuming that $X={\hat{F}}_{4}(A,B;C,D)$ and $Y=\hat{H}(A,B,C,D)$, following the approximation in Wolter (2007) we have

$$\begin{array}{l} Var[\tilde{D}(A,B;C,D)]\approx \frac{F_{4}{\left(A,B;C,D\right)}^{2}}{H{\left(A,B,C,D\right)}^{2}}[\frac{\mathrm{Var}[{\hat{F}}_{4}(A,B;C,D)]}{F_{4}{\left(A,B;C,D\right)}^{2}}+\frac{\mathrm{Var}[\hat{H}(A,B,C,D)]}{H{\left(A,B,C,D\right)}^{2}}\\ -2\frac{\mathrm{Cov}[{\hat{F}}_{4}(A,B;C,D),\hat{H}(A,B,C,D)]}{F_{4}(A,B;C,D)H(A,B,C,D)}], \end{array}$$

where $\mathrm{Var}[{\hat{F}}_{4}(A,B;C,D)]$ is given in Proposition 27, $\mathrm{Var}[\hat{H}(A,B,C,D)]$ in Lemma 39, and $\mathrm{Cov}[{\hat{F}}_{4}(A,B;C,D),\hat{H}(A,B,C,D)]$ in Lemma 40. □
