# Erratum to: Properties and unbiased estimation of F- and D-statistics in samples containing related and inbred individuals

**DOI:** 10.1093/genetics/iyab128

**Published:** 2021-12-08

*GENETICS* 2021, 1-14

An incorrect manuscript was inadvertently published under DOI 10.1093/genetics/iyab090 due to production error. The definitive uncorrected manuscript version of the above stated article has now been reinstated.

