# Supplementary material for: Properties and unbiased estimation of F- and D-statistics in samples containing related and inbred individuals
Source: Genetics. 2021 Jul 15;220(1):iyab090. doi: 10.1093/genetics/iyab090 (PMC8733448; doi:10.1093/genetics/iyab090)
Supplement: iyab090_Supplementary_Figures [file iyab090_supplementary_figures.pdf]

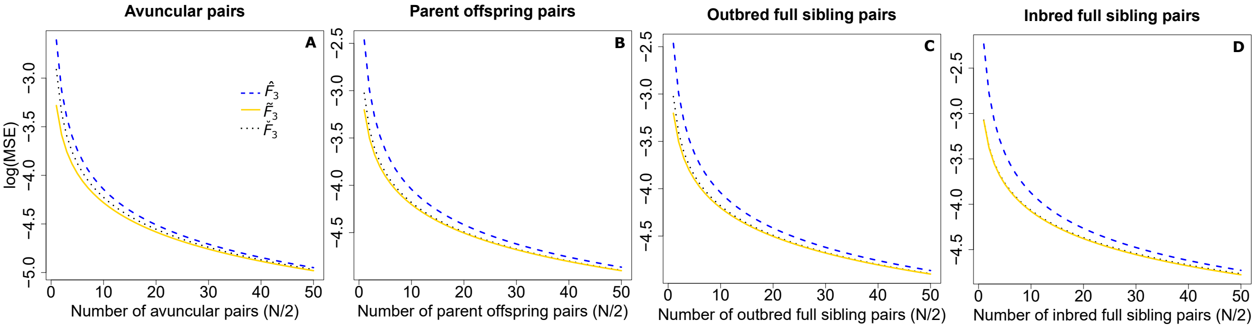

Figure S1: Mean squared error theoretically calculated for $\widehat{F_3}(A;B,C)$, $\breve{F_3}(A;B,C)$ and $\widetilde{F_3}(A;B,C)$ across different sample sizes or related pairs of individuals, including avuncular relationships (panel **A**), parent-offspring relationships (panel **B**), outbred full siblings (panel **C**), and inbred full siblings (panel **D**). The number of sampled individuals ranges from two to 100 with the number of relative pairs equaling half the total sampled, all computed using $J = 20$ loci. The true value of $F_3(A;B,C)$ is 0.033.

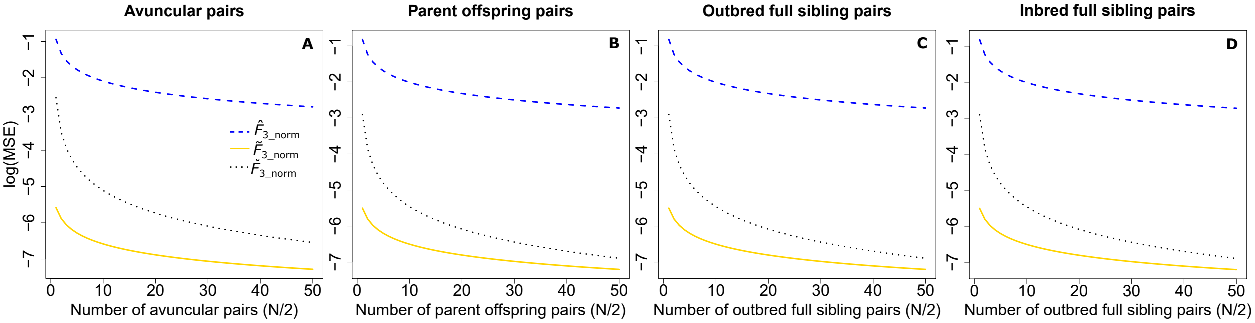

Figure S2: Mean squared error theoretically calculated for normalized $\widehat{F}_3(A; B, C \mid A)$, $\breve{F}_3(A; B, C \mid A)$, and $\widetilde{F}_3(A; B, C \mid A)$ across different sample sizes or related pairs of individuals, including avuncular relationships (panel **A**), parent-offspring relationships (panel **B**), outbred full siblings (panel **C**), and inbred full siblings (panel **D**). The number of sampled individuals ranges from two to 100 with the number of relative pairs equaling half the total sampled, all computed using $J = 20$ loci. The true value of normalized $F_3(A; B, C \mid A)$ is 0.116.

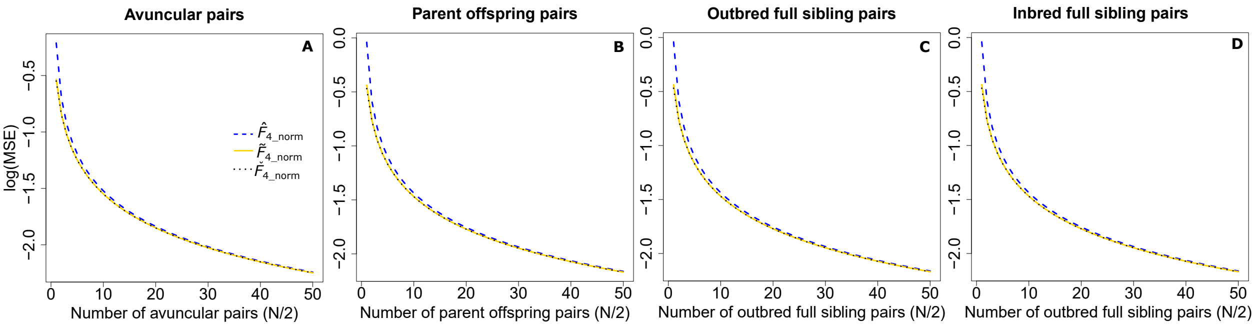

Figure S3: Mean squared error theoretically calculated for $\widehat{F}_4(A, B; C, D \mid A)$, $\breve{F}_4(A; B, C \mid A)$, and $\widetilde{F}_4(A, B; C, D \mid A)$ across different sample sizes or related pairs of individuals,including avuncular relationships (panel **A**), parent-offspring relationships (panel **B**), outbred full siblings (panel **C**), and inbred full siblings (panel **D**). The number of sampled individuals ranges from two to 100 with the number of relative pairs equaling half the total sampled, all computed using $J = 20$ loci. The true value of normalized $F_4(A, B; C, D \mid A)$ is 0.052.

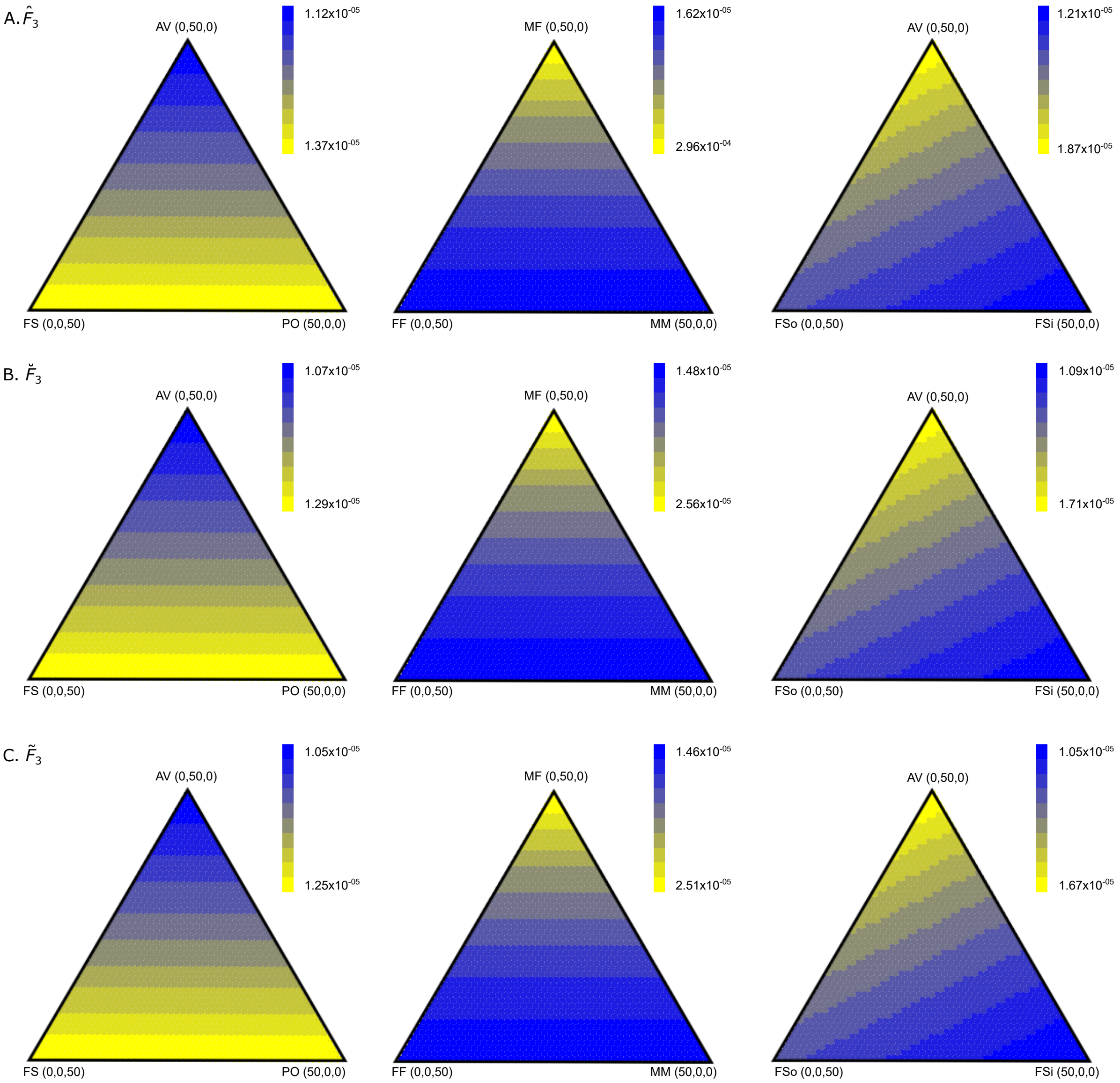

Figure S4: Theoretically calculated MSE of $\widehat{F}_3(A;B,C)$ (panel **A**), $\breve{F}_3(A;B,C)$ (panel **B**), and $\widetilde{F}_3(A;B,C)$ (panel **C**) when including relatives or inbred individuals for $J = 20$ loci. The MSE is estimated for instances when samples of 100 individuals include individuals related to exactly one other in the sample. The first column shows MSE for samples with different combinations of parent-offspring (PO), full sibling (FS), and avuncular (AV) relationships, the second includes full siblings that are male-male (MM), male-female (MF) and female-female (MF). The last column includes AV relationships as well as inbred (FSi) and outbred (FSo) full siblings. The true value of $F_3(A;B,C)$ is 0.033.

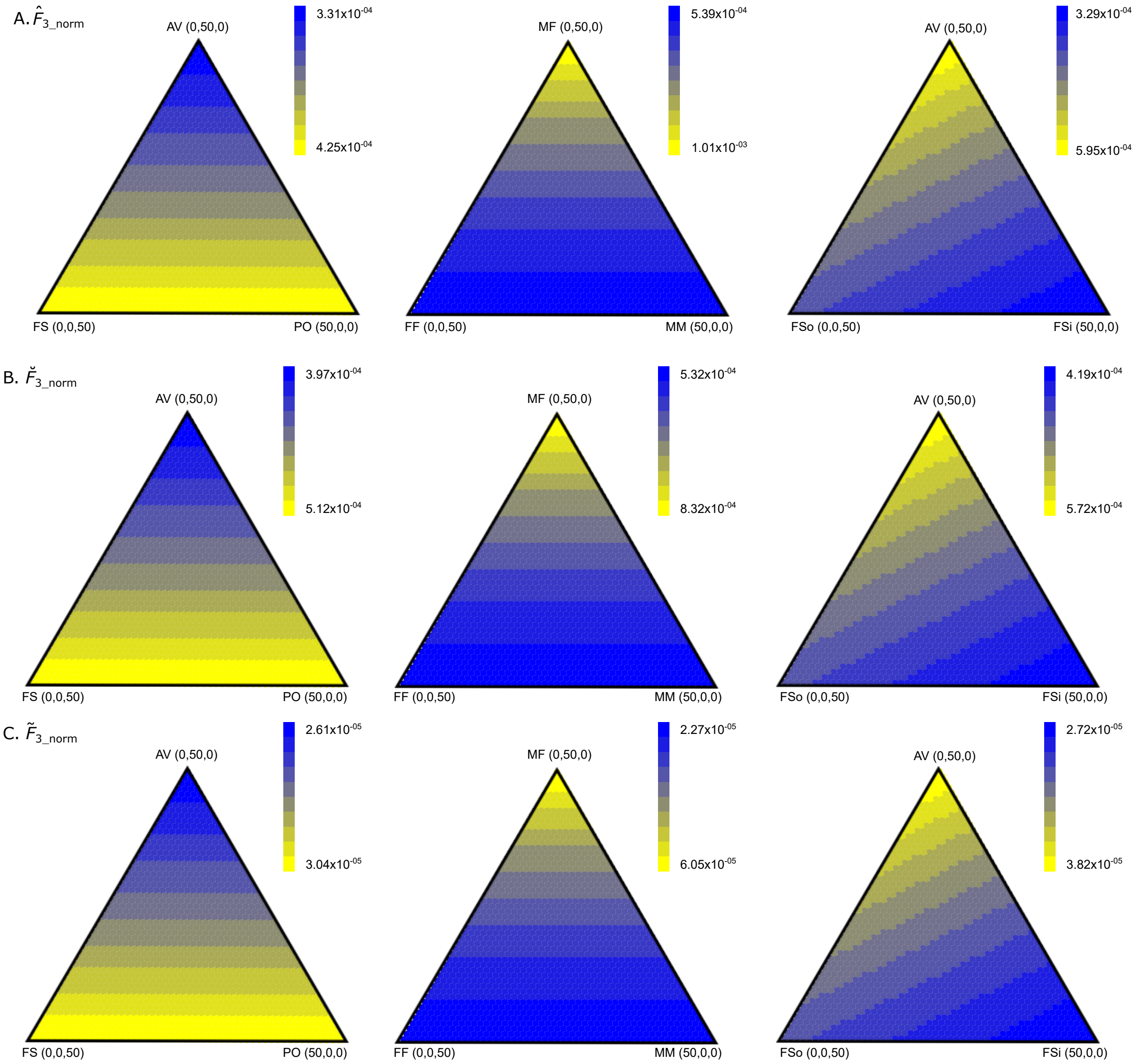

Figure S5: Theoretically calculated MSE $\widehat{F}_3(A;B,C\,|\,A)$ (panel **A**), $\breve{F}_3(A;B,C\,|\,A)$ (panel **B**), and normalized $\widetilde{F}_3(A;B,C\,|\,A)$ (panel **C**), when including relatives or inbred individuals for $J = 20$ loci. The MSE is estimated for instances when samples of 100 individuals include individuals related to exactly one other in the sample. The first column shows MSE for samples with different combinations of parent-offspring (PO), full sibling (FS), and avuncular (AV) relationships, the second includes full siblings that are male-male (MM), male-female (MF) and female-female (MF). The last column includes AV relationships as well as inbred (FSi) and outbred (FSo) full siblings. The true value of normalized $F_3(A;B,C\,|\,A)$ is 0.116.

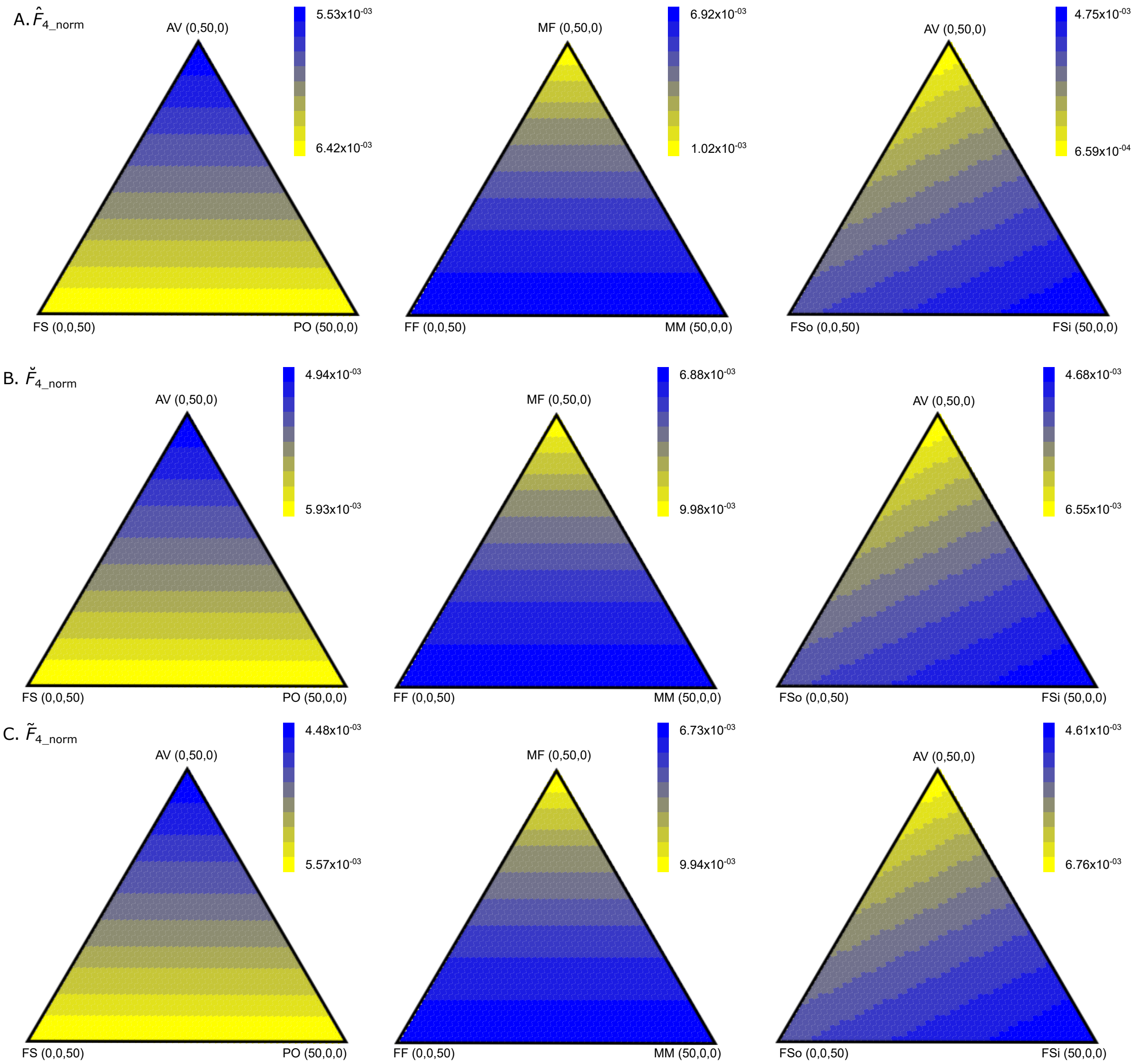

Figure S6: Theoretically calculated MSE of $\widehat{F}_4(A, B; C, D \mid A)$ (panel **A**), $\breve{F}_4(A, B; C, D \mid A)$ (panel **B**), and $\widetilde{F}_4(A, B; C, D \mid A)$ (panel **C**) when including relatives or inbred individuals for $J = 20$ loci. The MSE is estimated for instances when samples of 100 individuals include individuals related to exactly one other in the sample. The first column shows MSE for samples with different combinations of parent-offspring (PO), full sibling (FS), and avuncular (AV) relationships, the second includes full siblings that are male-male (MM), male-female (MF) and female-female (MF). The last column includes AV relationships as well as inbred (FSi) and outbred (FSo) full siblings. The true value of normalized $F_4(A, B; C, D \mid A)$ is 0.052.

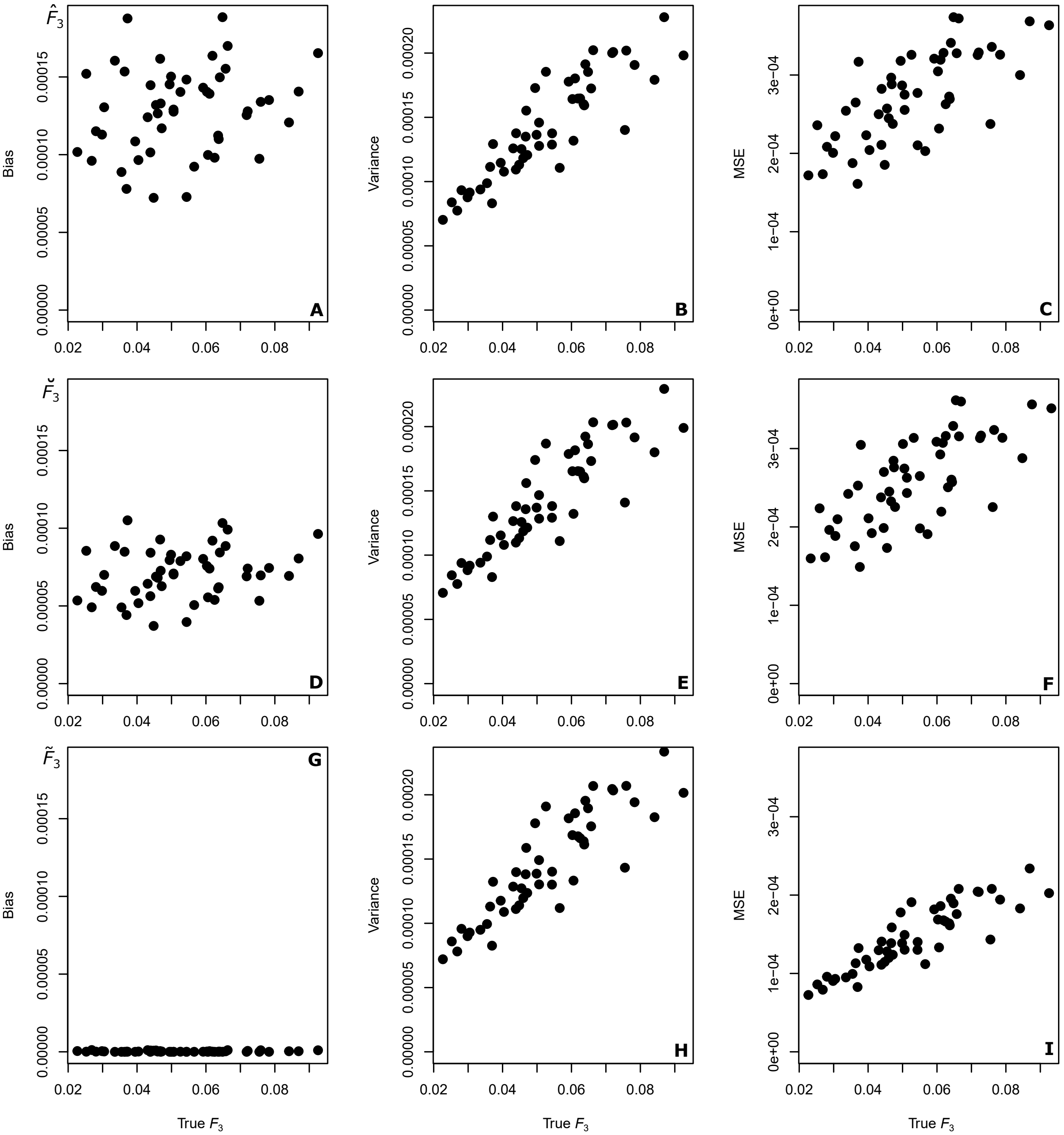

Figure S7: Comparison of squared bias (panels **A**, **D**, and **G**), variance (panels **B**, **E**, and **H**), and MSE (panels **C**, **F**, and **I**), for $\widehat{F}_3(A;B,C)$, $\breve{F}_3(A;B,C)$, and $\widetilde{F}_3(A;B,C)$ from simulated data including 60 parent offspring relative pairs. Each estimate was computed using $J = 20$ randomly sampled loci using $A =$ JPT, $B =$ CEU, and $C =$ YRI.

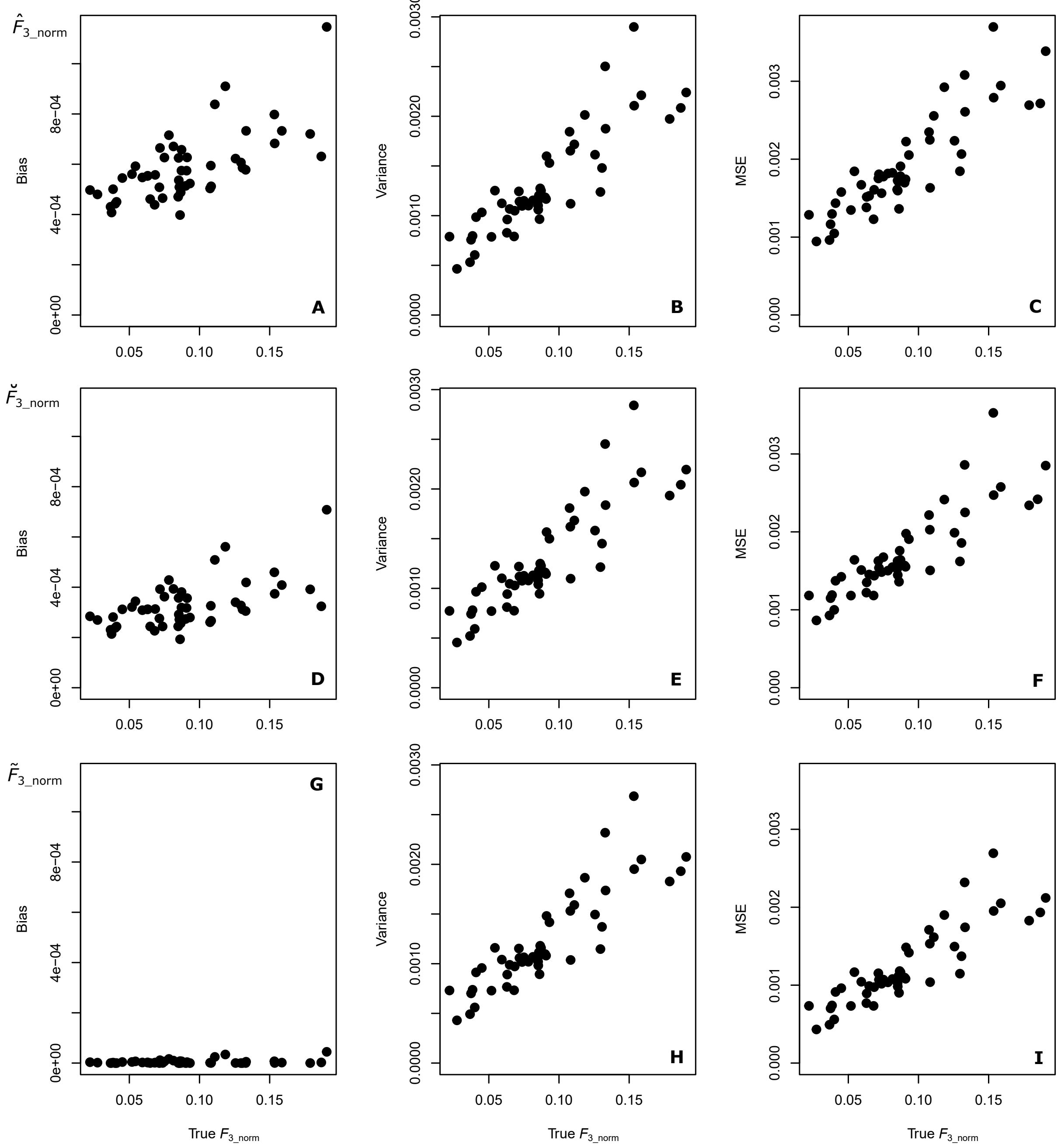

Figure S8: Comparison of squared bias (panels **A**, **D**, and **G**), variance (panels **B**, **E**, and **H**), and MSE (panels **C**, **F**, and **I**), for $\widehat{F}_3(A;B,C\,|\,A)$, $\breve{F}_3(A;B,C\,|\,A)$, and $\widetilde{F}_3(A;B,C\,|\,A)$ from simulated data including 60 relative pairs. Each estimate was computed using $J=20$ randomly sampled loci using $A=$ JPT, $B=$ CEU, and $C=$ YRI.

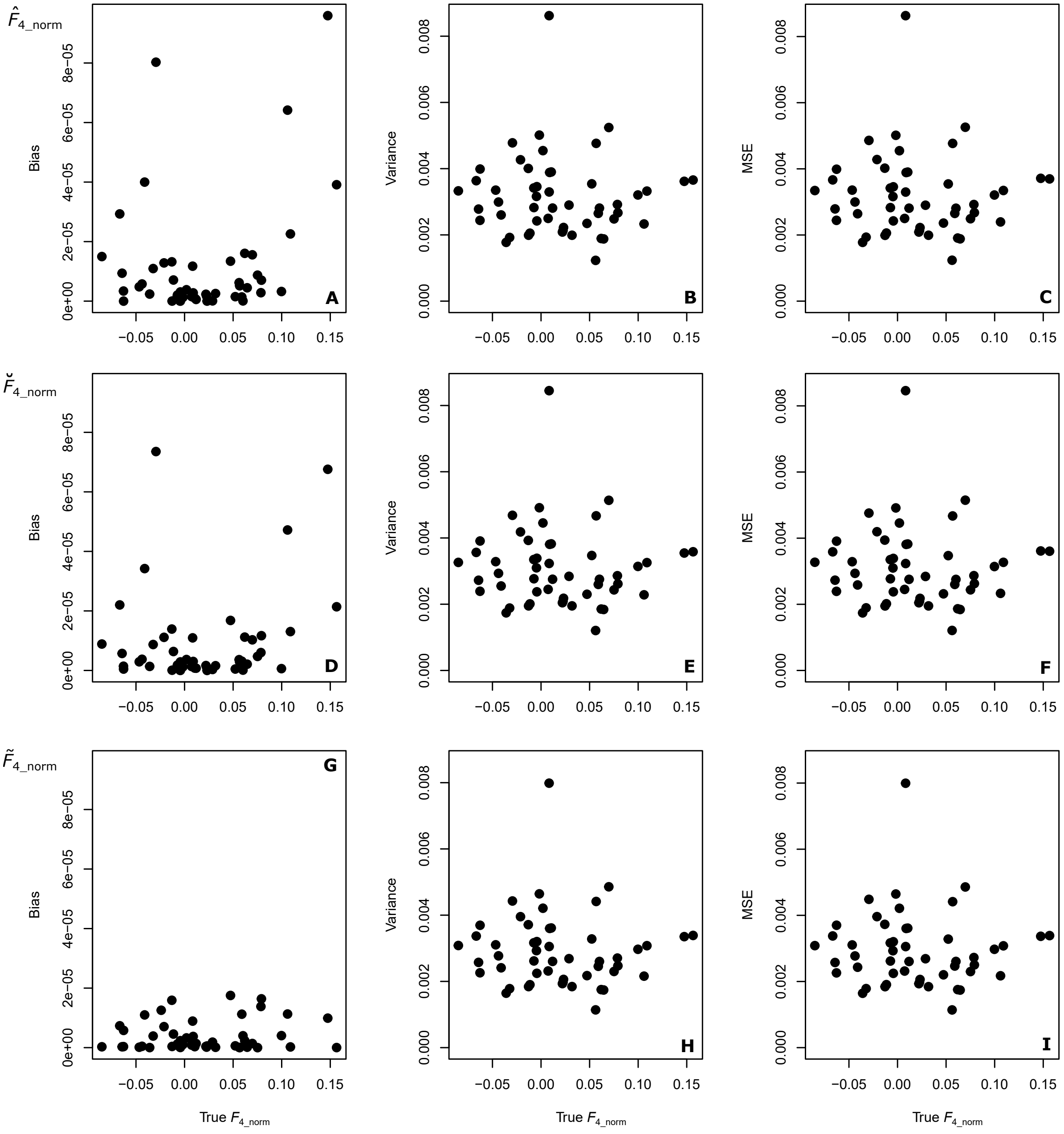

Figure S9: Comparison of squared bias (panels **A**, **D**, and **G**), variance (panels **B**, **E**, and **H**), and MSE (panels **C**, **F**, and **I**), for normalized $\widehat{F}_4(A, B; C, D \mid A)$, $\breve{F}_4(A, B; C, D \mid A)$, and $\widetilde{F}_4(A, B; C, D \mid A)$ from simulated data including 60 relative pairs. Each estimate was computed using $J = 20$ randomly sampled loci using $A =$ YRI, $B =$ CEU, $C =$ JPT, and $D =$ GIH.

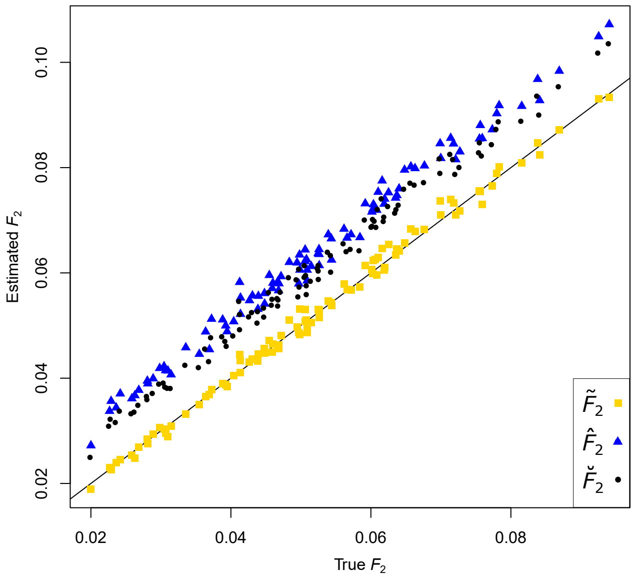

Figure S10: Comparison of true $F_2(A, B)$ to estimated $\widetilde{F}_2(A, B)$, $\breve{F}_2(A, B)$, and $\widehat{F}_2(A, B)$. Each dot represents the mean of 1000 simulations of parent offspring pairs used to compute $\widetilde{F}_2(A, B)$, $\breve{F}_2(A, B)$, and $\widehat{F}_2(A, B)$. Each simulation contains 50 parent offspring pairs.

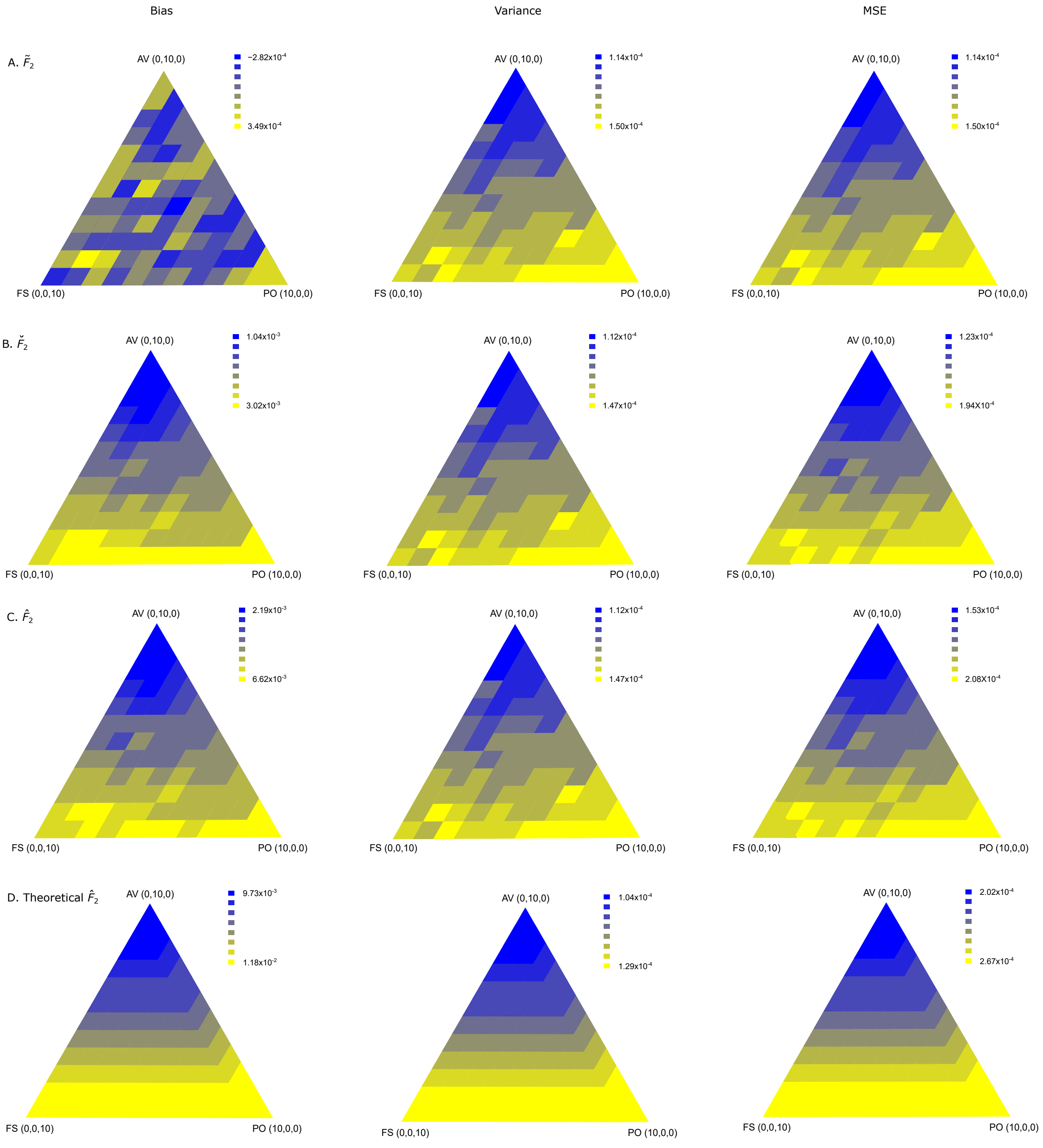

Figure S11: Theoretical vs. simulated $F_2(A, B)$ bias, variance, and MSE when including various combinations of parent-offspring, avuncular and outbred full-sibling pairs. (Row A) Simulations of relative pairs used to compute bias, variance, and MSE of $\widetilde{F}_2(A, B)$. (Row B) Simulations of relative pairs used to compute bias, variance, and MSE of Patterson's unbiased $\breve{F}_2(A, B)$. (Row C) Simulations of relative pairs used to compute bias, variance, and MSE of unbiased $\widehat{F}_2(A, B)$. (Row D) Theoretically computed bias, variance, and MSE for $\widehat{F}_2(A, B)$.The true value of $F_2(A, B)$ is 0.071, computed for $J = 20$ loci.

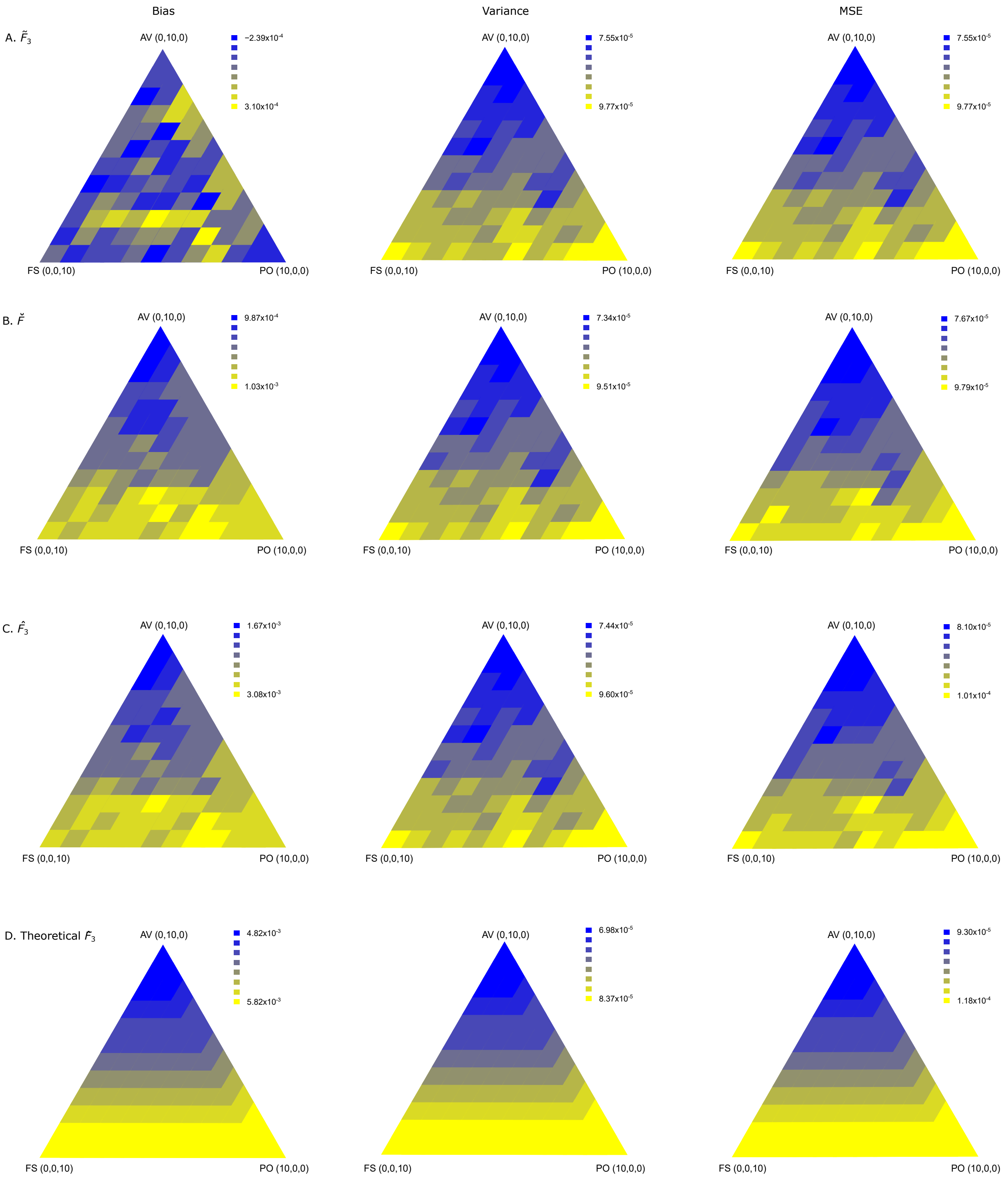

Figure S12: Theoretical vs. simulated $F_3(A;B,C)$ bias, variance, and MSE when including various combinations of parent-offspring, avuncular and outbred full-sibling pairs. (Row A) Simulations of relative pairs used to compute bias, variance, and MSE of $\widetilde{F}_3(A;B,C)$. (Row B) Simulations of relative pairs used to compute bias, variance, and MSE of $\breve{F}_3(A;B,C)$. (Row C) Simulations of relative pairs used to compute bias, variance, and MSE of $\widehat{F}_3(A;B,C)$. (Row D) Theoretically computed bias, variance, and MSE for $\widehat{F}_3(A;B,C)$. The true value of $F_3(A;B,C)$ is 0.033, computed for $J = 20$ loci.

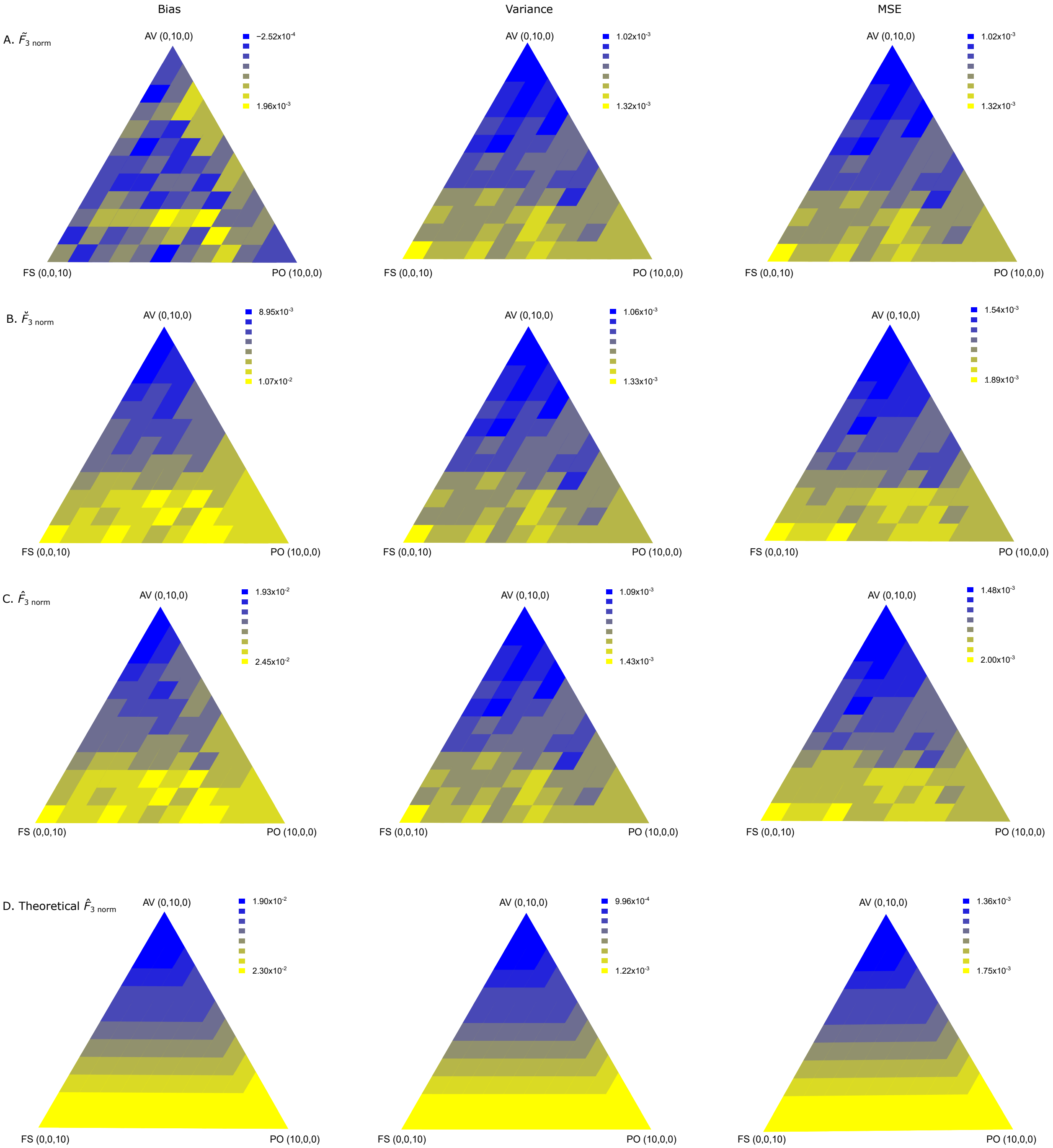

Figure S13: Theoretical vs. simulated normalized $F_3(A;B,C\,|\,A)$ bias, variance, and MSE when including various combinations of parent-offspring, avuncular and outbred full-sibling pairs. (Row A) Simulations of relative pairs used to compute bias, variance, and MSE of normalized $\widetilde{F}_3(A;B,C\,|\,A)$. (Row B) Simulations of relative pairs used to compute bias, variance, and MSE of normalized $\breve{F}_3(A;B,C\,|\,A)$. (Row C) Simulations of relative pairs used to compute bias, variance, and MSE of normalized $\widehat{F}_3(A;B,C\,|\,A)$. (Row D) Theoretically computed bias, variance, and MSE for normalized $\widehat{F}_3(A;B,C\,|\,A)$. The true value of normalized $F_3(A;B,C\,|\,A)$ is 0.116, computed for $J = 20$ loci.

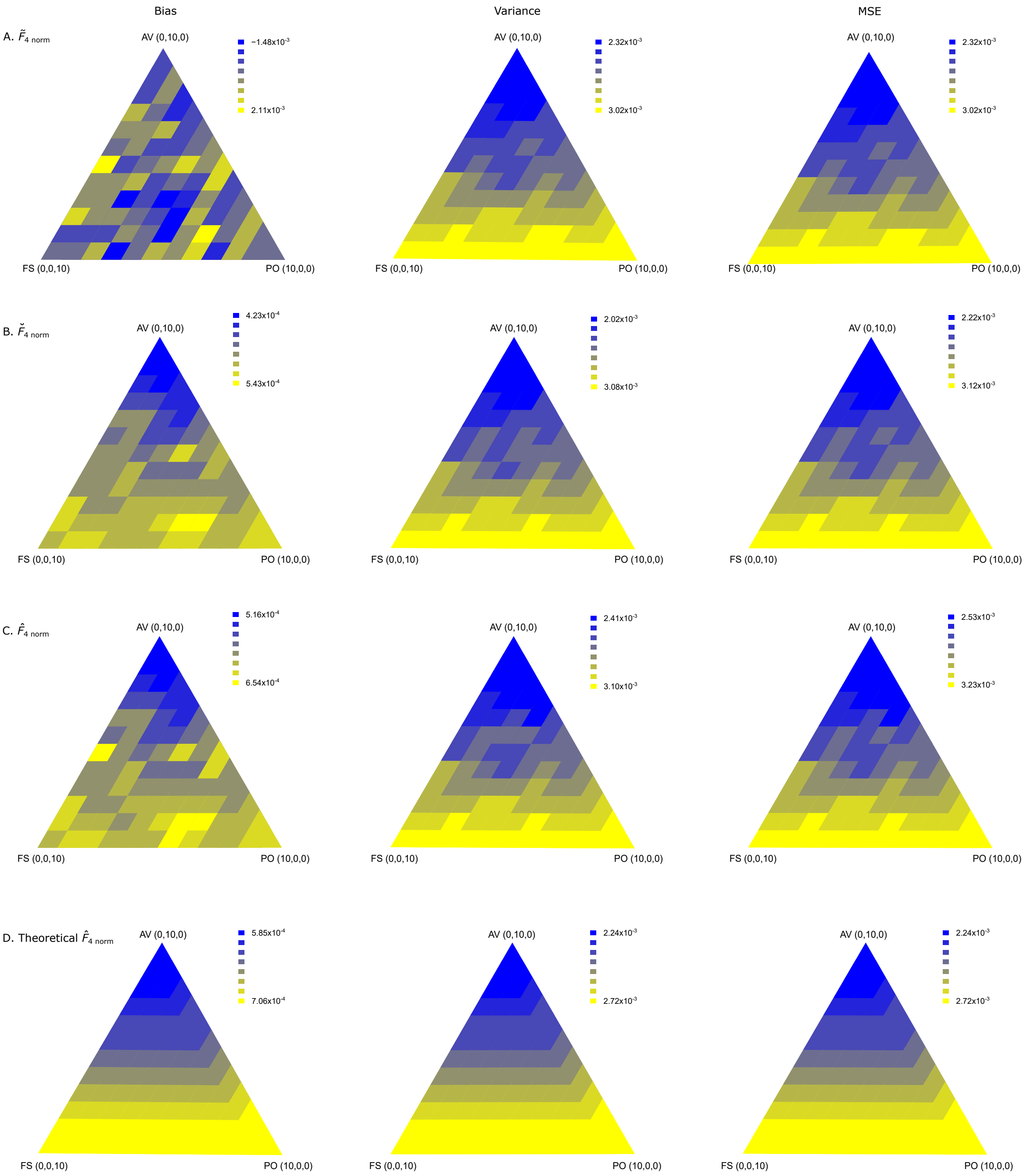

Figure S14: Theoretical vs. simulated normalized $F_4(A, B; C, D \mid A)$ bias, variance, and MSE when including various combinations of parent-offspring, avuncular and outbred full-sibling pairs. (Row A) Simulations of relative pairs used to compute bias, variance, and MSE of normalized $\widetilde{F}_4(A, B; C, D \mid A)$. (Row B) Simulations of relative pairs used to compute bias, variance, and MSE of normalized $\breve{F}_4(A, B; C, D \mid A)$. (Row C) Simulations of relative pairs used to compute bias, variance, and MSE of normalized $\widehat{F}_4(A, B; C, D \mid A)$. (Row D) Theoretically computed bias, variance, and MSE for normalized $\widehat{F}_4(A, B; C, D \mid A)$. The true value of normalized $F_4(A, B; C, D \mid A)$ is 0.052, computed for $J = 20$ loci.

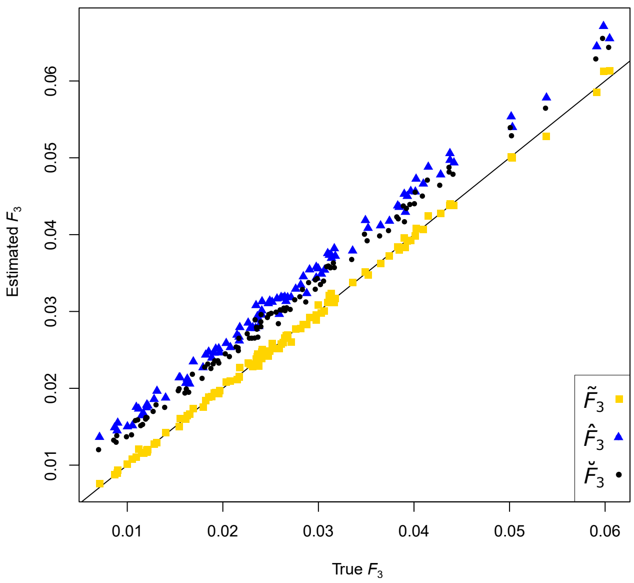

Figure S15: Comparison of true $F_3(A;B,C)$ to estimated $\widetilde{F}_3(A;B,C)$, $\breve{F}_3(A;B,C)$, and $\widehat{F}_3(A;B,C)$. Each dot represents the mean of 1000 simulations of parent offspring pairs used to compute $\widetilde{F}_3(A;B,C)$, $\breve{F}_3(A;B,C)$, and $\widehat{F}_3(A;B,C)$. Each simulation contains 50 parent offspring pairs.

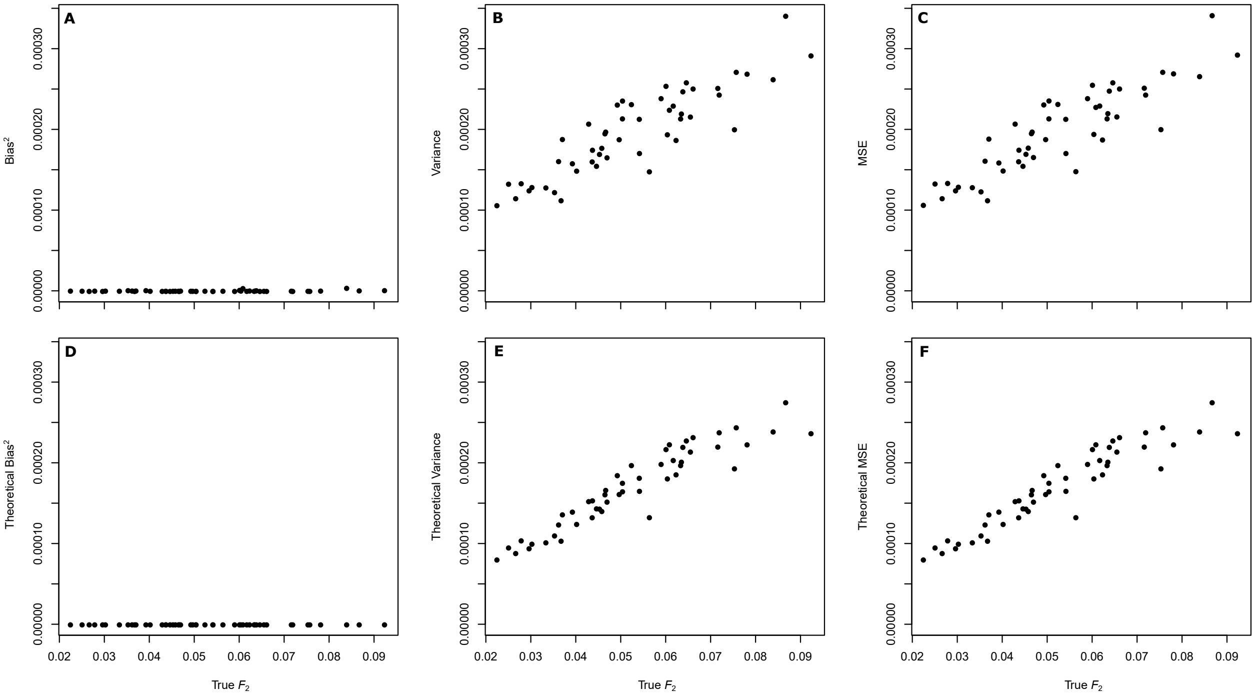

Figure S16: Simulated bias^2 (panel **A**), variance (panel **B**) and MSE (panel **C**) along with theoretical bias^2 (panel **D**), variance (panel **E**), and MSE (panel **F**) of $\widetilde{F}_2$ when the sample is composed of trios of full siblings. Each point represents data from 1000 simulations each and contains 20 full sibling trios.
